# Hypertension and diabetes prevalence, associated factors, care cascade, and quality of life in older adults: A cross-sectional population-based study in The Gambia, South Africa, and Zimbabwe

**DOI:** 10.1371/journal.pmed.1004785

**Published:** 2026-07-07

**Authors:** Anthony Muchai Manyara, Suad Abdullah, Ayaan Balshaf, Tadios Manyanga, Momodou Jallow, Etheldreda I. Yoliswa Madela, Hannah Wilson, Anya Burton, Farhanah Paruk, Chris Grundy, Camille Pearse, Tafadzwa Madanhire, Lucy Gates, Bilkish Cassim, Rashida A. Ferrand, Kate A. Ward, Celia L. Gregson

**Affiliations:** 1 Global Health and Ageing Research Unit, Bristol Medical School, University of Bristol, Bristol, United Kingdom; 2 The Health Research Unit Zimbabwe, Biomedical Research and Training Institute, Harare, Zimbabwe; 3 MRC Unit The Gambia, London School of Hygiene and Tropical Medicine, Banjul, The Gambia; 4 Department of Geriatrics, University of KwaZulu-Natal, Durban, KwaZulu-Natal, South Africa; 5 Division of Internal Medicine, University of KwaZulu-Natal, Durban, KwaZulu-Natal, South Africa; 6 Department of Infectious Disease Epidemiology and International Health, London School of Hygiene and Tropical Medicine, London, United Kingdom; 7 MRC Lifecourse Epidemiology Centre, Human Development and Health, University of Southampton, Southampton, United Kingdom; 8 Clinical Research Department, London School of Hygiene and Tropical Medicine, London, United Kingdom; Massachusetts General Hospital, UNITED STATES OF AMERICA

## Abstract

**Background:**

Hypertension and diabetes prevalence are increasing across Africa. We investigated the prevalence, associated factors, achievement of stages within the care cascade (diagnosis, treatment, control), and health-related quality of life (HRQoL) in three countries in Africa.

**Methods and findings:**

This cross-sectional study recruited adults aged ≥40 years in five settings: rural (*n* = 1,052) and urban (*n* = 1,218) The Gambia, rural (*n* = 948) and urban (*n* = 968) South Africa (SA), and urban (*n* = 1,110) Zimbabwe between 2022 and 2024. Data were collected using researcher-administered questionnaires and assessments. Hypertension and diabetes were defined using self-reported diagnosis, medication use, and blood pressure and glucose measurements. HRQoL was assessed using EuroQol-5 Dimension 5 Level questionnaire, with a minimally important difference (MID) defined as half a standard deviation (SD). Diabetes complications included neuropathy, cardiovascular disease, and kidney disease. Associations between hypertension and diabetes and risk factors were assessed using study site, age, sex, educational attainment, and wealth index-adjusted Generalised Linear Mixed Effects Models. Associations between care cascade stages and HRQoL were assessed using linear models.

Analysis included 5,296 adults, 53% female, and 52% age ≥60 years. Overall hypertension prevalence was 55.6% (95% confidence intervals [CI] 54.2%–56.9%); ranging from 39.6% (95% CI: 36.7–42.7) in rural The Gambia to 66.9% (64.1–69.7) in urban Zimbabwe. Overall, diabetes prevalence was 14.0% (13.1%–15.0%), ranging from 9.2% (7.6–11.0) in urban Zimbabwe to 19.4% (16.9–22.1) in rural SA. Both overweight and obesity, compared to normal weight, were associated with higher odds of hypertension (adjusted odds ratios: 1.73 (95% CI [1.47, 2.03]; *p* < 0.001) and 2.08 (95% CI [1.74, 2.49]; *p* < 0.001), respectively and diabetes (1.53 (95% CI [1.22, 1.91]; *p* < 0.001) and 2.12 (95% CI [1.67, 2.69); *p* < 0.001), respectively. The proportion with treated and controlled hypertension was 31.0% (913/2944), with 27.2% (800/2944) undiagnosed, and 26.0% (766/2944) treated but uncontrolled. Overall, 21.9% (161/735) had treated and controlled diabetes, whilst 49.7% (365/735) were undiagnosed, and 15.4% (113/735) were diagnosed and untreated. Underdiagnosis and inadequate treatment and control of both diseases were more common in men and in The Gambia. Overall, hypertension targets of 80-80-80% in diagnosis, treatment, and control were 72.8%(2144/2944), 78.3% (1679/2144), and 54.4% (913/1679), respectively. Diabetes targets of 80-80-80-60% in diagnosis, glucose control, hypertension control, and statin use were 50.8%(377/742), 65.7% (243/370), 60.4% (224/371), and 17.8% (67/377), respectively. For both disease targets, South Africa performed better than The Gambia and Zimbabwe. The overall mean±SD HRQoL utility score was 0.829 ± 0.107 (MID = 0.054). Compared to being nonhypertensive, having diagnosed and untreated hypertension was associated with a −0.07 (95%CI [−0.05, −0.08]; *p* < 0.001) lower HRQoL utility score. Compared to being nondiabetic, having treated and controlled diabetes was associated with lower HRQoL: −0.07 (95% CI [−0.01, −0.13]; *p* = 0.028) in The Gambia and −0.08 (95% CI [−0.03, −0.12]; *p* < 0.001) in Zimbabwe. Having ≥1 complication was associated with lower HRQoL: −0.04 (95% CI [−0.03, - 0.05]; *p* < 0.001). Study limitations are the cross-sectional design and reliance on single measurements of blood pressure and glucose concentrations.

**Conclusions:**

The high prevalence of hypertension and diabetes in mid-age and older adults in rural and urban Africa necessitates urgent diagnostic, preventive, and control interventions. This can include interventions targeted at obesity, screening of all adults aged ≥40 years, prompt and optimal treatment for those diagnosed, and ongoing monitoring to limit complications.

## Author summary

**Why was this study done?:**

- With changing lifestyles and increasing longevity, hypertension and diabetes prevalence are increasing across Africa, necessitating up-to-date evidence to inform health policy and practice.
- Most of the evidence on hypertension and diabetes in Africa focuses on younger adults (<50 years).
- Achievement of African hypertension targets (published by the World Hypertension League in 2022) and global diabetes targets (launched in 2022 by WHO) was poorly understood across Africa, and the relationship between care cascade stages (disease diagnosis, treatment, control) and HRQoL was understudied.

**What did the researchers do and find?:**

- Using data from a population-based cross-sectional study of adults aged ≥40 years in The Gambia, Zimbabwe, and South Africa, we assessed the prevalence of hypertension and diabetes, associated factors, proportions achieving the three stages of the care cascade, and associated HRQoL.
- We found a hypertension prevalence of 55.6% and diabetes of 14.0%, with only 31.0% having controlled hypertension and 21.9% controlled diabetes. Obesity was associated with higher odds of both diseases.
- Achievement of African hypertension targets of 80-80-80% in diagnosis, treatment, and control were 72.8%, 78.3%, and 54.4%, respectively; and global diabetes targets of 80-80-80-60% in diagnosis, glucose control, hypertension control, and statin use were 50.8%, 65.7%, 60.4%, and 17.8% respectively; with The Gambia and Zimbabwe having wider gaps in care than South Africa.
- Having diagnosed but untreated hypertension, compared to being nonhypertensive, was associated with lower HRQoL, as was the presence of diabetes complications.

**What do these findings mean?:**

- Interventions to reduce obesity are potentially an important strategy for preventing both diseases.
- There is an urgent need for realignment of health systems to prioritise screening for adults ≥40 years, coupled with appropriate strategies for management of common chronic diseases.
- Monitoring for secondary complications alongside the achievement of diabetes and hypertension control is needed.
- The main study limitations are the cross-sectional design and reliance on single measurements of blood pressure and glucose concentrations.

## Abstract 3

In a cross-sectional population-based study, Anthony Manyara and colleagues investigate the prevalence, associated factors, achievement of stages within the care cascade, and health-related quality of life in older adults in The Gambia, South Africa, and Zimbabwe.

## Introduction

The world is working to reduce by one-third the mortality attributed to the four major noncommunicable diseases (NCDs): cardiovascular disease, cancer, diabetes, and chronic respiratory disease [1]. Hypertension increases cardiovascular disease mortality and is commonly comorbid with diabetes [2]. In sub-Saharan Africa, approximately 2 million premature deaths are attributable to diabetes and hypertension complications annually; a number expected to increase [3]. The International Diabetes Federation (IDF) projects that by 2050, Africa will experience the greatest increase (+142%) compared to other global regions, in people living with diabetes, mainly due to changing lifestyle factors such as obesity, unhealthy diets, and low physical activity [4]. Similarly, the number of people with hypertension in Africa has doubled over the last three decades [5], and premature deaths due to hypertensive heart disease are predicted to increase [6]. Despite rising prevalence, nationally representative, up-to-date, and robust evidence on both diseases in Africa remains limited [4,5,7].

In 2024, the IDF estimated the age-standardised prevalence of diabetes in Africa as 5%, although available evidence suggests that prevalence is higher in older adults. A 2016 meta-analysis reported a 14% pooled prevalence of diabetes among adults aged ≥55 years in Africa [8]. Given the diverse diabetes phenotypes, a 2025 meta-analysis found a pooled prevalence of lean type 2 diabetes (individuals with a body mass index [BMI] of <25 kg.m^2^) of 39% in people with a mean age of 48 years [9]. For hypertension, a series of meta-analyses have reported the pooled prevalence of diabetes in different age groups in Africa: 57% in adults aged ≥50 years in 2019 [10]; 27% in rural and 34% in urban settings amongst West Africans of mean age 35–56 years [11]; and 58% in people living with diabetes, with a mean age of 58 years in 2023 [12].

A large unmet need remains in the diagnosis, treatment, and control of both conditions. The estimated total unmet need (sum of those not tested, tested but unaware of their diagnosis, aware of their diagnosis but untreated, and treated but with uncontrolled diabetes) for diabetes care in 12 African countries was approximately 80% as reported by Manne-Goehler and colleagues [13] and 62% in Kenya, Burkina Faso, Ghana and SA as reported by Wade and colleagues [14]; while for hypertension in sub-Saharan Africa, the unmet need was 87% for women and 91% for men [5]. However, most of these estimates are based on studies conducted at least a decade ago. There is a further need to track progress towards the World Hypertension League’s targets that aim to achieve, by 2030 in Africa: 80% diagnosis of hypertension in adults; 80% treatment of those diagnosed; and 80% control of those treated [15]. Similarly, progress remains to be determined towards the first-ever global targets for type 2 diabetes, adopted by WHO member states in 2022, which aim to be achieved by 2030: 80% diagnosed; among those diagnosed 80% with blood glucose controlled; among those diagnosed 80% with blood pressure controlled, and if age ≥40 years, 60% on statin medication [16].

Patient-reported outcome measures, such as health-related quality of life, are important indicators of treatment efficacy and burden based on patients’ experiences [17]. However, the association between hypertension or diabetes treatment, control, and quality of life remains understudied in Africa. While WHO STEPwise approach to NCD risk factor surveillance (STEPS) surveys provides important evidence on hypertension and diabetes prevalence and associated factors, most of the surveys were conducted a decade ago, limited to adults below 70 years, and lack data on quality of life [18].

This study aimed to add to the existing literature on hypertension and diabetes and fill knowledge gaps based on data from three countries representing the Western (The Gambia) and Southern Africa regions (Zimbabwe, South Africa) and different World Bank income groups: low-income (The Gambia), lower-middle income (Zimbabwe), and upper-middle income (South Africa) [19]. Specifically, this study aimed to answer the following questions: 1) What is the prevalence of hypertension, diabetes and comorbid diabetes and hypertension across urban and rural sites? 2) Which sociodemographic, household, and lifestyle factors are associated with hypertension and diabetes? 3) What is the achievement status on the stages of the hypertension and diabetes care cascades, including attainment of African hypertension targets and global targets for diabetes? and 4) What are the associations between different care cascade stages (diagnosis, treatment, and control) and health-related quality of life? Informed by literature, we hypothesised that socioeconomic (e.g., older age, high educational attainment), household (e.g., high household wealth), lifestyle (e.g., obesity) factors were associated with hypertension and diabetes [20,21]; and untreated and uncontrolled hypertension and diabetes were associated with lower quality of life [22,23].

## Methods

This study is reported using the STROBE (Strengthening the reporting of observational studies in epidemiology) checklist for cross-sectional studies (S1 Checklist) [24].

### Study design and population

This population-based cross-sectional study, conducted between 2022 and 2024, was embedded within a primary study of prevalent vertebral fractures and multimorbidity in adults aged ≥40 years in five sites: rural and urban The Gambia, rural and urban South Africa, and urban Zimbabwe (low population densities and poor road access prevented rural data collection in Zimbabwe). The study sites were representative of typical urban and rural sites and thus broadly representative of urban and rural sites in each country. A sample target of 504 women and 504 men in each site age ≥40 years (equally distributed across three age strata: 40–54, 55–69 and ≥70 years) provided ≥90% power (α = 0.05) to estimate with 2.5% precision, an outcome with 9% prevalence (e.g., vertebral fracture) in each site, and detect an odds ratio of 2.0 for an associated risk factor with 13% prevalence (e.g., HIV) [25]. Further details on the study are described in the published protocol [25].

Each of the five sites was divided into sampling blocks using satellite imagery and OpenStreetMap within the geographical information systems QGIS. These blocks were designed to be of similar sizes, with each one expected to have 18–30 residents who could participate in the study. The blocks were randomly selected, and field workers approached every house, enumerated residents, and invited those who were eligible to participate. Enumeration continued until the sample targets were achieved.

### Data collection procedures and definitions

Data were collected using validated and standardised procedures involving researcher-administered questionnaires (e.g., sociodemographic characteristics, self-reported disease diagnosis), physical assessments (e.g., blood pressure measures, anthropometry), and point-of-care blood tests for blood glucose. Participants brought their medications to the research clinic; the names and indications of the medications were recorded. Medications were classified and indications were reviewed and cleaned to indicate relevant diseases by two consultant physicians (EIYM, CLG).

#### Hypertension.

Blood pressure was measured on the upper, nondominant arm using an Omron blood pressure monitor (Omron Corporation, Kyoto, Japan) and an appropriately-sized cuff (mid-upper arm circumference was measured to inform cuff size), with participants seated in a chair after resting for at least 5 min [26]. Three blood pressure readings, each 5 min apart, were taken. Participants with high blood pressure readings, based on the lowest reading (i.e., ≥140 mmHg for systolic blood pressure (SBP) or ≥90 mmHg for diastolic blood pressure (DBP)), were referred to a local health facility for further management.

Hypertension was defined using one of the following: 1) previously diagnosed by a medical professional, 2) taking antihypertensives, or 3) the lowest of the three blood pressure measurements being ≥140 mmHg for SBP or ≥90 mmHg for DBP on the data collection day [27,26]. Undiagnosed hypertension was defined as participants who did not self-report a diagnosis nor were taking antihypertensives and had either SBP ≥ 140 mmHg or DBP ≥ 90 mmHg. Treated hypertension was defined as recorded use of antihypertensives, while controlled hypertension was defined as SBP < 140 mmHg and DBP < 90 mmHg. Hypertension medications were classified as calcium channel antagonists, angiotensin-converting-enzyme (ACE) inhibitors, angiotensin receptor blockers, alpha-2 adrenergic receptor agonists and beta-blockers (for hypertension indication), thiazide diuretics, hydrazinophthalazine, loop diuretics, and potassium-sparing diuretics (if given with a hypertension indication in conjunction with other antihypertensives) (Table E in S1 Appendix).

#### Diabetes.

A prior diabetes diagnosis was self-reported. The questionnaire was unable to differentiate between type 1 and type 2 diabetes. Type 2 diabetes was assumed for the study population, given the inclusion age and context; however, age and insulin use were reviewed to investigate the possible inadvertent inclusion of type 1 diabetes cases. A point-of-care finger prick was used to obtain a capillary whole blood sample for glucometer measurement of glucose levels (mmol/L). The times of the last intake of food and drink (excluding water) and the glucose test were recorded. Fasting blood glucose was defined as a ≥8-hour difference between these times, and a random blood glucose level as a <8-hour difference. Diabetes was defined using one of the following: 1) previous diagnosis by a medical professional, 2) taking diabetes medications, 3) a random blood glucose of ≥11.1 mmol/L, or 4) a fasting blood glucose of ≥7 mmol/L [28]. Where the time difference between last food/drink and glucose test was unclear, a random blood glucose was assumed. Undiagnosed diabetes was defined as participants who did not self-report a diagnosis, were not taking diabetes medications, and had an elevated blood glucose (as above). The validity of this approach was checked using the African Diabetes Risk Score (ADRS): a validated simple risk score for identifying undiagnosed diabetes in African populations that uses age, hypertension, and waist circumference [29]. Compared to those without diabetes, those categorised as having undiagnosed diabetes had a higher risk score in South Africa and Zimbabwe, where waist circumference was measured: mean ± standard deviation (SD) of 7.6 ± 6.0 versus 5.9 ± 5.8. Treated diabetes was defined as having any recorded diabetes medication, while controlled diabetes was defined by a random blood glucose <11.1 mmol/L or a fasting blood glucose <7 mmol/L. Participants with an elevated blood glucose were referred to a local health facility for management, while those with a low blood glucose (<3.7 mmol/L) were promptly given sugary food/drink and referred by the study clinician for clinical management.

Diabetes medications were categorised as Biguanides, sulfonylureas, and insulin (Table F in S1 Appendix); notably, no other diabetes medications were reported in these settings at the time of data collection. Similarly, two statin medications were documented (simvastatin and atorvastatin) and used to compute the proportions of participants living with diabetes who were using statins. Diabetes complications were defined based on self-reported diagnosis or use of medication with an indication for neuropathy, cardiovascular disease, or chronic kidney disease.

#### Sociodemographic, household, and lifestyle factors.

Sociodemographic and household factors were self-reported. Educational attainment and marital status were binarised to generate a single category risk factor no formal/primary level education versus secondary/tertiary level; single/widowed versus married/cohabiting). A household wealth index was computed from ownership of household assets using a principal component analysis as described previously [30], and binarised as low versus middle/high wealth. Food insecurity was assessed using five *Yes/No* questions taken from the Household Food Insecurity Access Scale, documenting food insufficiency in the last 4 weeks [31]. Food security was defined as responding *No* to all questions, and insecurity as responding *Yes* to any of the five questions.

Alcohol intake and tobacco use were self-reported and classified as current or former use/consumption and never used/consumed. Physical activity, assessed using the short-form of the International Physical Activity Questionnaire [32], was used to calculate Metabolic equivalent task (MET) minutes per week [33]. Low physical activity was defined as <3,000 MET-minutes per week, and moderate or high physical activity as ≥3,000 MET-minutes per week [34].

Height was measured using Seca 213 stadiometers and weight using Seca 803 scales. BMI was computed using height and weight measures and categorised as underweight (<18.5 kg.m^2^), normal (18.5–24.9 kg.m^2^), and overweight (25–29.9 kg.m^2^) and obese (≥30 kg.m^2^) [35]. Waist and hip circumference were measured using the Seca 201 measuring tape in South Africa and Zimbabwe; it was omitted in The Gambia for cultural reasons, as advised by community members. Waist circumference was taken as the minimum circumference between the bottom of the rib cage and the iliac crests (hip bones), while hip circumference was taken at the widest point of the buttocks. An average of two measurements was used to calculate the waist-to-hip ratio (WHR). A high WHR was defined as ≥0.90 in men and ≥0.85 in women [36].

#### Quality of life.

Health-related quality of life (HRQoL) was assessed by researcher-administered questionnaire using the visual analogue scale (VAS) and EuroQol-5 Dimension 5 Level (EQ-5D-5L) [37] converted to a utility score based on the Zimbabwe value set [38]. Sensitivity analyses used the Ghanian value set [39].

### Ethical approvals

Before data collection, all participants provided written informed consent or, for those with cognitive impairment, assent through a proxy (family member, carer, and friend). Cognitive impairment was implied for participants who could not understand, retain, or weigh up information provided or communicate their decision based on this information. Ethical approval was obtained in all the study countries. The Gambia: The Gambia Government/MRC Unit The Gambia, LSHTM Scientific Coordinating Committee and Ethics Committee (22/04/2021 ref 22975); Ministry of Health (20/08/2021 ref DDHS/AD/2021/08(MTN27)). Zimbabwe: The Medical Research Council of Zimbabwe (14/07/2021 ref MRCZ/A/2706). South Africa: The University of KwaZulu-Natal’s Biomedical Research Ethics Committee (BREC, 21/08/2021 BREC/00002513/2021).

### Statistical analysis

The analyses were pre-planned as part of an internship in May 2024. Data-driven changes to the analyses (emerging from health-related quality of life-related analysis) were incorporated in June 2025. Changes to the analyses following peer review were incorporated between January and April 2026. Analyses were performed using R statistical software (version 4.3.1) [40]. Continuous variables were summarised as means (±SD), and categorical variables as counts (and percentages). Prevalence estimates for hypertension, diabetes, and hypertension-diabetes comorbidity were calculated by study site, sex, and age group. The Clopper-Pearson exact binomial method (using the binom.test() function [40]) was used to compute 95% confidence intervals (CI) for all prevalence estimates.

Associations between hypertension and diabetes and sociodemographic and household characteristics (age, sex, marital status, educational attainment, food insecurity, and wealth index) and lifestyle factors (BMI, WHR, tobacco use, alcohol consumption, and physical activity) were assessed for each disease using Generalised Linear Mixed Effects Models (GLMM) with study site as a fixed effect and a random intercept for sampling block, nested within sampling area, nested within site, to account for the hierarchical sampling structure. Model estimation used the Laplace approximation with the BOBYQA optimiser set to a maximum of 200,000 function evaluations to facilitate convergence using the ‘lme4’ package [41].

The first model was adjusted for study site, the second adjusted for site, age (as a continuous variable) and sex, and the third adjusted for confounders. The identification of confounders was based on a conceptual framework used by similar studies in Malawi [20] and The Gambia [21]. This framework categorises risk factors for hypertension or diabetes into distal factors (age, sex, urban or rural residence, educational attainment, occupation, and asset ownership) and proximal factors (e.g., smoking, physical activity) and assumes that distal factors influence proximal factors; hence, distal factors are potential confounders between the proximal risk factors and outcomes of interest [20,21]. Therefore, an adjustment was made in the third model for available distal factors: study site, age, sex, educational attainment, and wealth index categories. Data on employment did not distinguish between those retired and unemployed in this older population; therefore, occupation-related adjustments were not made. Given the unexpected protective association between tobacco use and alcohol consumption and diabetes, further adjustments and disaggregation were explored. Furthermor, in the third model, additional adjustments were made for BMI and tobacco use (when alcohol was the exposure) and alcohol consumption (when tobacco use was the exposure). After these adjustments, alcohol use still had a protective association against diabetes, and hence the association was explored in only those with diabetes with the exposure group being those who were newly diagnosed with diabetes (*n* = 365), given that those already diagnosed may have stopped alcohol consumption as part of lifestyle modification. Following peer review suggestion, a sensitivity analyses was explored using continuous variables (for age, wealth index, BMI, and MET minutes per week) and categories as captured in the questionnaire without binarisation for education, marital status, and food insecurity.

The cascades of care for hypertension, diabetes, and diabetes-hypertension comorbidity were presented in stacked bar graphs to show the proportion of adults undiagnosed, diagnosed but untreated, treated but uncontrolled, and controlled in all people with a condition and were disaggregated by the five sites, and sex. A similar approach was used to present the progress towards the African hypertension and global diabetes targets (albeit without sex and age disaggregation, given a reduced sample size). Using the previously described definitions, descriptive analyses (counts and percentages) were conducted for hypertension and diabetes medications, and results for diabetes complications were presented in the text or tables. Associations between the different care cascade categories (nonhypertensive/nondiabetic, treated and controlled, treated and uncontrolled, diagnosed and untreated, and undiagnosed) and HRQoL Zimbabwe value set utility score (outcome), were determined using generalised linear models (GLMs) for each disease in the combined study population and also by country, given the small sample sizes after rural-urban disaggregation. The same analysis was repeated using the VAS as the outcome, and sensitivity analysis used the utility scores from the Ghanaian value set [39] following peer review suggestions. Data-driven analyses were performed using GLMs to understand the associations between care cascade categories and HRQoL, the relationship between these categories and the number of prescribed medications for each disease and complications (specifically for diabetes). Overall, CI were used to infer evidence of between-group differences. A minimally important difference (MID) in HRQoL was defined as half a SD [42], and where CI of differences were computed, the lower confidence bound was used to infer achievement of the MID. Comparisons between being nondiabetic or nonhypertensive and care cascade categories were reported when MID was reached. Overall, apart from waist circumference, which was not measured in The Gambia, missingness in all other variables ranged from 0% to 7.5%, and no further analyses were conducted to handle missingness.

## Results

### Participants and descriptive data

**Fig 1:**
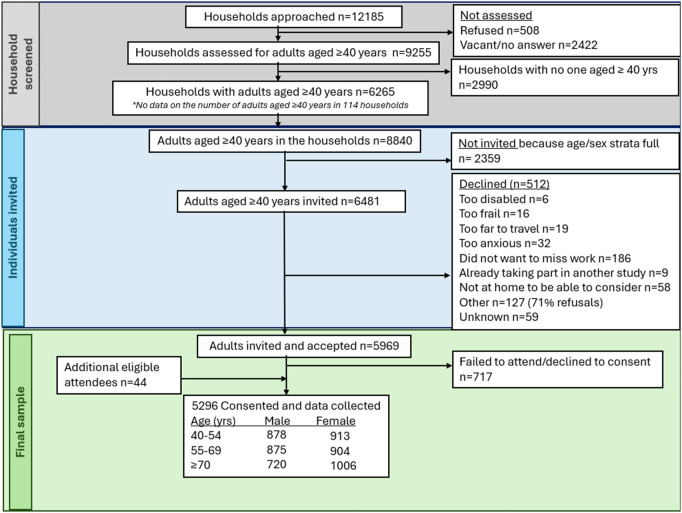
Participant flow diagram. The 44 additional eligible attendees were members of the same household who accompanied the invited participants in a caregiving capacity.

In total, 12,185 households were enumerated, identifying 8,840 eligible adults, of whom 6,481 were invited, and 5,296 (81.7%) participated and provided informed consent or assent (either directly or via proxy) and are included in the analysis (Fig 1). Of the included participants, 99.3% (5,259) had blood pressure and 98.3% (5,208) blood glucose measurements available.

Overall, 52% of participants were female. Low educational attainment was most common in The Gambia, whilst food insecurity was most common in South Africa. Obesity was most common in South Africa, and in both South Africa and Zimbabwe, more than half of the participants had a high WHR (Table 1).

**Table 1:** Sociodemographic, household, and lifestyle characteristics across the five sites.

	Overall, *n* = 5,296	The Gambia Rural, *n* = 1,052	The Gambia Urban, *n* = 1,218	South Africa Rural, *n* = 948	South Africa Urban, *n* = 968	Zimbabwe Urban, *n* = 1,110
Sex						
Women	2,823/5,296 (53.3%)	547/1,052 (52.0%)	671/1,218 (55.1%)	513/948 (54.1%)	520/968 (53.7%)	572/1,110 (51.5%)
Age group						
40–54 years	1,791/5,296 (33.8%)	344/1,052 (32.7%)	398/1,218 (32.7%)	340/948 (35.9%)	348/968 (36.0%)	361/1,110 (32.5%)
55–69 years	1,779/5,296 (33.6%)	341/1,052 (32.4%)	386/1,218 (31.7%)	345/948 (36.4%)	344/968 (35.5%)	363/1,110 (32.7%)
≥70 years	1,726/5,296 (32.6%)	367/1,052 (34.9%)	434/1,218 (35.6%)	263/948 (27.7%)	276/968 (28.5%)	386/1,110 (34.8%)
Education attainment						
Primary/No education	2,984/5,296 (56.3%)	931/1,052 (88.5%)	913/1,218 (75.0%)	504/948 (53.2%)	239/968 (24.7%)	397/1,110 (35.8%)
Secondary and above	2,262/5,296 (42.7%)	114/1,052 (10.8%)	276/1,218 (22.7%)	442/948 (46.6%)	724/968 (74.8%)	706/1,110 (63.6%)
*Missing*	*50/5,296 (0.9%)*	*7/1,052 (0.7%)*	*29/1,218 (2.4%)*	*2/948 (0.2%)*	*5/968 (0.5%)*	*7/1,110 (0.6%)*
Marital status						
Married	2,976/5,296 (56.2%)	868/1,052 (82.5%)	923/1,218 (75.8%)	345/948 (36.4%)	260/968 (26.9%)	580/1,110 (52.3%)
Single	1,248/5,296 (23.6%)	30/1,052 (2.9%)	55/1,218 (4.5%)	442/948 (46.6%)	559/968 (57.7%)	162/1,110 (14.6%)
Widowed	1,062/5,296 (20.1%)	154/1,052 (14.6%)	236/1,218 (19.4%)	160/948 (16.9%)	144/968 (14.9%)	368/1,110 (33.2%)
*Missing*	*10/5,296 (0.2%)*	*0/1,052 (0.0%)*	*4/1,218 (0.3%)*	*1/948 (0.1%)*	*5/968 (0.5%)*	*0/1,110 (0.0%)*
Household wealth index categories						
Low	2,116/5,296 (40.0%)	530/1,052 (50.4%)	420/1,218 (34.5%)	467/948 (49.3%)	323/968 (33.4%)	376/1,110 (33.9%)
Mid	2,017/5,296 (38.1%)	406/1,052 (38.6%)	393/1,218 (32.3%)	314/948 (33.1%)	534/968 (55.2%)	370/1,110 (33.3%)
High	1,163/5,296 (22.0%)	116/1,052 (11.0%)	405/1,218 (33.3%)	167/948 (17.6%)	111/968 (11.5%)	364/1,110 (32.8%)
Food insecurity	2,350/5,296 (44.4%)	386/1,052 (36.7%)	389/1,218 (31.9%)	573/948 (60.4%)	657/968 (67.9%)	345/1,110 (31.1%)
*Missing*	*7/5,296 (0.1%)*	*0/1,052 (0.0%)*	*3/1,218 (0.2%)*	*1/948 (0.1%)*	*3/968 (0.3%)*	*0/1,110 (0.0%)*
BMI categories						
Underweight (<18.5 kg.m^2^)	369/5,296 (7.0%)	160/1,052 (15.2%)	62/1,218 (5.1%)	28/948 (3.0%)	44/968 (4.5%)	75/1,110 (6.8%)
Normal (18.5–24.9 kg.m^2^)	2,163/5,296 (40.8%)	617/1,052 (58.7%)	523/1,218 (42.9%)	275/948 (29.0%)	271/968 (28.0%)	477/1,110 (43.0%)
Overweight ((≥25–29.9 kg.m^2^)	1,284/5,296 (24.2%)	209/1,052 (19.9%)	363/1,218 (29.8%)	220/948 (23.2%)	192/968 (19.8%)	300/1,110 (27.0%)
Obese (≥30 kg.m^2^)	1,399/5,296 (26.4%)	59/1,052 (5.6%)	252/1,218 (20.7%)	393/948 (41.5%)	444/968 (45.9%)	251/1,110 (22.6%)
*Missing*	*81/5,296 (1.5%)*	*7/1,052 (0.7%)*	*18/1,218 (1.5%)*	*32/948 (3.4%)*	*17/968 (1.8%)*	*7/1,110 (0.6%)*
High waist-to-hip ratio^‡^	1,665/5,296 (31.4%)	0/1,052 (0.0%)	0/1,218 (0.0%)	551/948 (58.1%)	553/968 (57.1%)	561/1,110 (50.5%)
*Missing*	*2,322/5,296 (43.8%)*	*1,052/1,052 (100.0%)*	*1,218/1,218 (100.0%)*	*40/948 (4.2%)*	*8/968 (0.8%)*	*4/1,110 (0.4%)*
Low physical activity^ꞙ^	3,710/5,296 (70.1%)	572/1,052 (54.4%)	1,080/1,218 (88.7%)	638/948 (67.3%)	627/968 (64.8%)	793/1,110 (71.4%)
*Missing*	*396/5,296 (7.5%)*	*31/1,052 (2.9%)*	*49/1,218 (4.0%)*	*93/948 (9.8%)*	*203/968 (21.0%)*	*20/1,110 (1.8%)*
Alcohol consumption^†^	839/5,296 (15.8%)	0/1,052 (0.0%)	6/1,218 (0.5%)	249/948 (26.3%)	391/968 (40.4%)	193/1,110 (17.4%)
*Missing*	*21/5,296 (0.4%)*	*2/1,052 (0.2%)*	*6/1,218 (0.5%)*	*2/948 (0.2%)*	*7/968 (0.7%)*	*4/1,110 (0.4%)*
Tobacco use^†^	1,171/5,296 (22.1%)	245/1,052 (23.3%)	200/1,218 (16.4%)	233/948 (24.6%)	303/968 (31.3%)	190/1,110 (17.1%)
*Missing*	*11/5,296 (0.2%)*	*1/1,052 (0.1%)*	*4/1,218 (0.3%)*	*3/948 (0.3%)*	*3/968 (0.3%)*	*0/1,110 (0.0%)*

Missingness was below 1% for the majority of variables in italic. † Use refers to current or former use.

‡ ≥0.90 in men and ≥0.85 in women.

ꞙ <3,000 Metabolic equivalent (METS) minutes per week.

BMI, body mass index.

### Prevalence of hypertension and diabetes

**Fig 2:**
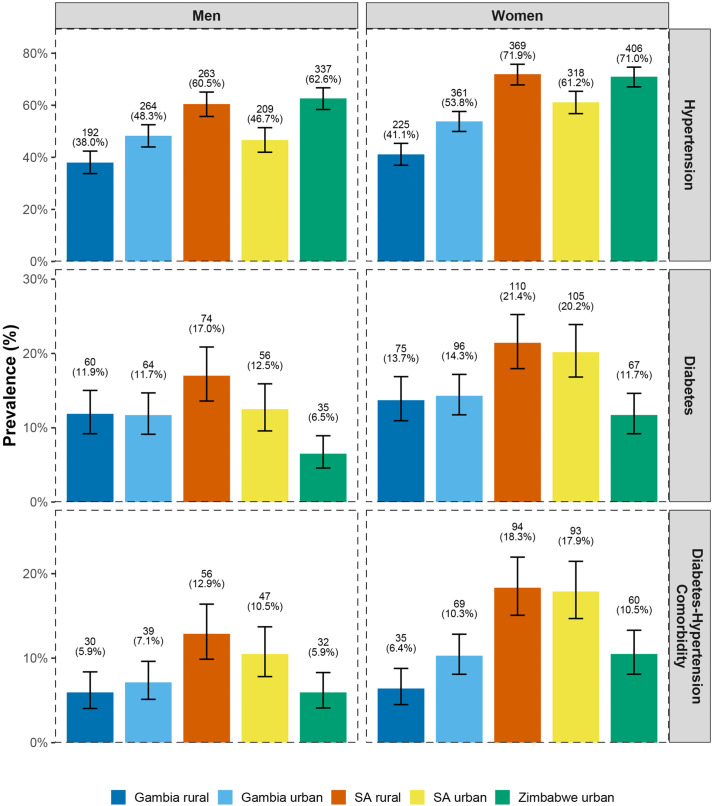
Prevalence of hypertension, diabetes, and diabetes-hypertension comorbidity across the five studies by sex. Also see Table A in S1 Appendix. SA: South Africa.

In total, the prevalence of hypertension was 55.6% (95% CI 54.2%–56.9%). Across study sites, prevalence was lowest in rural The Gambia (39.6% (95% CI: 36.7–42.7)); >50% in urban The Gambia (51.3% (48.5–54.2)) and urban South Africa (54.4% (51.3–57.6)); and >60% in rural South Africa (66.7% (63.6–69.7)) and urban Zimbabwe (66.9% (64.1–69.7)). The prevalence was higher among women in urban and rural South Africa and urban Zimbabwe (Fig 2, Table A in S1 Appendix). Across all study sites, prevalence was higher in the 40–54 age group compared to the ≥70 years age group (Fig A in S1 Appendix).

Overall, the prevalence of diabetes was 14.0% (95% CI: 13.1%–15.0%). The overall prevalence was similar for the rural (12.8% (10.9–15.0)) and urban (13.1% (11.4–15.2)) The Gambia, lower than for the rural (19.4% (16.9–22.1)) and urban (16.6% (14.3–19.1)) South Africa, and higher than in urban Zimbabwe (9.2% (7.6–11.0)). Prevalence was higher in women in urban South Africa and Zimbabwe (Fig 2, Table A in S1 Appendix). Prevalence was higher in older age groups and both sexes in all sites, except for men in urban Zimbabwe and women in urban The Gambia (Fig A in S1 Appendix). In all three countries, the prevalence of diabetes-hypertension comorbidity was 10.5% (9.7%–11.3%). Prevalence was higher in rural (15.8% (13.6–18.3)) and urban (14.5% (12.3–16.8)) South Africa and lower in the other study sites albeit with overlapping CI in urban The Gambia and Zimbabwe: 6.2% (4.8–7.8) in rural The Gambia, 8.9% (7.3–10.6) in urban The Gambia, and 8.3% (6.7–10.1) in urban Zimbabwe. The prevalence was higher among women in urban South Africa (Fig 2, Table A in S1 Appendix)

### Factors associated with hypertension and diabetes

Independent of study site, age, educational attainment, and wealth index, being female was associated with both hypertension and diabetes (aOR 1.39, 95% CI [1.23, 1.57]; *p* < 0.001) and (aOR 1.41, 95% CI [1.19, 1.67]; *p* < 0.001), respectively (Table 2).

**Table 2:** Pooled associations between diabetes and hypertension and sociodemographic, household, and lifestyle factors.

Characteristic	Hypertension						Diabetes					
	Model 1		Model 2		Model 3		Model 1		Model 2		Model 3	
	OR (95% CI)	*P*-value	OR (95% CI)	*P*-value	OR (95% CI)	*P*-value	OR (95% CI)	*P*-value	OR (95% CI)	*P*-value	OR (95% CI)	*P*-value
**Sociodemographic and household factors** *Reference category for each factor is to the right, and the odds shown relate to the factor on the left*												
Women vs. Men	1.45 (1.30;1.63)	<0.001	1.40 (1.24;1.58)	<0.001	1.39 (1.23;1.57)	<0.001	1.50 (1.27;1.77)	<0.001	1.44 (1.22;1.71)	<0.001	1.41 (1.19;1.67)	<0.001
55-69 vs. 40–54 years	2.75 (2.39;3.17)	<0.001	2.77 (2.40;3.19)	<0.001	2.71 (2.34;3.13)	<0.001	1.92 (1.55;2.38)	<0.001	1.93 (1.56;2.39)	<0.001	1.84 (1.48;2.30)	<0.001
≥70 vs. 40–54 years	6.81 (5.82;7.96)	<0.001	6.71 (5.74;7.85)	<0.001	6.59 (5.58;7.79)	<0.001	2.45 (1.98;3.03)	<0.001	2.36 (1.91;2.93)	<0.001	2.24 (1.79;2.82)	<0.001
Widowed vs. Married^a^	2.47 (2.10;2.91)	<0.001	1.16 (0.96;1.40)	0.120	1.18 (0.97;1.42)	0.091	1.24 (1.01;1.52)	0.041	0.83 (0.66;1.04)	0.109	0.85 (0.67;1.07)	0.167
Single vs. Married^a^	0.63 (0.54;0.75)	<0.001	0.84 (0.70;1.00)	0.049	0.84 (0.71;1.01)	0.059	0.51 (0.40;0.64)	<0.001	0.54 (0.42;0.69)	<0.001	0.55 (0.43;0.71)	<0.001
Low vs. High education	2.13 (1.85;2.44)	<0.001	1.05 (0.90;1.23)	0.505	1.07 (0.92;1.26)	0.385	1.58 (1.30;1.91)	<0.001	1.16 (0.95;1.43)	0.152	1.19 (0.97;1.47)	0.098
Low vs. High wealth index	0.86 (0.74;1.01)	0.068	0.86 (0.73;1.02)	0.079	0.86 (0.73;1.03)	0.094	0.74 (0.59;0.93)	0.010	0.76 (0.60;0.95)	0.018	0.77 (0.61;0.97)	0.029
Middle vs. High wealth index	0.98 (0.84;1.15)	0.819	0.92 (0.77;1.09)	0.324	0.93 (0.78;1.10)	0.399	0.82 (0.66;1.03)	0.086	0.82 (0.65;1.03)	0.082	0.82 (0.65;1.03)	0.095
Food insecure vs. Secure	0.92 (0.82;1.04)	0.198	0.96 (0.85;1.10)	0.583	0.97 (0.85;1.10)	0.615	0.99 (0.83;1.18)	0.940	1.02 (0.86;1.22)	0.809	1.05 (0.88;1.26)	0.604
**Lifestyle factors**												
BMI ≤ 18.5 vs. > 18.5–24.9 kg.m^2^	0.67 (0.53;0.85)	<0.001	0.57 (0.44;0.74)	<0.001	0.58 (0.45;0.75)	<0.001	0.64 (0.42;0.99)	0.043	0.61 (0.39;0.94)	0.024	0.62 (0.40;0.95)	0.030
BMI 25–29.9 vs. > 18.5–24.9 kg.m^2^	1.61 (1.39;1.87)	<0.001	1.73 (1.48;2.03)	<0.001	1.73 (1.47;2.03)	<0.001	1.55 (1.25;1.93)	<0.001	1.53 (1.23;1.91)	<0.001	1.53 (1.22;1.91)	<0.001
BMI ≥ 30 vs. > 18.5–24.9 kg.m^2^	1.97 (1.69;2.30)	<0.001	2.10 (1.76;2.52)	<0.001	2.08 (1.74;2.49)	<0.001	2.21 (1.78;2.73)	<0.001	2.13 (1.68;2.70)	<0.001	2.12 (1.67;2.69)	<0.001
High vs. Low WHR*^c^	1.60 (1.38;1.87)	<0.001	1.15 (0.97;1.36)	0.100	1.14 (0.96;1.35)	0.136	2.83 (2.21;3.61)	<0.001	2.42 (1.89;3.11)	<0.001	2.38 (1.85;3.05)	<0.001
Low vs. High physical activity^d^	1.59 (1.37;1.84)	<0.001	1.06 (0.91;1.25)	0.433	1.04 (0.89;1.22)	0.613	1.28 (1.03;1.60)	0.026	1.07 (0.85;1.34)	0.563	1.04 (0.83;1.31)	0.715
Current/Former tobacco user vs. never user	0.60 (0.53;0.69)	<0.001	0.76 (0.65;0.90)	0.001	0.87 (0.73;1.03)^†^	0.109	0.57 (0.46;0.71)	<0.001	0.71 (0.56;0.90)	0.005	0.88 (0.68;1.14)^†^	0.325
Alcohol consumption vs. nonconsumption	0.52 (0.44;0.62)	<0.001	0.92 (0.76;1.11)	0.370	1.14 (0.93;1.40)^†^	0.219	0.31 (0.23;0.42)	<0.001	0.41 (0.30;0.56)	<0.001	0.44 (0.32;0.61)^†‡^	<0.001

*P-values were derived from Wald z-tests.*

Model 1: Adjusted for site only.

Model 2: Adjusted for site, sex, and age.

Model 3: Adjusted for site, sex, age, household wealth index, and educational attainment.

All models include a random intercept for block nested within sampling area nested within site.

†Additional adjustment for BMI and alcohol consumption when tobacco use is the exposure and tobacco use when alcohol consumption is the exposure in Model 3.

‡Analysis in only those with diabetes, the site, sex, age, household wealth index, and educational attainment odds ratio in the newly diagnosed (*n* = 365) was 3.28 (1.60;6.76).

*Based on data from South Africa and Zimbabwe only.

^a^Married includes cohabiting, and single includes divorced and separated.

^b^Low education is defined as no formal education and primary level education attainment.

^c^High WHR defined as ≥0.90 in men and ≥0.85 in women.

^d^Low physical activity is defined as <3,000 Metabolic Equivalents minutes/week.

Independent of study site, sex, educational attainment, and wealth index, being 55–69 years, compared to being 40–54 years, was associated with higher odds of both hypertension (aOR 2.71, 95% CI [2.34, 3.13]; *p* < 0.001) and diabetes (aOR 1.84, 95% CI [1.48, 2.30]; *p* < 0.001). Similarly, being ≥70 years, compared to being 40–54 years, was associated with both hypertension (aOR 6.59, 95% CI [5.58, 7.79]; *p* < 0.001) and diabetes (aOR 2.24, 95% CI [1.79, 2.82]; *p* < 0.001) (Table 2). Independent of study site, sex, and educational attainment, having low household wealth, compared to high household wealth, was associated with lower odds of diabetes (aOR 0.77, 95% CI [0.61, 0.97]; *p* = 0.029) (Table 2). Independent of study site, sex, age, educational attainment, and wealth index, being single compared to being married was associated with lower odds of diabetes (aOR 0.55, 95% CI [0.43, 0.71]; *p* < 0.001) (Table 2).

Furthermore, independent of study site, sex, age, educational attainment and wealth index, being overweight or obese, compared to having normal BMI, were both associated with higher odds of hypertension (aOR 1.73, 95% CI [1.47, 2.03]; *p* < 0.001) and (aOR 2.08, 95% CI [1.74, 2.49]; *p* < 0.001), respectively, and diabetes (aOR 1.53, 95% CI [1.22, 1.91]; *p* < 0.001) and (aOR 2.12, 95% CI [1.67, 2.69]; *p* < 0.001), respectively (Table 2). A high WHR was associated with diabetes (aOR 2.38, 95% CI [1.85, 3.05]; *p* < 0.001) (Table 2).

Without binarising categorical variables, compared with never married, being currently married, divorced, or widowed was associated with hypertension and diabetes after adjusting for study site, sex, age, educational attainment, and wealth index (Table B in S1 Appendix).

### Hypertension and diabetes care cascades

For hypertension, the overall proportions were 31.0% (913/2944) for treatment and control; 26.0% (766/2,944) for treatment without control; 15.8% (465/2,944) for diagnosis without treatment; and 27.2% (800/2,944) for undiagnosed hypertension (Table C in S1 Appendix). Overall, 72.8% (2,144/2,944) were diagnosed and 57.0% (1,679/2,944) treated.

**Fig 3:**
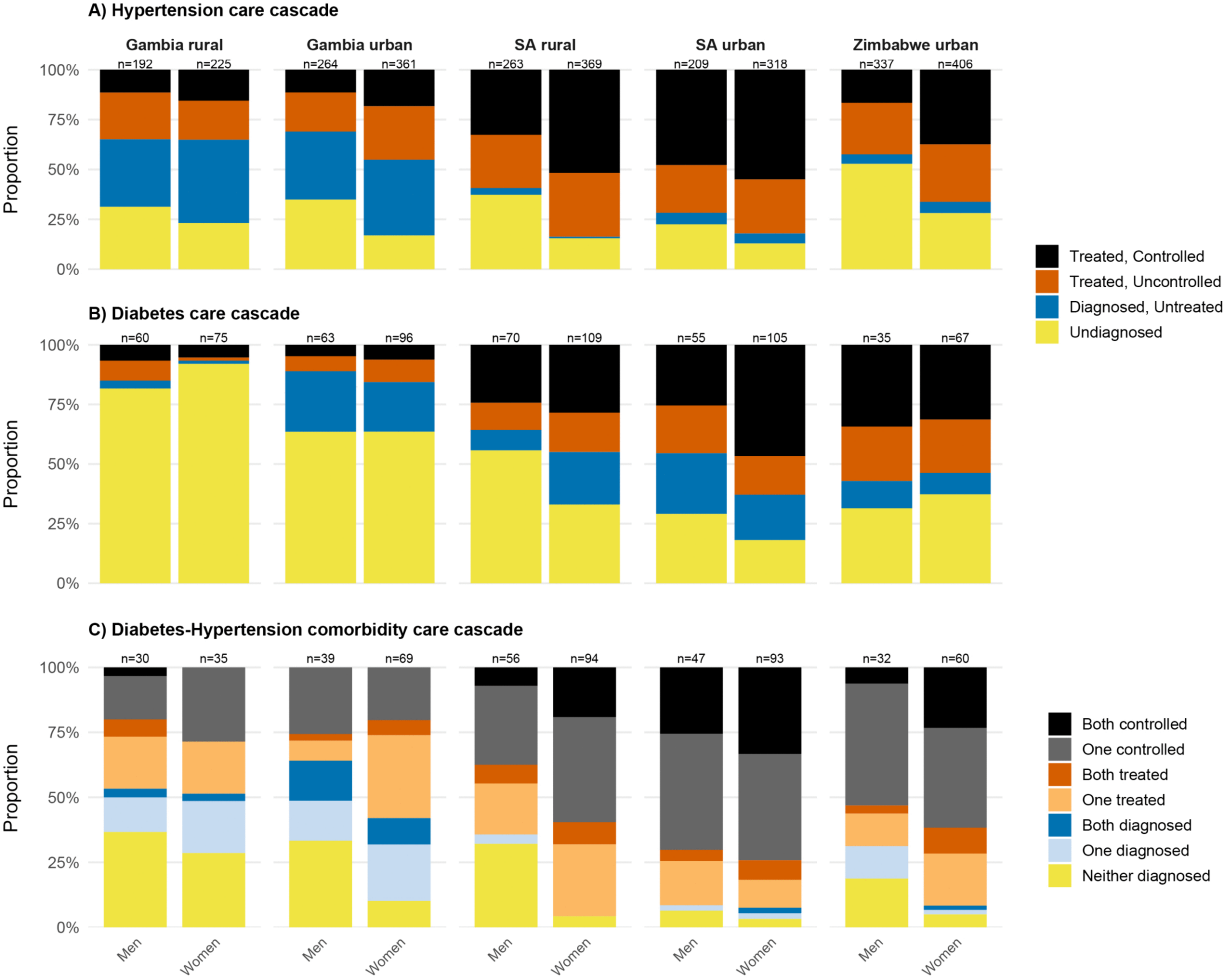
Proportions of people in each stage of the care cascade for hypertension, diabetes, and diabetes-hypertension comorbidity across the five sites, by sex. SA: South Africa.

Undiagnosed hypertension was more common among men than women, 37.5% versus 19.4%; this trend was also observed in all sites (Fig 3, Table D in S1 Appendix). Rural and urban The Gambia had the lowest proportions of hypertension treatment and control: <20% in both sexes (Fig 3, Table C in S1 Appendix).

For diabetes, the overall proportions were 21.9% 161/735) for treatment and control; 13.1% (96/735) for treatment without control; 15.4% (113/735) for diagnosis without treatment; and 49.7% (365/735) for undiagnosed hypertension (Table C in S1 Appendix). Overall, 50.3% (370/735) were diagnosed and 35.0% (257/735) treated.

Undiagnosed diabetes was more common in men than women: 54.8% versus 46.5% (Fig 3, Table D in S1 Appendix).

For those with comorbid hypertension and diabetes, the proportions were 14.8% (82/555) for both conditions controlled; 34.4% (191/555) for one controlled; 6.3% (35/555) for both treated; 19.6% (109/555) for one treated; 3.2% (18/555) for both diagnosed; 7.6% (42/555) for one diagnosed; and 14.1% (78/555) for neither diagnosed (Table C in S1 Appendix). Control of one or both conditions was more common in South Africa and Zimbabwe (Fig 3, Table D in S1 Appendix).

The number of antihypertensives taken ranged from 1 to 6 per person, with a mean±SD of 1.5 ± 0.8, varying from 1.2 ± 0.4 in The Gambia to 1.6 ± 0.9 in South Africa. The most common antihypertensive classes in all countries were thiazide diuretics (taken by 50.2% with treated hypertension) and calcium channel antagonists (36.1%), with the latter being more commonly used in Zimbabwe (57.5%) (Table G in S1 Appendix). The number of diabetes medicines ranged from 1 to 3, with an overall mean of 1.2 ± 0.4, which was similar in all three countries. Overall, Biguanides were the most common diabetes medication (70.8%) (Table G in S1 Appendix). Of the 19 (7.2% of all on diabetes medication) using insulin, only 10 (1.3% of all people with diabetes) were using insulin without reporting Biguanides or sulfonylurea use, corresponding to 3.8% of all patients with diabetes on medication. The median age of these 10 participants was 65 years, with just five younger than 65 years.

### World Hypertension League’s targets for Africa

**Fig 4:**
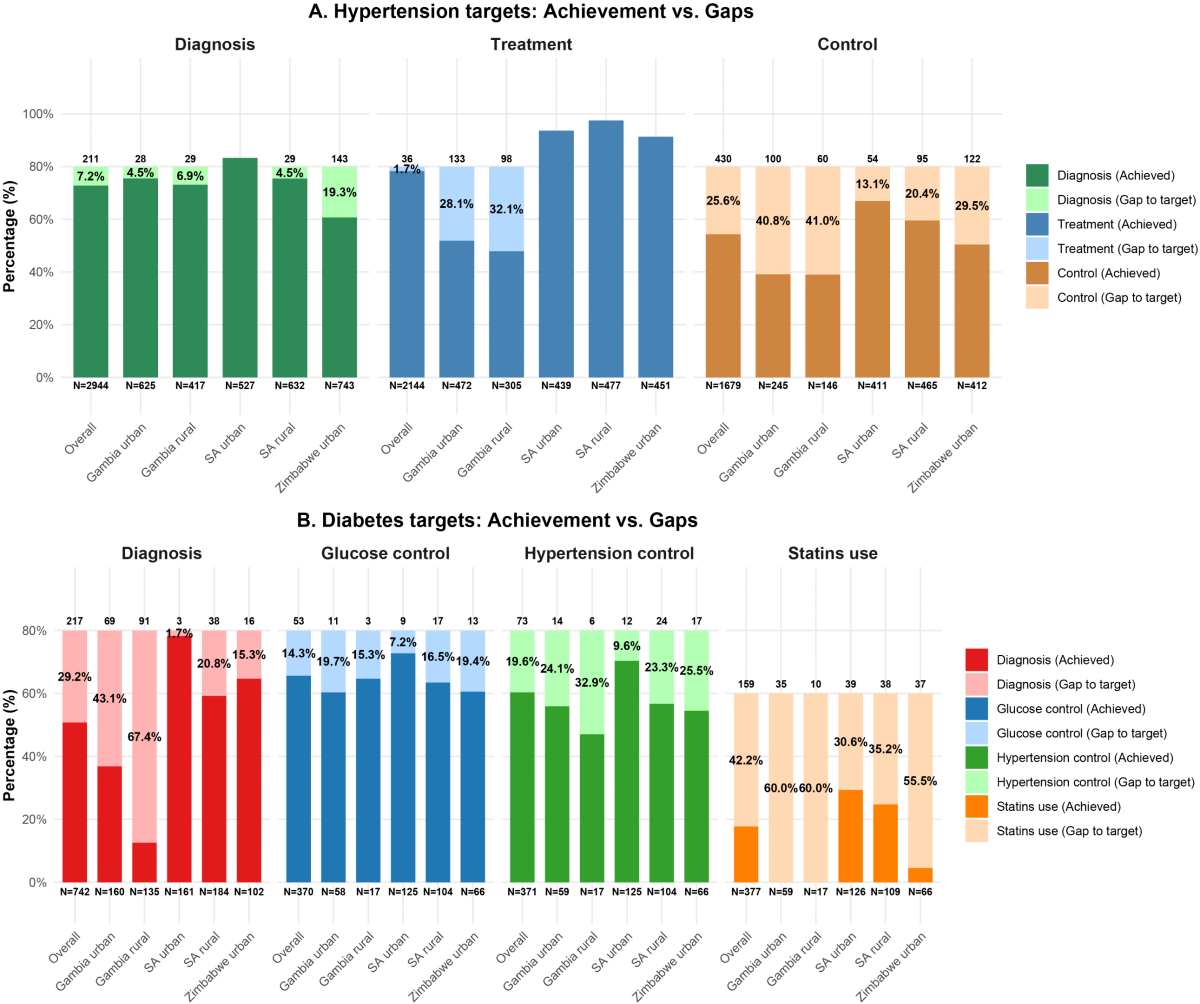
Achievement and gaps of the World Hypertension League targets for Africa (A) [80-80-80% in diagnosis, treatment, and control] and the global diabetes targets (B) [80-80-80-60% in diagnosis, glucose control, hypertension control, and statin use] across the five study sites.

Overall, the achievement of the 80-80-80% was: 72.8% (2144/2944) for diagnosis (gap, 7.2%), 78.3% (1679/2144) for treatment (gap, 1.7%), and 54.4% (913/1679) for control (gap, 25.6%). Achievement gaps were highest in urban Zimbabwe for diagnosis (19.3%), and rural The Gambia for both treatment (32.1%) and control (41.1%) (Fig 4). Targets for diagnosis were exceeded in urban South Africa, and for treatment in rural and urban South Africa and urban Zimbabwe (Fig 4).

### Global diabetes targets

Overall, achievement of the 80-80-80-60% was 50.8% (377/742) for diagnosis (gap, 29.2%), 65.7% (243/370) for glucose control (gap, 14.3%), 60.4% (224/371) for blood pressure control (gap, 19.6%), and 17.8% (67/377) for statin use (gap, 42.2%). Gaps in achievement of diagnosis ranged from 1.7% in urban South Africa to 67.4% in rural The Gambia (Fig 4). Glucose control achievement gaps ranged from 7.2% in urban South Africa to 19.7% in urban The Gambia and 19.4% in urban Zimbabwe. Hypertension control was best in urban South Africa (a gap of only 9.6%) and worst in rural The Gambia (32.9%). None of the 76 participants with diagnosed diabetes in The Gambia were on statins (Fig 4). Overall, only 48 people with diabetes (6.5%) were on aspirin: none in rural The Gambia, six (3.8%) in urban The Gambia, 19 (10.3%) in rural South Africa, 18 (11.2%) in urban South Africa, and 5 (4.9%) in urban Zimbabwe.

### Health-related quality of life

Overall, the mean±SD HRQoL utility score was 0.829 ± 0.107, and the MID in HRQoL (i.e., 0.5*SD) was 0.054.

#### Association between hypertension care cascade and quality of life.

**Fig 5:**
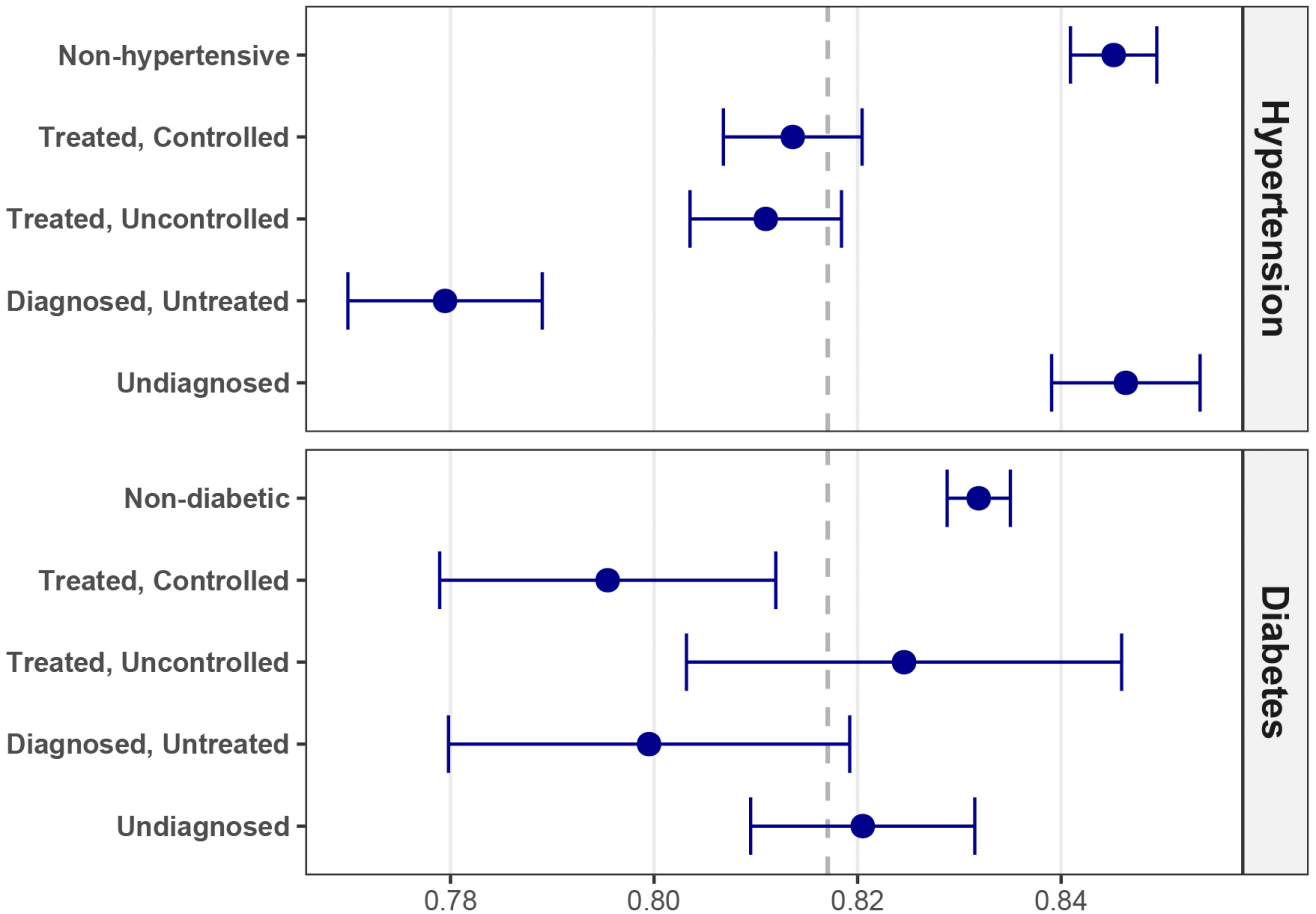
Health-related quality of life utility score (with 95% confidence intervals) in different care cascade stages for hypertension and diabetes in all three countries. The broken line represents the median utility score.

Overall, compared to being nonhypertensive, having diagnosed but untreated hypertension achieved the MID in HRQoL, −0.07 (95% CI [−0.05, −0.08]; *p* < 0.001) (Fig 5, Table K in S1 Appendix). Similarly, MID was observed in The Gambia, −0.07 (95% CI [−0.05, −0.08]; *p* < 0.001) and to a lesser extent South Africa, −0.05 (95% CI [−0.01, −0.09]; *p* = 0.022) (Fig B, Table L in S1 Appendix). Compared to being nonhypertensive, having treated yet uncontrolled hypertension in The Gambia was associated with lower HRQoL, −0.05 (95% CI [−0.03, −0.06]; *p* < 0.001), as was having treated and controlled hypertension in Zimbabwe, −0.06 (95% CI [−0.04, −0.08]; *p* < 0.001) (Fig B, Table L in S1 Appendix). In contrast, being undiagnosed was broadly similar in HRQoL with being nonhypertensive (Fig 5).

#### Association between diabetes care cascade and quality of life.

Overall, compared to those without diabetes, being undiagnosed, untreated, or having uncontrolled or controlled diabetes was not associated with a MID in HRQoL. However, notable associations were observed in individual countries. Compared to being nondiabetic, having treated and controlled diabetes in The Gambia and Zimbabwe was associated with lower HRQoL, −0.07 (95% CI [−0.01, −0.13]; *p* = 0.028) and −0.08 (95% CI [−0.03, −0.12]; *p* < 0.001), respectively (Fig B, Table L in S1 Appendix). Similarly, having diagnosed and untreated diabetes in Zimbabwe was associated with lower HRQoL, −0.11 (95% CI [−0.03, −0.19]; *p* = 0.005) (Fig B, Table L in S1 Appendix). Generally similar trends were observed when the HRQoL VAS was used (Fig C in S1 Appendix).Sensitivity analyses

Trends and magnitude of differences in HRQoL were similar when the Ghanian value set was used (Fig D and Table M in S1 Appendix).

#### Association between the number of medicines, complications, and disease control.

There was no association between the number of medicines taken and control of diabetes or hypertension. Overall, the proportion with ≥1 diabetes complication ranged from 5.5% (*n* = 20/365) in the undiagnosed group to 29.2% (*n* = 47/161) in the treated and controlled group (Table N in S1 Appendix).

**Fig 6:**
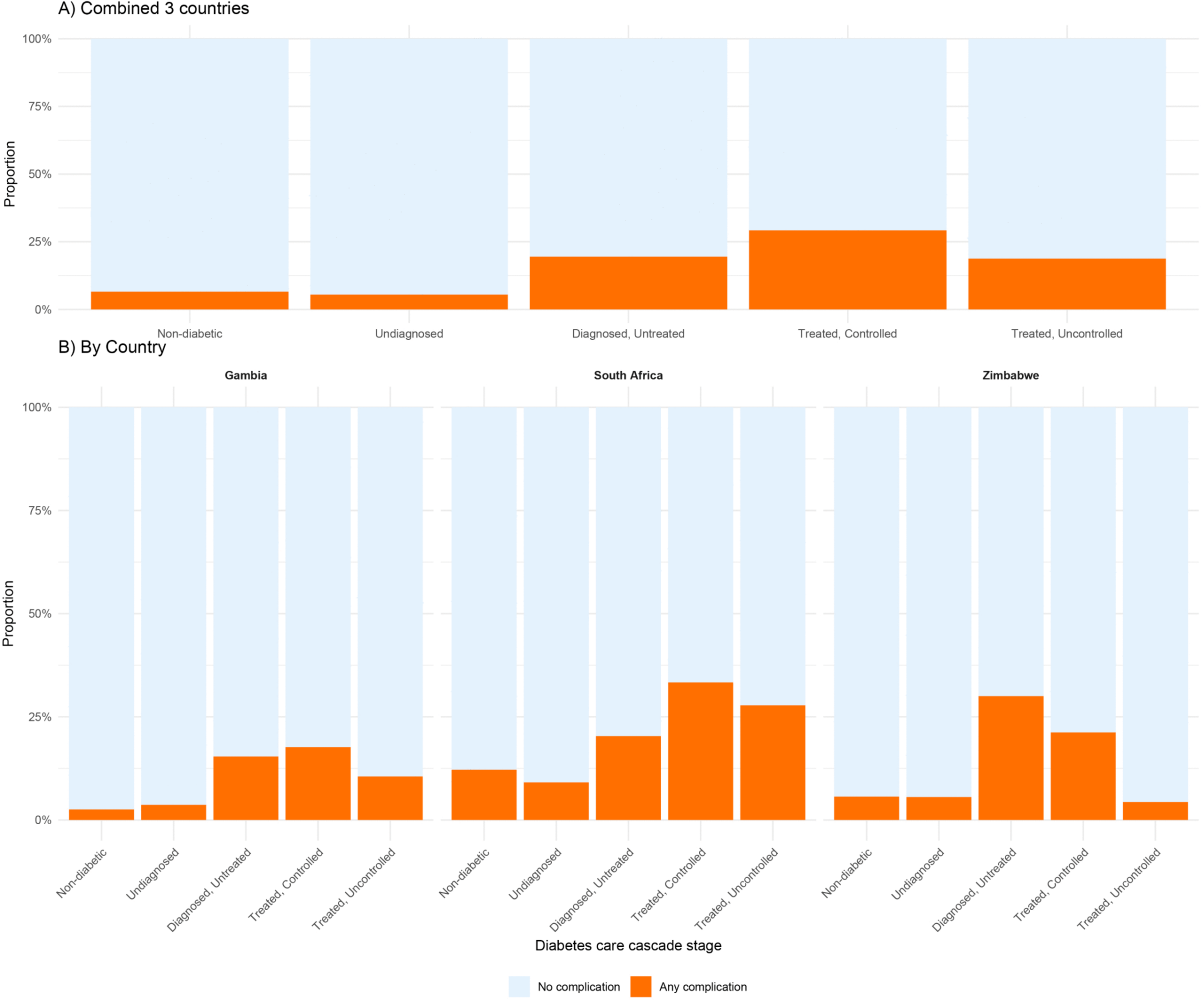
Proportion with no complication or any complication in different diabetes care cascade stages in all three countries (Panel A) and across the three countries (Panel B).

The most common complication was cardiovascular disease (6.9%) (Table P in S1 Appendix). Having ≥1 complication was more common in: 1) those with diagnosed diabetes, irrespective of treatment, and 2) in South Africa and Zimbabwe, compared to The Gambia (Fig 6, Table Q in S1 Appendix). Having ≥1 complication was associated with a lower HRQoL (−0.04 [−0.03, −0.05], *p* < 0.001).

## Discussion

This analysis reports a high prevalence of hypertension and diabetes in both rural and urban settings among adults in The Gambia, South Africa, and Zimbabwe, with prevalence increasing with age. Independent of age, sex, educational attainment, and household wealth, both diseases were associated with overweight and obesity, whilst diabetes was also associated with high central obesity. Only 31% and 22% had treated and controlled hypertension and diabetes, respectively, with only 15% with a diabetes-hypertension comorbidity having both diseases treated and controlled. Undiagnosed disease contributed most to failure of the care cascade for both diseases; this was particularly evident in younger Gambians. Achievement of the 2022 World Hypertension League’s targets for Africa was low in Zimbabwe for diagnosis and in The Gambia for treatment and control. Furthermore, achievement of 2022 global targets for diabetes diagnosis, glucose control, hypertension control, and statin use were all low in The Gambia and Zimbabwe, and somewhat better in South Africa. Having diagnosed but untreated hypertension was associated with lower quality of life, as was treated and controlled diabetes in The Gambia and Zimbabwe, potentially due to having more diabetes complications.

The overall hypertension prevalence of 56% in the current study (of adults ≥40 years) is similar to the pooled prevalence of 57% based on 34 studies in African adults aged ≥50 years, which included two studies from South Africa [10]. Similarly, our study’s overall diabetes prevalence of 14% is consistent with the pooled prevalence (14%) in African adults aged ≥55 years, based on 49 studies, including two from South Africa and one from Zimbabwe [8]. Additionally, the association between excess weight (overweight and obesity) and hypertension and diabetes is consistent with other African [20,21] and global literature [43,44]. Excess weight is a modifiable but complex risk factor necessitating individual-level (e.g., increasing awareness to inform behaviour change), social-level (e.g., changing social norms related to body weight), and environmental-level (e.g., access to healthy foods) interventions [45]. Evaluated obesity interventions in Africa have primarily focussed on individual-level interventions and have been assessed in younger adults (mean age of 25 years) [46]; therefore, there is a need to evaluate other intervention types and include older adults. Meanwhile, among older adults such as those included in the current study, the rollout of WHO Integrated Care for Older People (ICOPE) framework, a set of evidence-based, community-level interventions for various intrinsic capacity impairments, including nutrition [47] may provide the much-needed individual-level obesity prevention and management interventions in this age group.

There is a low rate of treatment and control of hypertension and diabetes, 31% and 22% respectively, with treatment and control being more common in older adults, women, and those living in South Africa, where access to healthcare is better. The proportion with controlled hypertension in this current study is higher than previously reported, at 13% in women and 9% in men, in a younger population (age 30–79 years), across SSA countries, including data from The Gambia, South Africa, and Zimbabwe [5]. The proportion with controlled diabetes in the current study is similar to previous estimates of 22% based on 233 studies globally [48], and 23% in 28 low- and middle-income countries, including South Africa [13], but lower than an analysis in 40–60-year-olds in Kenya, Burkina Faso, Ghana, and South Africa, 38% [14]. This current study’s findings that treatment and control are more common in older adults and women concur with a global systematic review [48]. Findings may be explained by older adults’ increased perceived risk (hence better health-seeking behaviour) and women’s greater contact with the healthcare system through their life course, or a healthy survivor bias.

To standardise diabetes care, WHO member states recently adopted the global diabetes targets (80-80-80-60% in diagnosis, glucose control, hypertension control, and statin use), which aim to screen, treat, and manage diabetes (including a target on hypertension control) for all adults aged ≥40 years. This study offers an initial audit of the global diabetes targets in three African countries. Compared to recent audits in other parts of the world, the gaps in achieving these targets were higher for diabetes diagnosis (29% versus 7% in Iran) and statin use (42% versus 4% in the USA and 10% in Iran) [49,50]. However, the gaps in achievement were lower for glucose control (14% versus 30% in the USA and 43% in Iran) [49,50]. The gap in achieving hypertension control was higher than in the USA (20% versus 10%) and lower than in Iran (20% versus 25%) [49,50]. However, it should be noted that glucose control in the current study was defined using a single fasting or random blood glucose test rather than glycated haemoglobin (HbA1c), as used in the Iran and USA studies [48,49], which may not be applicable in African populations [46]. Furthermore, lower achievement in diabetes diagnosis means that the achievements of other targets are not representative of most people with diabetes. Moreover, achievement of the global diabetes targets varied across study sites and countries, for example, The Gambia and Zimbabwe were less likely to achieve these than South Africa. Whilst in South Africa, urban dwellers had better achievement than rural dwellers. Economic differences could explain these differences: firstly, at a country-level, South Africa is an upper-middle income country [18], secondly, at the individual-level, low household wealth was more common in rural than urban South Africa. Furthermore, differences could reflect differences in treatment guidelines. For instance, statins were not on The Gambia’s 2019 essential medicines list [51], potentially explaining the absence of treatment in people with diabetes. Overall, use of most of the antihypertensive medications (11/13 unique medications, 84.6%) and half of the diabetes medications (3/6 unique medications, 50.0%) listed in the 2025 WHO Model Lists of Essential Medicines [52] was reported. Similar to the successes in achieving 95-95-95 HIV targets across countries with wide-ranging economies [53], achievement of diabetes targets will require specific funding, stakeholder engagement, multicomponent interventions, and health systems realignment to prioritise screening, treatment, and control of diabetes and hypertension [54].

Finally, this study contributes to the scant evidence on associations between hypertension and diabetes care cascade achievement and quality of life. These relationships are complex, as they are influenced by symptom severity and count, incidence and control of complications, optimisation and duration of treatment, among other factors. Previous single-country studies have been conflicting; a study in Congo found that adherence to hypertension medication compared to nonadherence was associated with a better quality of life [55], whereas a Nigerian study found that being on antihypertensives was associated with a lower quality of life compared to being untreated, whilst hypertension itself had no effect [56]. A systematic review on quality of life in people living with diabetes in SSA found that lower quality of life was predicted by drug combinations and complications, and adherence was associated with better quality of life [22,23]. Taken together, these findings have important policy, practice, and research implications. First, screening and diagnosis should be promptly followed by treatment to limit symptom severity and complications, preserving quality of life. Second, those diagnosed should be screened and treated for complications. Third, while monitoring progress towards targets is important, their achievement does not always translate to patient-important outcomes, such as quality of life [57]. Therefore, monitoring of patient-important outcomes, along with progress on diagnosis, treatment, and control, is needed.

The strengths of this study include a large population-based sample, rural-urban comparisons, the inclusion of middle-aged and older adults, an audit of African hypertension and global diabetes targets, and examination of associations between care cascade stages and quality of life in three diverse African countries. However, findings should be interpreted in the context of limitations. Firstly, the choice not to undertake a causal inference analysis precludes any causal claims about the observed associations. Secondly, for logistical reasons, diabetes and hypertension were diagnosed using single measures; reassuringly, those diagnosed with diabetes had a higher ADRS: a validated score to identify undiagnosed diabetes [29]. The ADRS was not computed in The Gambia; however, the development of ADRS used data from Senegal [29], whose population is comparable to that of The Gambia. Additionally, a single elevated random blood glucose measurement is considered a robust risk factor for undiagnosed diabetes [58]. Thirdly, treatment definitions did not include lifestyle modification advice, as these data were not available. Fourthly, diabetes control was defined using random and fasting blood glucose rather than HbA1c. However, evidence from rural Zambia suggests that random blood glucose levels correlate with HbA1c in individuals with noninsulin-treated diabetes, providing a potentially cheaper alternative for monitoring glucose control [59]. Furthermore, valid cut-offs for HbA1c remain unknown for African populations [46]. Additionally, only three diabetes complications were included in the analysis, with neuropathy not specifically assessed but identified from reported medication indications. Furthermore, while type 2 diabetes was assumed, some, most likely very few, participants may have had type 1 diabetes, although their age makes this less likely in this challenging context. Only 10 participants self-reported use of insulin alone, with five younger than 65 years. Globally, the proportions of type 1 diabetes cases in the 40–64 and ≥65 years age groups are 43% and 16%, respectively, with Africa contributing very low proportions of global cases (8%) compared to other regions, such as Asia (31%) [60]. Moreover, the use of Zimbabwean and Ghanaian (for sensitivity analyses) HRQoL value sets was not optimal for The Gambia and South Africa. Furthermore, the lack of representation from Eastern and Central Africa regions limits the generalisability of the findings to the continent. Also, the use of ‘overall’ in the current study is not reflective of a particular population but the combined sample from all study sites. Whilst analyses were pre-planned, the statistical analysis plan was not published. Finally, whilst data were collected over 2 years, healthcare delivery for hypertension and diabetes did not materially change over this period, so the effects of timing within and between countries would have been minimal. Despite these limitations, this study reports a high prevalence of hypertension and diabetes in middle-aged and older adults in three African countries. Obesity interventions could be an important strategy for preventing both diseases. There is a high unmet need in the treatment of both diseases and the achievement of the global diabetes targets, particularly in men, and in The Gambia and Zimbabwe. This necessitates urgent realignment of health systems to prioritise awareness raising, screening of all adults ≥40 years, prompt treatment, and optimal management of hypertension and diabetes, including related complications. Monitoring of patient-important outcomes, such as secondary complications, is needed along with auditing the progress towards achievement of diabetes and hypertension control.

## Supporting information

S1 Appendix Supplementary Tables A–Q and Figures A–D. **Table A:** Prevalence of hypertension and diabetes by study site and sex. **Fig A:** Prevalence of hypertension and diabetes by study site and age group. **Table B:** Sensitivity analysis: associations between diabetes and hypertension and sociodemographic, household, and lifestyle factors without binarisation. **Table C:** Descriptives of the care cascade (diagnosed, treated, controlled) by site. **Table D:** Care cascades by site and sex. **Table E:** Medication and classes for antihypertensives. **Table F:** Medication and classes for diabetes medications. **Table G:** Proportions of medication classes in the 3 countries. **Table H:** Health-Related Quality of Life Utility Scores by care cascade stage. **Table J:** Health-Related Quality of Life Utility Scores by care cascade stage in the 3 countries. **Table K:** Differences in utility score between nonhypertensive or nondiabetic with care cascade stages. **Table L:** Differences in utility score between nonhypertensive or nondiabetic and care cascade stages in the 3 countries. **Fig B:** Health-related quality of life utility score (with 95% confidence intervals) in different care cascade stages for hypertension and diabetes across the three countries. The broken line represents the median utility score. **Fig C:** Health-related quality of life visual analogue scale score (with 95% confidence intervals) in different care cascade stages for hypertension and diabetes across the three countries. The broken line represents the median score. **Table M:** Sensitivity analyses - differences in utility score (Ghanian value set) between nonhypertensive or nondiabetic and care cascade stages in the 3 countries. **Fig D:** Sensitivity analyses using the Ghanian value set. **Table N:** Overall proportion (95% confidence intervals) with ≥1 diabetes complications. **Table P:** Prevalence of diabetes complications overall and in the three countries. **Table Q:** Proportion (95% confidence intervals) with ≥1 diabetes complications across the three countries.
(DOCX)

S1 Checklist STROBE Statement—Checklist of items that should be included in reports of *cross-sectional studies* available from https://doi.org/10.1136/bmj.39335.541782.AD and www.strobe-statement.org.
(DOC)

S1 Text Inclusivity in the global research questionnaire. (DOCX)

## Abbreviations

ACE: angiotensin-converting-enzyme
ADRS: visual analogue scale
BMI: body mass index
BREC: Biomedical Research Ethics Committee
CI: confidence intervals
DBP: diastolic blood pressure
GLMs: generalised linear models
HbA1c: glycated haemoglobin
ICOPE: Integrated Care for Older People
IDF: International Diabetes Federation
MET: Metabolic equivalent task
MID: minimally important difference
NCDs: noncommunicable diseases
PROMs: patient-reported outcome measures
SA: South Africa
SBP: systolic blood pressure
SD: standard deviation
STEPS: STEPwise approach to NCD risk factor surveillance
VAS: African Diabetes Risk Score
WHR: waist-to-hip ratio.
