# Supplementary material for: Hypertension and diabetes prevalence, associated factors, care cascade, and quality of life in older adults: A cross-sectional population-based study in The Gambia, South Africa, and Zimbabwe
Source: PLoS Med. 2026 Jul 7;23(7):e1004785. doi: 10.1371/journal.pmed.1004785 (PMC13367908; doi:10.1371/journal.pmed.1004785)
Supplement: S1 Appendix — Table A: Prevalence of hypertension and diabetes by study site and sex. Fig A: Prevalence of hypertension and diabetes by study site and age group. Table B: Sensitivity analysis: associations between diabetes and hypertension and sociodemographic, household, and lifestyle factors without binarisation. Table C: Descriptives of the care cascade (diagnosed, treated, controlled) by site. Table D: Care cascades by site and sex. Table E: Medication and classes for antihypertensives. Table F: Medication and classes for diabetes medications. Table G: Proportions of medication classes in the 3 countries. Table H: Health-Related Quality of Life Utility Scores by care cascade stage. Table J: Health-Related Quality of Life Utility Scores by care cascade stage in the 3 countries. Table K: Differences in utility score between nonhypertensive or nondiabetic with care cascade stages. Table L: Differences in utility score between nonhypertensive or nondiabetic and care cascade stages in the 3 countries. Fig B: Health-related quality of life utility score (with 95% confidence intervals) in different care cascade stages for hypertension and diabetes across the three countries. The broken line represents the median utility score. Fig C: Health-related quality of life visual analogue scale score (with 95% confidence intervals) in different care cascade stages for hypertension and diabetes across the three countries. The broken line represents the median score. Table M: Sensitivity analyses - differences in utility score (Ghanian value set) between nonhypertensive or nondiabetic and care cascade stages in the 3 countries. Fig D: Sensitivity analyses using the Ghanian value set. Table N: Overall proportion (95% confidence intervals) with ≥1 diabetes complications. Table P: Prevalence of diabetes complications overall and in the three countries. Table Q: Proportion (95% confidence intervals) with ≥1 diabetes complications across the three countries. (DOCX) [file pmed.1004785.s001.docx]

S1 Appendix

Table of Contents

[Table A: Prevalence of hypertension and diabetes by study site and sex 3](#_Toc229747602)

[Figure A: Prevalence of hypertension and diabetes by study site and age group 5](#_Toc229747603)

[Table B: Sensitivity analysis: associations between diabetes and hypertension and sociodemographic, household and lifestyle factors without binarisation 6](#_Toc229747604)

[Table C: Descriptives of the care cascade (diagnosed, treated, controlled) by site 9](#_Toc229747605)

[Table D: Care cascades by site, and sex 10](#_Toc229747606)

[Table E: Medication and classes for antihypertensives 12](#_Toc229747607)

[Table F: Medication and classes for diabetes medications 14](#_Toc229747608)

[Table G: Proportions of medication classes in the 3 countries 14](#_Toc229747609)

[Table H: Health-Related Quality of Life Utility Scores by care cascade stage 16](#_Toc229747610)

[Table J: Health-Related Quality of Life Utility Scores by care cascade stage in the 3 countries 17](#_Toc229747611)

[Table K: Differences in utility score between non-hypertensive or non-diabetic with care cascade stages 18](#_Toc229747612)

[Table L: Differences in utility score between non-hypertensive or non-diabetic and care cascade stages in the 3 countries 19](#_Toc229747613)

[Figure B: Health-related quality of life utility score (with 95% confidence intervals) in different care cascade stages for hypertension and diabetes across the three countries. The broken line represents the median utility score. 20](#_Toc229747614)

[Figure C: Health-related quality of life visual analogue scale score (with 95% confidence intervals) in different care cascade stages for hypertension and diabetes across the three countries. The broken line represents the median score 21](#_Toc229747615)

[Table M: Sensitivity analyses - differences in utility score (Ghanian value set) between non-hypertensive or non-diabetic and care cascade stages in the 3 countries 22](#_Toc229747616)

[Figure D: Sensitivity analyses using the Ghanian value set 24](#_Toc229747617)

[Table N: Overall proportion (95% confidence intervals) with ≥1 diabetes complications 25](#_Toc229747618)

[Table P: Prevalence of diabetes complications overall and in the three countries 25](#_Toc229747619)

[Table Q: Proportion (95% confidence intervals) with ≥1 diabetes complications across the three countries 25](#_Toc229747620)

# Table A: Prevalence of hypertension and diabetes by study site and sex

| **Condition** | **Site** | **Male_n** | **Female_n** | **Total_n** | **Male_cases** | **Female_cases** | **Total_cases** | **Male_% prevalence_ci** | **Female_%prevalence_ci** | **Total_%prevalence_ci** |
| --- | --- | --- | --- | --- | --- | --- | --- | --- | --- | --- |
| **Hypertension** | Overall | 2,473 | 2,823 | 5,296 | 1,265 | 1,679 | 2,944 | 51.2 (49.2–53.1) | 59.5 (57.6–61.3) | 55.6 (54.2–56.9) |
|  | Gambia urban | 547 | 671 | 1,218 | 264 | 361 | 625 | 48.3 (44.0–52.5) | 53.8 (49.9–57.6) | 51.3 (48.5–54.2) |
|  | Gambia rural | 505 | 547 | 1,052 | 192 | 225 | 417 | 38.0 (33.8–42.4) | 41.1 (37.0–45.4) | 39.6 (36.7–42.7) |
|  | SA urban | 448 | 520 | 968 | 209 | 318 | 527 | 46.7 (42.0–51.4) | 61.2 (56.8–65.4) | 54.4 (51.2–57.6) |
|  | SA rural | 435 | 513 | 948 | 263 | 369 | 632 | 60.5 (55.7–65.1) | 71.9 (67.8–75.8) | 66.7 (63.6–69.7) |
|  | Zimbabwe urban | 538 | 572 | 1,110 | 337 | 406 | 743 | 62.6 (58.4–66.7) | 71.0 (67.1–74.7) | 66.9 (64.1–69.7) |
| **Diabetes** | Overall | 2,473 | 2,823 | 5,296 | 289 | 453 | 742 | 11.7 (10.4–13.0) | 16.0 (14.7–17.5) | 14.0 (13.1–15.0) |
|  | Gambia urban | 547 | 671 | 1,218 | 64 | 96 | 160 | 11.7 (9.1–14.7) | 14.3 (11.7–17.2) | 13.1 (11.3–15.2) |
|  | Gambia rural | 505 | 547 | 1,052 | 60 | 75 | 135 | 11.9 (9.2–15.0) | 13.7 (10.9–16.9) | 12.8 (10.9–15.0) |
|  | SA urban | 448 | 520 | 968 | 56 | 105 | 161 | 12.5 (9.6–15.9) | 20.2 (16.8–23.9) | 16.6 (14.3–19.1) |
|  | SA rural | 435 | 513 | 948 | 74 | 110 | 184 | 17.0 (13.6–20.9) | 21.4 (18.0–25.3) | 19.4 (16.9–22.1) |
|  | Zimbabwe urban | 538 | 572 | 1,110 | 35 | 67 | 102 | 6.5 (4.6–8.9) | 11.7 (9.2–14.6) | 9.2 (7.6–11.0) |
| **Diabetes-Hypertension Comorbidity** | Overall | 2,473 | 2,823 | 5,296 | 204 | 351 | 555 | 8.2 (7.2–9.4) | 12.4 (11.2–13.7) | 10.5 (9.7–11.3) |
|  | Gambia urban | 547 | 671 | 1,218 | 39 | 69 | 108 | 7.1 (5.1–9.6) | 10.3 (8.1–12.8) | 8.9 (7.3–10.6) |
|  | Gambia rural | 505 | 547 | 1,052 | 30 | 35 | 65 | 5.9 (4.0–8.4) | 6.4 (4.5–8.8) | 6.2 (4.8–7.8) |
|  | SA urban | 448 | 520 | 968 | 47 | 93 | 140 | 10.5 (7.8–13.7) | 17.9 (14.7–21.5) | 14.5 (12.3–16.8) |
|  | SA rural | 435 | 513 | 948 | 56 | 94 | 150 | 12.9 (9.9–16.4) | 18.3 (15.1–21.9) | 15.8 (13.6–18.3) |
|  | Zimbabwe urban | 538 | 572 | 1,110 | 32 | 60 | 92 | 5.9 (4.1–8.3) | 10.5 (8.1–13.3) | 8.3 (6.7–10.1) |
| **prevalence_ci – 95 % Confidence Intervals**  **SA – South Africa** | | | | | | | | | | |

# Figure A: Prevalence of hypertension and diabetes by study site and age group


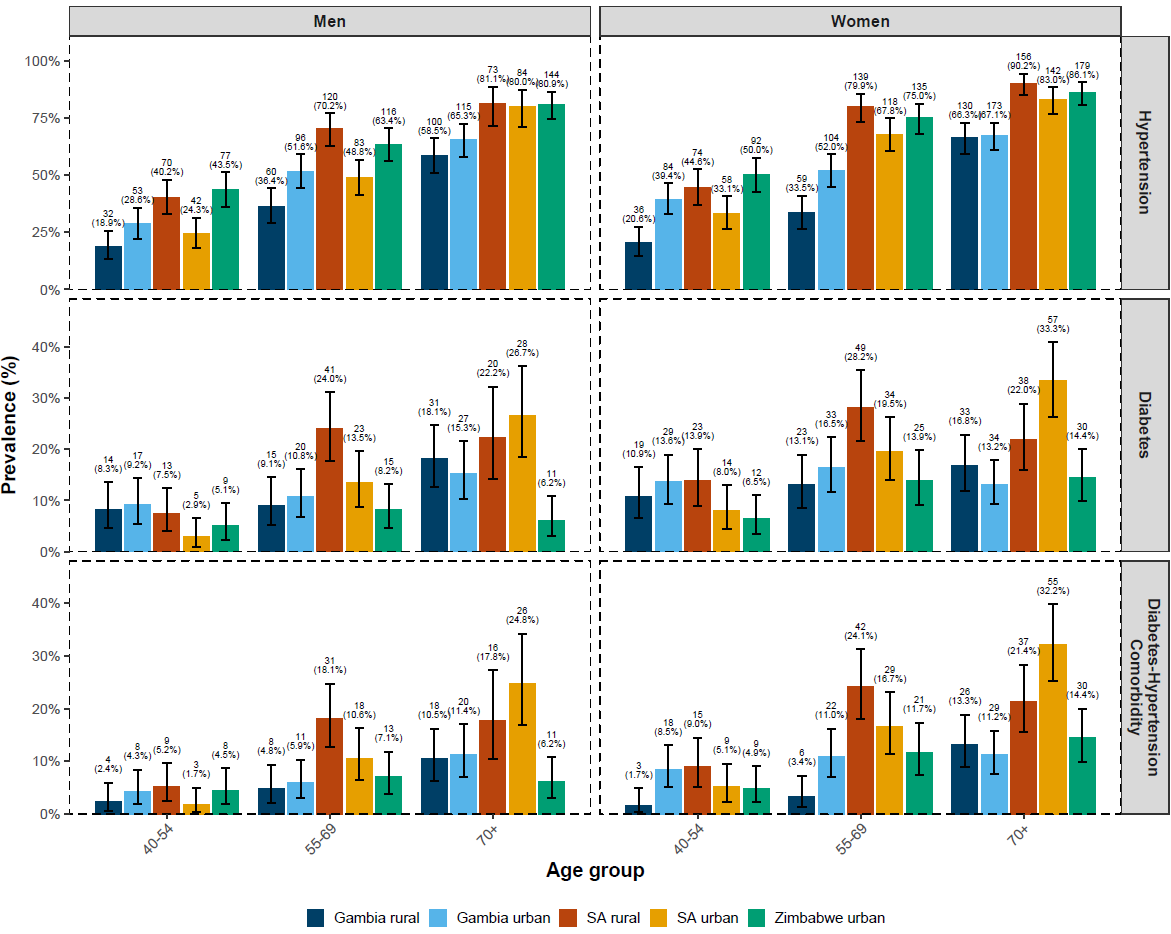


# Table B: Sensitivity analysis: associations between diabetes and hypertension and sociodemographic, household and lifestyle factors without binarisation

| **Variable** | **Category** | **Hypertension** | | | | | | **Diabetes** | | | | | |
| --- | --- | --- | --- | --- | --- | --- | --- | --- | --- | --- | --- | --- | --- |
|  |  | **Model 1** | | **Model 2** | | **Model 3** | | **Model 1** | | **Model 2** | | **Model 3** | |
|  |  | **OR (95% CI)** | **p** | **OR (95% CI)** | **p** | **OR (95% CI)** | **p** | **OR (95% CI)** | **p** | **OR (95% CI)** | **p** | **OR (95% CI)** | **p** |
| **Education** | Primary (ref) | 1.00 (ref) | – | 1.00 (ref) | – | 1.00 (ref) | – | 1.00 (ref) | – | 1.00 (ref) | – | 1.00 (ref) | – |
|  | Secondary | 0.53 (0.45–0.62) | <0.001 | 0.93 (0.78–1.11) | 0.438 | 0.92 (0.78–1.10) | 0.378 | 0.63 (0.51–0.78) | <0.001 | 0.82 (0.65–1.02) | 0.074 | 0.81 (0.64–1.01) | 0.057 |
|  | Training college | 0.77 (0.54–1.08) | 0.133 | 1.19 (0.82–1.72) | 0.353 | 1.16 (0.80–1.68) | 0.421 | 0.63 (0.38–1.06) | 0.084 | 0.79 (0.47–1.34) | 0.380 | 0.76 (0.45–1.29) | 0.309 |
|  | University | 0.45 (0.25–0.81) | 0.008 | 0.79 (0.42–1.47) | 0.457 | 0.75 (0.40–1.41) | 0.373 | 0.53 (0.21–1.38) | 0.196 | 0.75 (0.29–1.98) | 0.568 | 0.69 (0.26–1.82) | 0.457 |
|  | None | 1.75 (1.39–2.20) | <0.001 | 1.06 (0.83–1.36) | 0.637 | 1.08 (0.84–1.38) | 0.547 | 0.94 (0.69–1.28) | 0.697 | 0.74 (0.54–1.01) | 0.061 | 0.76 (0.56–1.05) | 0.096 |
|  | Unknown | 1.22 (0.62–2.37) | 0.563 | 0.63 (0.31–1.26) | 0.189 | 0.64 (0.32–1.28) | 0.204 | 1.53 (0.67–3.51) | 0.318 | 1.12 (0.48–2.61) | 0.789 | 1.17 (0.50–2.72) | 0.714 |
|  | Dara school | 1.24 (0.99–1.55) | 0.067 | 0.95 (0.74–1.21) | 0.651 | 0.96 (0.75–1.23) | 0.763 | 0.98 (0.71–1.36) | 0.925 | 0.90 (0.65–1.25) | 0.537 | 0.93 (0.67–1.30) | 0.667 |
| **Food insecurity score (number of affirmative responses)** | 0 – none (ref) | 1.00 (ref) | – | 1.00 (ref) | – | 1.00 (ref) | – | 1.00 (ref) | – | 1.00 (ref) | – | 1.00 (ref) | – |
|  | 1 | 1.08 (0.87–1.35) | 0.483 | 1.02 (0.80–1.29) | 0.886 | 1.03 (0.81–1.30) | 0.822 | 1.28 (0.96–1.70) | 0.088 | 1.27 (0.95–1.69) | 0.102 | 1.29 (0.96–1.72) | 0.088 |
|  | 2 | 0.78 (0.59–1.02) | 0.065 | 0.82 (0.61–1.09) | 0.165 | 0.80 (0.60–1.07) | 0.138 | 1.18 (0.80–1.72) | 0.403 | 1.22 (0.83–1.80) | 0.302 | 1.22 (0.83–1.79) | 0.318 |
|  | 3 | 0.85 (0.65–1.11) | 0.229 | 0.89 (0.67–1.19) | 0.448 | 0.88 (0.66–1.18) | 0.394 | 0.85 (0.56–1.29) | 0.434 | 0.87 (0.57–1.33) | 0.523 | 0.90 (0.58–1.37) | 0.611 |
|  | 4 | 0.94 (0.79–1.12) | 0.498 | 1.02 (0.85–1.22) | 0.862 | 1.03 (0.85–1.23) | 0.782 | 0.80 (0.62–1.04) | 0.096 | 0.83 (0.64–1.08) | 0.161 | 0.85 (0.65–1.10) | 0.208 |
|  | 5 – all | 0.92 (0.75–1.12) | 0.389 | 0.99 (0.80–1.22) | 0.900 | 1.02 (0.82–1.26) | 0.870 | 1.01 (0.77–1.32) | 0.952 | 1.06 (0.80–1.39) | 0.688 | 1.07 (0.81–1.41) | 0.628 |
| **Marital status** | Never married (ref) | 1.00 (ref) | – | 1.00 (ref) | – | 1.00 (ref) | – | 1.00 (ref) | – | 1.00 (ref) | – | 1.00 (ref) | – |
|  | Currently married | 2.15 (1.76–2.61) | <0.001 | 1.41 (1.15–1.74) | 0.001 | 1.40 (1.13–1.73) | 0.002 | 2.58 (1.97–3.38) | <0.001 | 2.33 (1.77–3.08) | <0.001 | 2.25 (1.70–2.97) | <0.001 |
|  | Separated | 1.70 (1.02–2.83) | 0.041 | 1.36 (0.80–2.31) | 0.256 | 1.36 (0.80–2.31) | 0.260 | 3.66 (1.79–7.48) | <0.001 | 3.50 (1.71–7.18) | <0.001 | 3.41 (1.66–7.01) | <0.001 |
|  | Divorced | 2.21 (1.58–3.09) | <0.001 | 1.45 (1.02–2.07) | 0.038 | 1.45 (1.02–2.06) | 0.041 | 2.16 (1.32–3.52) | 0.002 | 1.80 (1.10–2.96) | 0.020 | 1.75 (1.06–2.88) | 0.029 |
|  | Widowed | 5.11 (4.08–6.41) | <0.001 | 1.62 (1.26–2.09) | <0.001 | 1.60 (1.24–2.06) | <0.001 | 3.11 (2.32–4.15) | <0.001 | 1.93 (1.40–2.66) | <0.001 | 1.91 (1.39–2.64) | <0.001 |
|  | Cohabitating | 0.43 (0.23–0.79) | 0.007 | 0.51 (0.27–0.97) | 0.040 | 0.52 (0.27–0.98) | 0.042 | 0.79 (0.28–2.26) | 0.660 | 0.91 (0.32–2.61) | 0.860 | 0.92 (0.32–2.65) | 0.882 |
|  | Decline to answer | 1.30 (0.08–21.42) | 0.852 | 1.59 (0.08–30.29) | 0.758 | 1.76 (0.09–35.33) | 0.710 | 12.17 (0.67–222.42) | 0.092 | 13.61 (0.71–260.79) | 0.083 | 13.65 (0.67–276.42) | 0.089 |
| **Continuous variables** | | | | | | | | | | | | | |
| **Age (years)** | | 1.07 (1.06–1.07) | <0.001 | 1.07 (1.06–1.07) | <0.001 | 1.07 (1.06–1.07) | <0.001 | 1.03 (1.02–1.03) | <0.001 | 1.02 (1.02–1.03) | <0.001 | 1.02 (1.02–1.03) | <0.001 |
| **BMI (kg/m²)** | | 1.05 (1.05–1.06) | <0.001 | 1.06 (1.05–1.08) | <0.001 | 1.06 (1.05–1.08) | <0.001 | 1.05 (1.03–1.06) | <0.001 | 1.04 (1.03–1.06) | <0.001 | 1.04 (1.03–1.06) | <0.001 |
| Physical activity (per 1000 MET-min/week) | | 0.96 (0.95–0.97) | <0.001 | 0.99 (0.97–1.00) | 0.042 | 0.99 (0.97–1.00) | 0.056 | 0.98 (0.96–1.00) | 0.046 | 1.00 (0.98–1.01) | 0.632 | 1.00 (0.98–1.02) | 0.701 |
| **Wealth index** | | 1.21 (1.01–1.45) | 0.043 | 1.15 (0.95–1.39) | 0.162 | 1.18 (0.97–1.44) | 0.099 | 1.46 (1.11–1.93) | 0.007 | 1.40 (1.06–1.86) | 0.018 | 1.27 (0.72–2.25) | 0.406 |
| Model 1: Adjusted for site. Model 2: Adjusted for site + sex + age. Model 3: Adjusted for site + sex + age + wealth index + education.  *P-values were derived from Wald z-tests.* | | | | | | | | | | | | | |

# Table C: Descriptives of the care cascade (diagnosed, treated, controlled) by site

| **Characteristic** | **Overall^1^** | **Gambia rural^1^** | **Gambia urban^1^** | **SA rural^1^** | **SA urban^1^** | **Zimbabwe urban^1^** |
| --- | --- | --- | --- | --- | --- | --- |
| **Hypertension Care Cascade** | | | | | | |
| Treated, Controlled | 913/2,944 (31.0%) | 57/417 (13.7%) | 96/625 (15.4%) | 277/632 (43.8%) | 275/527 (52.2%) | 208/743 (28.0%) |
| Treated, Uncontrolled | 766/2,944 (26.0%) | 89/417 (21.3%) | 149/625 (23.8%) | 188/632 (29.7%) | 136/527 (25.8%) | 204/743 (27.5%) |
| Diagnosed, Untreated | 465/2,944 (15.8%) | 159/417 (38.1%) | 227/625 (36.3%) | 12/632 (1.9%) | 28/527 (5.3%) | 39/743 (5.2%) |
| Undiagnosed | 800/2,944 (27.2%) | 112/417 (26.9%) | 153/625 (24.5%) | 155/632 (24.5%) | 88/527 (16.7%) | 292/743 (39.3%) |
| **Diabetes Care Cascade** | | | | | | |
| Treated, Controlled | 161/735 (21.9%) | 8/135 (5.9%) | 9/159 (5.7%) | 48/179 (26.8%) | 63/160 (39.4%) | 33/102 (32.4%) |
| Treated, Uncontrolled | 96/735 (13.1%) | 6/135 (4.4%) | 13/159 (8.2%) | 26/179 (14.5%) | 28/160 (17.5%) | 23/102 (22.5%) |
| Diagnosed, Untreated | 113/735 (15.4%) | 3/135 (2.2%) | 36/159 (22.6%) | 30/179 (16.8%) | 34/160 (21.3%) | 10/102 (9.8%) |
| Undiagnosed | 365/735 (49.7%) | 118/135 (87.4%) | 101/159 (63.5%) | 75/179 (41.9%) | 35/160 (21.9%) | 36/102 (35.3%) |
| **Diabetes-Hypertension Comorbidity Care Cascade** | | | | | | |
| Both controlled | 82/555 (14.8%) | 1/65 (1.5%) | 0/108 (0.0%) | 22/150 (14.7%) | 43/140 (30.7%) | 16/92 (17.4%) |
| One controlled | 191/555 (34.4%) | 15/65 (23.1%) | 24/108 (22.2%) | 55/150 (36.7%) | 59/140 (42.1%) | 38/92 (41.3%) |
| Both treated | 35/555 (6.3%) | 2/65 (3.1%) | 5/108 (4.6%) | 12/150 (8.0%) | 9/140 (6.4%) | 7/92 (7.6%) |
| One treated | 109/555 (19.6%) | 13/65 (20.0%) | 25/108 (23.1%) | 37/150 (24.7%) | 18/140 (12.9%) | 16/92 (17.4%) |
| Both diagnosed | 18/555 (3.2%) | 2/65 (3.1%) | 13/108 (12.0%) | 0/150 (0.0%) | 2/140 (1.4%) | 1/92 (1.1%) |
| One diagnosed | 42/555 (7.6%) | 11/65 (16.9%) | 21/108 (19.4%) | 2/150 (1.3%) | 3/140 (2.1%) | 5/92 (5.4%) |
| Neither diagnosed | 78/555 (14.1%) | 21/65 (32.3%) | 20/108 (18.5%) | 22/150 (14.7%) | 6/140 (4.3%) | 9/92 (9.8%) |
| *^1^n/N (%)* | | | | | | |

# Table D: Care cascades by site, and sex

**Table D1: Hypertension Care Cascade (%)**

| **Category** | **Sex** | **Overall** | **Gambia rural** | **Gambia urban** | **SA rural** | **SA urban** | **Zimbabwe urban** |
| --- | --- | --- | --- | --- | --- | --- | --- |
| Treated, Controlled | Men | 23.2 | 11.5 | 11.4 | 32.7 | 47.8 | 16.6 |
| Treated, Controlled | Women | 36.9 | 15.6 | 18.3 | 51.8 | 55.0 | 37.4 |
| Treated, Controlled | Overall | 31.0 | 13.7 | 15.4 | 43.8 | 52.2 | 28.0 |
| Treated, Uncontrolled | Men | 24.0 | 23.4 | 19.7 | 26.6 | 23.9 | 25.8 |
| Treated, Uncontrolled | Women | 27.5 | 19.6 | 26.9 | 32.0 | 27.0 | 28.8 |
| Treated, Uncontrolled | Overall | 26.0 | 21.3 | 23.8 | 29.7 | 25.8 | 27.5 |
| Diagnosed, Untreated | Men | 15.2 | 33.9 | 34.1 | 3.4 | 5.7 | 4.7 |
| Diagnosed, Untreated | Women | 16.3 | 41.8 | 38.0 | 0.8 | 5.0 | 5.7 |
| Diagnosed, Untreated | Overall | 15.8 | 38.1 | 36.3 | 1.9 | 5.3 | 5.2 |
| Undiagnosed | Men | 37.5 | 31.2 | 34.8 | 37.3 | 22.5 | 52.8 |
| Undiagnosed | Women | 19.4 | 23.1 | 16.9 | 15.4 | 12.9 | 28.1 |
| Undiagnosed | Overall | 27.2 | 26.9 | 24.5 | 24.5 | 16.7 | 39.3 |
| Total N | Men | 1,265 | 192 | 264 | 263 | 209 | 337 |
| Total N | Women | 1,679 | 225 | 361 | 369 | 318 | 406 |
| Total N | Overall | 2,944 | 417 | 625 | 632 | 527 | 743 |

**Table D2: Diabetes Care Cascade (%)**

| **Category** | **Sex** | **Overall** | **Gambia rural** | **Gambia urban** | **SA rural** | **SA urban** | **Zimbabwe urban** |
| --- | --- | --- | --- | --- | --- | --- | --- |
| Treated, Controlled | Men | 17.7 | 6.7 | 4.8 | 24.3 | 25.5 | 34.3 |
| Treated, Controlled | Women | 24.6 | 5.3 | 6.2 | 28.4 | 46.7 | 31.3 |
| Treated, Controlled | Overall | 21.9 | 5.9 | 5.7 | 26.8 | 39.4 | 32.4 |
| Treated, Uncontrolled | Men | 12.7 | 8.3 | 6.3 | 11.4 | 20.0 | 22.9 |
| Treated, Uncontrolled | Women | 13.3 | 1.3 | 9.4 | 16.5 | 16.2 | 22.4 |
| Treated, Uncontrolled | Overall | 13.1 | 4.4 | 8.2 | 14.5 | 17.5 | 22.5 |
| Diagnosed, Untreated | Men | 14.8 | 3.3 | 25.4 | 8.6 | 25.5 | 11.4 |
| Diagnosed, Untreated | Women | 15.7 | 1.3 | 20.8 | 22.0 | 19.0 | 9.0 |
| Diagnosed, Untreated | Overall | 15.4 | 2.2 | 22.6 | 16.8 | 21.2 | 9.8 |
| Undiagnosed | Men | 54.8 | 81.7 | 63.5 | 55.7 | 29.1 | 31.4 |
| Undiagnosed | Women | 46.5 | 92.0 | 63.5 | 33.0 | 18.1 | 37.3 |
| Undiagnosed | Overall | 49.7 | 87.4 | 63.5 | 41.9 | 21.9 | 35.3 |
| Total N | Men | 283 | 60 | 63 | 70 | 55 | 35 |
| Total N | Women | 452 | 75 | 96 | 109 | 105 | 67 |
| Total N | Overall | 735 | 135 | 159 | 179 | 160 | 102 |

**Table D3: Hypertension-Diabetes Comorbidity Care Cascade (%)**

| **Category** | **Sex** | **Overall** | **Gambia rural** | **Gambia urban** | **SA rural** | **SA urban** | **Zimbabwe urban** |
| --- | --- | --- | --- | --- | --- | --- | --- |
| Both controlled | Men | 9.3 | 3.3 | 0.0 | 7.1 | 25.5 | 6.2 |
| Both controlled | Women | 17.9 | 0.0 | 0.0 | 19.1 | 33.3 | 23.3 |
| Both controlled | Overall | 14.8 | 1.5 | 0.0 | 14.7 | 30.7 | 17.4 |
| One controlled | Men | 33.3 | 16.7 | 25.6 | 30.4 | 44.7 | 46.9 |
| One controlled | Women | 35.0 | 28.6 | 20.3 | 40.4 | 40.9 | 38.3 |
| One controlled | Overall | 34.4 | 23.1 | 22.2 | 36.7 | 42.1 | 41.3 |
| Both treated | Men | 4.9 | 6.7 | 2.6 | 7.1 | 4.3 | 3.1 |
| Both treated | Women | 7.1 | 0.0 | 5.8 | 8.5 | 7.5 | 10.0 |
| Both treated | Overall | 6.3 | 3.1 | 4.6 | 8.0 | 6.4 | 7.6 |
| One treated | Men | 15.7 | 20.0 | 7.7 | 19.6 | 17.0 | 12.5 |
| One treated | Women | 21.9 | 20.0 | 31.9 | 27.7 | 10.8 | 20.0 |
| One treated | Overall | 19.6 | 20.0 | 23.1 | 24.7 | 12.9 | 17.4 |
| Both diagnosed | Men | 3.4 | 3.3 | 15.4 | 0.0 | 0.0 | 0.0 |
| Both diagnosed | Women | 3.1 | 2.9 | 10.1 | 0.0 | 2.2 | 1.7 |
| Both diagnosed | Overall | 3.2 | 3.1 | 12.0 | 0.0 | 1.4 | 1.1 |
| One diagnosed | Men | 8.3 | 13.3 | 15.4 | 3.6 | 2.1 | 12.5 |
| One diagnosed | Women | 7.1 | 20.0 | 21.7 |  | 2.2 | 1.7 |
| One diagnosed | Overall | 7.6 | 16.9 | 19.4 | 1.3 | 2.1 | 5.4 |
| Neither diagnosed | Men | 25.0 | 36.7 | 33.3 | 32.1 | 6.4 | 18.8 |
| Neither diagnosed | Women | 7.7 | 28.6 | 10.1 | 4.3 | 3.2 | 5.0 |
| Neither diagnosed | Overall | 14.1 | 32.3 | 18.5 | 14.7 | 4.3 | 9.8 |
| Total N | Men | 204 | 30 | 39 | 56 | 47 | 32 |
| Total N | Women | 351 | 35 | 69 | 94 | 93 | 60 |
| Total N | Overall | 555 | 65 | 108 | 150 | 140 | 92 |

# Table E: Medication and classes for antihypertensives

| Medication | Drug Classification | Indication for drug |
| --- | --- | --- |
| Amlodipine | Calcium channel antagonist | Hypertension |
| Doxazosin |  |  |
| Nifedipine |  |  |
| Captopril | ACE-inhibitor |  |
| Enalapril |  |  |
| Lisinopril |  |  |
| Perindopril |  |  |
| Ramipril |  |  |
| Losartan | Angiotensin Receptor Blocker |  |
| Telmisartan |  |  |
| Valsartan |  |  |
| Atenolol | Beta Blocker |  |
| Bisoprolol |  |  |
| Carvedilol |  |  |
| Methyldopa | Alpha-2 adrenergic receptor agonist |  |
| Hydralazine | Hydrazinophthalazine | Hypertension indication in conjunction with other antihypertensives |
| Furosemide | Loop diuretic |  |
| Spironolactone | Potassium-sparing diuretic |  |
| Bendrofluamethazide | Thiazide diuretic |  |
| Chlortalidone |  |  |
| Hydrochlorothiazide |  |  |
| Indapamide |  |  |

# Table F: Medication and classes for diabetes medications

| **Medication** | **Drug Classification** |
| --- | --- |
| Insulin | Insulin |
| Biguanides | Metformin |
| Glibenclamide | Sulfonylurea |
| Gliclazide |  |
| Glimepiride |  |

# Table G: Proportions of medication classes in the 3 countries

| **Country** | **Disease** | **Medication class** | **N (%)*** |
| --- | --- | --- | --- |
| **Overall** | **Diabetes** | **Biguanides** | **187 (70.8%)** |
|  |  | **Biguanides+ Sulfonylurea** | **41 (15.5%)** |
|  |  | **Sulfonylurea** | **17 (6.4%)** |
|  |  | **Insulin** | **10 (3.8%)** |
|  |  | **Insulin + Biguanides** | **9 (3.4%)** |
|  | **Hypertension** | **Thiazide diuretic** | **496 (50.2%)** |
|  |  | **Calcium channel antagonist** | **357 (36.1%)** |
|  |  | **ACE-inhibitor** | **75 (7.6%)** |
|  |  | **Loop diuretic** | **19 (1.9%)** |
|  |  | **Beta Blocker** | **15 (1.5%)** |
|  |  | **Angiotensin Receptor Blocker** | **14 (1.4%)** |
|  |  | **Potassium sparing diuretic** | **6 (0.6%)** |
|  |  | **Hydrazinophthalazine** | **4 (0.4%)** |
|  |  | **alpha-2 adrenergic receptor agonist** | **2 (0.2%)** |
| Gambia | Diabetes | Biguanides | 19 (51.4%) |
|  |  | Biguanides + Sulfonylurea | 8 (21.6%) |
|  |  | Sulfonylurea | 8 (21.6%) |
|  |  | Insulin | 2 (5.4%) |
|  | Hypertension | Calcium channel antagonist | 90 (57.7%) |
|  |  | Thiazide diuretic | 54 (34.6%) |
|  |  | ACE-inhibitor | 10 (6.4%) |
|  |  | Loop diuretic | 1 (0.6%) |
|  |  | alpha-2 adrenergic receptor agonist | 1 (0.6%) |
| South Africa | Diabetes | Biguanides | 130 (76%) |
|  |  | Biguanides+ Sulfonylurea | 25 (14.6%) |
|  |  | Insulin + Biguanides | 7 (4.1%) |
|  |  | Insulin | 5 (2.9%) |
|  |  | Sulfonylurea | 4 (2.3%) |
|  | Hypertension | Thiazide diuretic | 334 (69%) |
|  |  | Calcium channel antagonist | 67 (13.8%) |
|  |  | ACE-inhibitor | 55 (11.4%) |
|  |  | Loop diuretic | 10 (2.1%) |
|  |  | Beta Blocker | 7 (1.4%) |
|  |  | Angiotensin Receptor Blocker | 4 (0.8%) |
|  |  | Hydrazinophthalazine | 4 (0.8%) |
|  |  | Potassium sparing diuretic | 3 (0.6%) |
| Zimbabwe | Diabetes | Biguanides | 38 (67.9%) |
|  |  | Biguanides + Sulfonylurea | 8 (14.3%) |
|  |  | Sulfonylurea | 5 (8.9%) |
|  |  | Insulin | 3 (5.4%) |
|  |  | Insulin + Biguanides | 2 (3.6%) |
|  | Hypertension | Calcium channel antagonist | 200 (57.5%) |
|  |  | Thiazide diuretic | 108 (31%) |
|  |  | ACE-inhibitor | 10 (2.9%) |
|  |  | Angiotensin Receptor Blocker | 10 (2.9%) |
|  |  | Beta Blocker | 8 (2.3%) |
|  |  | Loop diuretic | 8 (2.3%) |
|  |  | Potassium sparing diuretic | 3 (0.9%) |
|  |  | alpha-2 adrenergic receptor agonist | 1 (0.3%) |
| *overlapping proportions | | | |

# Table H: Health-Related Quality of Life Utility Scores by care cascade stage

| **Disease** | **Category** | **Utility Score** | **95% CI** |
| --- | --- | --- | --- |
| Hypertension | Non-hypertensive | 0.85 | [0.84, 0.85] |
|  | Treated, Controlled | 0.81 | [0.81, 0.82] |
|  | Treated, Uncontrolled | 0.81 | [0.80, 0.82] |
|  | Diagnosed, Untreated | 0.78 | [0.77, 0.79] |
|  | Undiagnosed | 0.85 | [0.84, 0.85] |
| Diabetes | Non-diabetic | 0.83 | [0.83, 0.84] |
|  | Treated, Controlled | 0.80 | [0.78, 0.81] |
|  | Treated, Uncontrolled | 0.82 | [0.80, 0.85] |
|  | Diagnosed, Untreated | 0.80 | [0.78, 0.82] |
|  | Undiagnosed | 0.82 | [0.81, 0.83] |

# Table J: Health-Related Quality of Life Utility Scores by care cascade stage in the 3 countries

| **Disease** | **Category** | **Country** | **Utility Score** | **95% CI** |
| --- | --- | --- | --- | --- |
| Hypertension | Non-hypertensive | Gambia | 0.84 | [0.83, 0.84] |
|  | Non-hypertensive | South Africa | 0.85 | [0.84, 0.86] |
|  | Non-hypertensive | Zimbabwe | 0.87 | [0.86, 0.88] |
|  | Treated, Controlled | Gambia | 0.82 | [0.80, 0.83] |
|  | Treated, Controlled | South Africa | 0.81 | [0.80, 0.82] |
|  | Treated, Controlled | Zimbabwe | 0.81 | [0.80, 0.83] |
|  | Treated, Uncontrolled | Gambia | 0.79 | [0.78, 0.80] |
|  | Treated, Uncontrolled | South Africa | 0.81 | [0.80, 0.82] |
|  | Treated, Uncontrolled | Zimbabwe | 0.83 | [0.82, 0.85] |
|  | Diagnosed, Untreated | Gambia | 0.77 | [0.76, 0.78] |
|  | Diagnosed, Untreated | South Africa | 0.80 | [0.77, 0.84] |
|  | Diagnosed, Untreated | Zimbabwe | 0.85 | [0.82, 0.89] |
|  | Undiagnosed | Gambia | 0.83 | [0.82, 0.84] |
|  | Undiagnosed | South Africa | 0.85 | [0.83, 0.86] |
|  | Undiagnosed | Zimbabwe | 0.86 | [0.85, 0.87] |
| Diabetes | Non-diabetic | Gambia | 0.82 | [0.81, 0.82] |
|  | Non-diabetic | South Africa | 0.84 | [0.83, 0.84] |
|  | Non-diabetic | Zimbabwe | 0.85 | [0.85, 0.86] |
|  | Treated, Controlled | Gambia | 0.75 | [0.70, 0.80] |
|  | Treated, Controlled | South Africa | 0.81 | [0.79, 0.83] |
|  | Treated, Controlled | Zimbabwe | 0.78 | [0.74, 0.81] |
|  | Treated, Uncontrolled | Gambia | 0.83 | [0.78, 0.88] |
|  | Treated, Uncontrolled | South Africa | 0.81 | [0.79, 0.84] |
|  | Treated, Uncontrolled | Zimbabwe | 0.84 | [0.80, 0.89] |
|  | Diagnosed, Untreated | Gambia | 0.79 | [0.75, 0.82] |
|  | Diagnosed, Untreated | South Africa | 0.82 | [0.79, 0.84] |
|  | Diagnosed, Untreated | Zimbabwe | 0.74 | [0.68, 0.81] |
|  | Undiagnosed | Gambia | 0.82 | [0.81, 0.84] |
|  | Undiagnosed | South Africa | 0.81 | [0.79, 0.83] |
|  | Undiagnosed | Zimbabwe | 0.83 | [0.80, 0.86] |

# Table K: Differences in utility score between non-hypertensive or non-diabetic with care cascade stages

| **Disease** | **Contrast** | **Difference** |
| --- | --- | --- |
| Hypertension | Undiagnosed - Non-hypertensive | 0.001 (-0.009, 0.012) |
|  | Diagnosed, Untreated - Non-hypertensive | -0.066 (-0.079, -0.053) |
|  | Treated, Uncontrolled - Non-hypertensive | -0.034 (-0.045, -0.023) |
|  | Treated, Controlled - Non-hypertensive | -0.032 (-0.042, -0.021) |
| Diabetes | Undiagnosed - Non-diabetic | -0.011 (-0.026, 0.003) |
|  | Diagnosed, Untreated - Non-diabetic | -0.032 (-0.057, -0.007) |
|  | Treated, Uncontrolled - Non-diabetic | -0.007 (-0.034, 0.020) |
|  | Treated, Controlled - Non-diabetic | -0.036 (-0.057, -0.015) |

# Table L: Differences in utility score between non-hypertensive or non-diabetic and care cascade stages in the 3 countries

| **Table: Differences in EQ5D Index by Country and Disease** | | | |
| --- | --- | --- | --- |
| **Disease** | **Country** | **Contrast** | **Difference** |
| Hypertension | Gambia | Undiagnosed - (Non-hypertensive) | -0.005 (-0.023, 0.012) |
|  | Gambia | Diagnosed, Untreated - (Non-hypertensive) | -0.065 (-0.08, -0.051) |
|  | Gambia | Treated, Uncontrolled - (Non-hypertensive) | -0.045 (-0.063, -0.026) |
|  | Gambia | Treated, Controlled - (Non-hypertensive) | -0.018 (-0.04, 0.004) |
|  | South Africa | Undiagnosed - (Non-hypertensive) | -0.003 (-0.021, 0.016) |
|  | South Africa | Diagnosed, Untreated - (Non-hypertensive) | -0.047 (-0.088, -0.005) |
|  | South Africa | Treated, Uncontrolled - (Non-hypertensive) | -0.037 (-0.054, -0.02) |
|  | South Africa | Treated, Controlled - (Non-hypertensive) | -0.037 (-0.051, -0.023) |
|  | Zimbabwe | Undiagnosed - (Non-hypertensive) | -0.009 (-0.029, 0.011) |
|  | Zimbabwe | Diagnosed, Untreated - (Non-hypertensive) | -0.016 (-0.059, 0.027) |
|  | Zimbabwe | Treated, Uncontrolled - (Non-hypertensive) | -0.038 (-0.06, -0.016) |
|  | Zimbabwe | Treated, Controlled - (Non-hypertensive) | -0.057 (-0.079, -0.035) |
| Diabetes | Gambia | Undiagnosed - (Non-diabetic) | 0.006 (-0.013, 0.025) |
|  | Gambia | Diagnosed, Untreated - (Non-diabetic) | -0.03 (-0.072, 0.012) |
|  | Gambia | Treated, Uncontrolled - (Non-diabetic) | 0.014 (-0.046, 0.074) |
|  | Gambia | Treated, Controlled - (Non-diabetic) | -0.069 (-0.132, -0.005) |
|  | South Africa | Undiagnosed - (Non-diabetic) | -0.025 (-0.051, 0.001) |
|  | South Africa | Diagnosed, Untreated - (Non-diabetic) | -0.021 (-0.054, 0.012) |
|  | South Africa | Treated, Uncontrolled - (Non-diabetic) | -0.022 (-0.058, 0.014) |
|  | South Africa | Treated, Controlled - (Non-diabetic) | -0.028 (-0.053, -0.002) |
|  | Zimbabwe | Undiagnosed - (Non-diabetic) | -0.023 (-0.067, 0.021) |
|  | Zimbabwe | Diagnosed, Untreated - (Non-diabetic) | -0.108 (-0.191, -0.025) |
|  | Zimbabwe | Treated, Uncontrolled - (Non-diabetic) | -0.009 (-0.064, 0.046) |
|  | Zimbabwe | Treated, Controlled - (Non-diabetic) | -0.077 (-0.124, -0.031) |

# Figure B: Health-related quality of life utility score (with 95% confidence intervals) in different care cascade stages for hypertension and diabetes across the three countries. The broken line represents the median utility score.


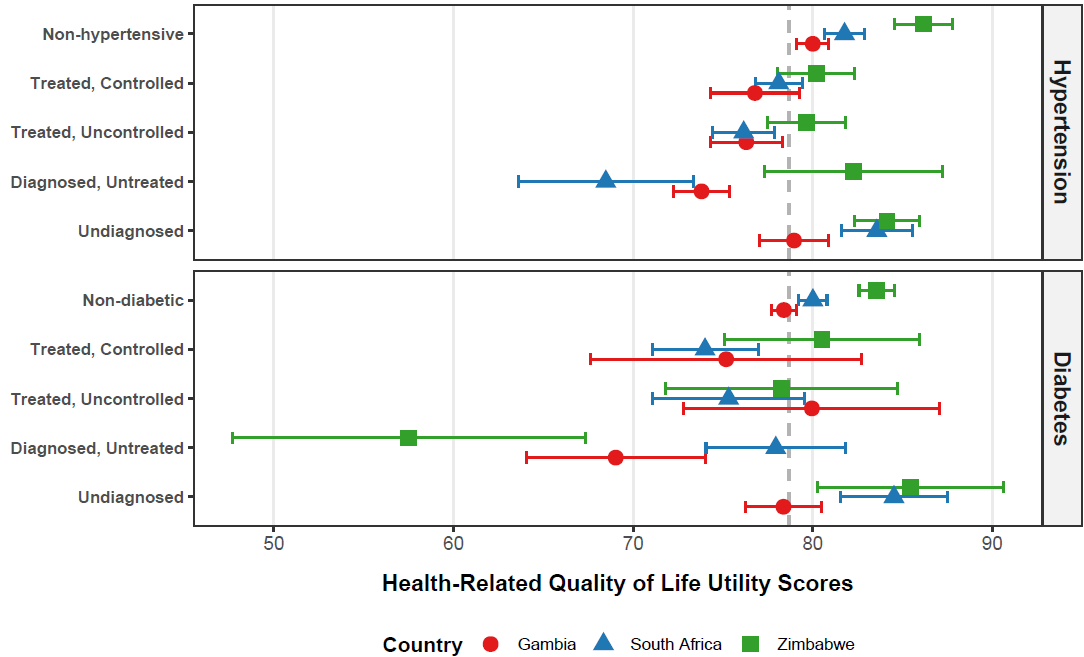


# Figure C: Health-related quality of life visual analogue scale score (with 95% confidence intervals) in different care cascade stages for hypertension and diabetes across the three countries. The broken line represents the median score


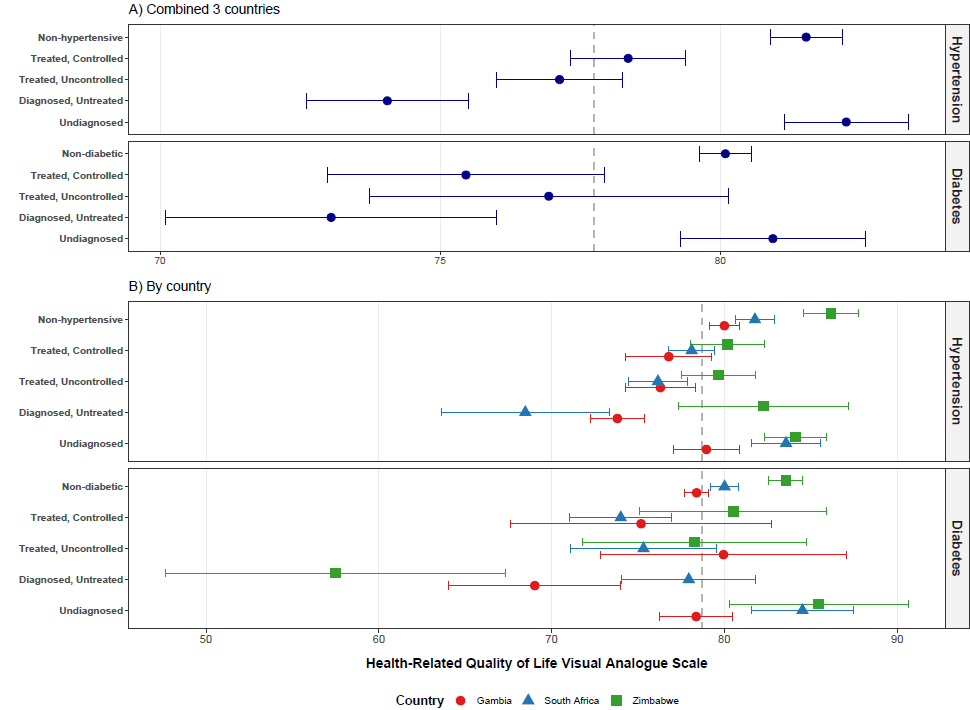


# Table M: Sensitivity analyses - differences in utility score (Ghanian value set) between non-hypertensive or non-diabetic and care cascade stages in the 3 countries

| **Table: Differences in EQ5D Index by Country and Disease - Sensitivity Analysis** | | | |
| --- | --- | --- | --- |
| **Disease** | **Country** | **Contrast** | **Difference** |
| Hypertension | Gambia | Undiagnosed - (Non-hypertensive) | -0.01 (-0.036, 0.016) |
| Hypertension | Gambia | Diagnosed, Untreated - (Non-hypertensive) | -0.099 (-0.121, -0.076) |
| Hypertension | Gambia | Treated, Uncontrolled - (Non-hypertensive) | -0.067 (-0.094, -0.04) |
| Hypertension | Gambia | Treated, Controlled - (Non-hypertensive) | -0.027 (-0.06, 0.005) |
| Hypertension | South Africa | Undiagnosed - (Non-hypertensive) | -0.006 (-0.034, 0.022) |
| Hypertension | South Africa | Diagnosed, Untreated - (Non-hypertensive) | -0.067 (-0.129, -0.005) |
| Hypertension | South Africa | Treated, Uncontrolled - (Non-hypertensive) | -0.052 (-0.078, -0.027) |
| Hypertension | South Africa | Treated, Controlled - (Non-hypertensive) | -0.052 (-0.074, -0.031) |
| Hypertension | Zimbabwe | Undiagnosed - (Non-hypertensive) | -0.011 (-0.041, 0.019) |
| Hypertension | Zimbabwe | Diagnosed, Untreated - (Non-hypertensive) | -0.019 (-0.083, 0.045) |
| Hypertension | Zimbabwe | Treated, Uncontrolled - (Non-hypertensive) | -0.053 (-0.086, -0.019) |
| Hypertension | Zimbabwe | Treated, Controlled - (Non-hypertensive) | -0.084 (-0.118, -0.051) |
| Diabetes | Gambia | Undiagnosed - (Non-diabetic) | 0.017 (-0.01, 0.045) |
| Diabetes | Gambia | Diagnosed, Untreated - (Non-diabetic) | -0.034 (-0.096, 0.029) |
| Diabetes | Gambia | Treated, Uncontrolled - (Non-diabetic) | 0.031 (-0.059, 0.12) |
| Diabetes | Gambia | Treated, Controlled - (Non-diabetic) | -0.098 (-0.193, -0.003) |
| Diabetes | South Africa | Undiagnosed - (Non-diabetic) | -0.038 (-0.077, 0) |
| Diabetes | South Africa | Diagnosed, Untreated - (Non-diabetic) | -0.037 (-0.087, 0.012) |
| Diabetes | South Africa | Treated, Uncontrolled - (Non-diabetic) | -0.04 (-0.094, 0.014) |
| Diabetes | South Africa | Treated, Controlled - (Non-diabetic) | -0.035 (-0.073, 0.003) |
| Diabetes | Zimbabwe | Undiagnosed - (Non-diabetic) | -0.03 (-0.096, 0.035) |
| Diabetes | Zimbabwe | Diagnosed, Untreated - (Non-diabetic) | -0.182 (-0.305, -0.058) |
| Diabetes | Zimbabwe | Treated, Uncontrolled - (Non-diabetic) | -0.017 (-0.098, 0.065) |
| Diabetes | Zimbabwe | Treated, Controlled - (Non-diabetic) | -0.117 (-0.186, -0.048) |

# Figure D: Sensitivity analyses using the Ghanian value set


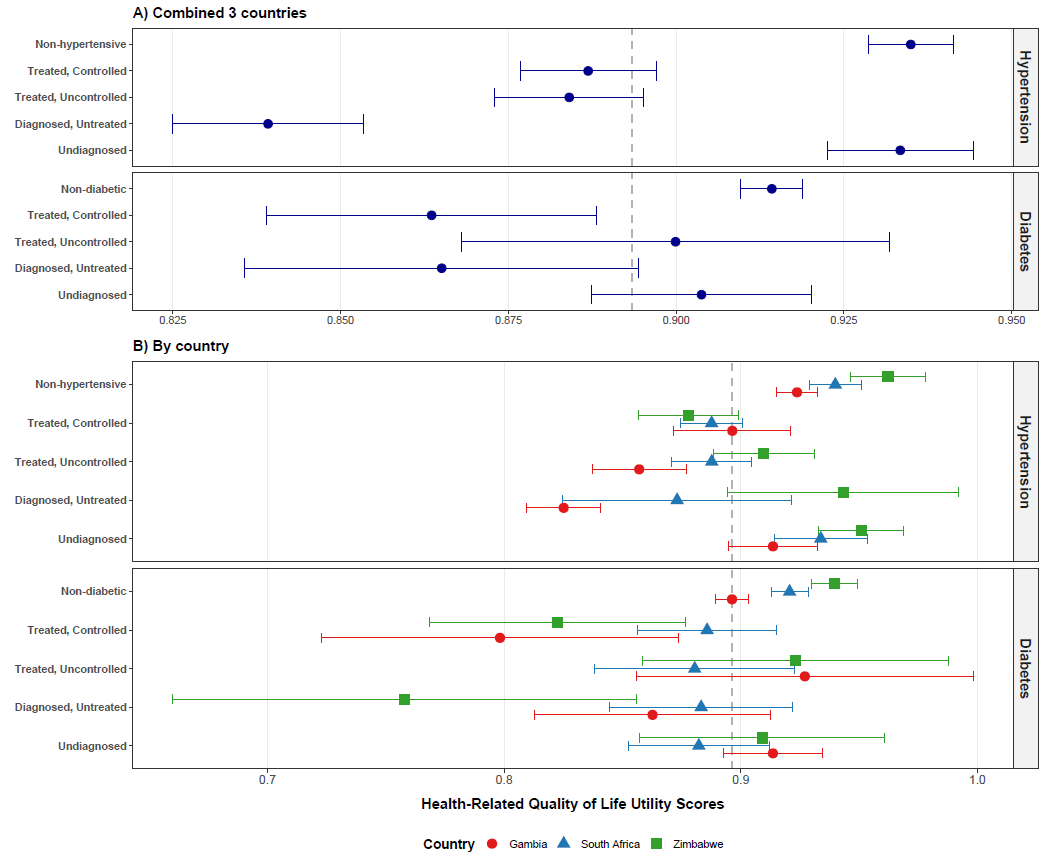


# Table N: Overall proportion (95% confidence intervals) with ≥1 diabetes complications

| **Diabetes Category** | **Prevalence (95% CI)** | **Cases/Total** |
| --- | --- | --- |
| Undiagnosed | 5.5% (3.1%-7.8%) | 20/365 |
| Diagnosed, Untreated | 19.5% (12.2%-26.8%) | 22/113 |
| Treated, Controlled | 29.2% (22.2%-36.2%) | 47/161 |
| Treated, Uncontrolled | 18.8% (10.9%-26.6%) | 18/96 |

# Table P: Prevalence of diabetes complications overall and in the three countries

| Country | N | CVD | CKD | Neuropathy |
| --- | --- | --- | --- | --- |
| Gambia | 2,270 | 64 (2.8%) | 10 (0.4%) | 1 (0%) |
| South Africa | 1,916 | 228 (12.2%) | 48 (2.7%) | 12 (0.6%) |
| Zimbabwe | 1,110 | 67 (6.1%) | 3 (0.3%) | 0 (0%) |
| **Total** | **5,296** | **359 (6.9%)** | **61 (1.2%)** | **13 (0.2%)** |
| CVD – cardiovascular diseases; CKD- chronic kidney disease | | | | |

# Table Q: Proportion (95% confidence intervals) with ≥1 diabetes complications across the three countries

| **Diabetes Category** | **Country** | **Prevalence (95% CI)** | **Cases/Total** |
| --- | --- | --- | --- |
| Undiagnosed | Gambia | 3.7% (1.2%-6.1%) | 8/219 |
|  | South Africa | 9.1% (3.7%-14.5%) | 10/110 |
|  | Zimbabwe | 5.6% (0.0%-13.0%) | 2/36 |
| Diagnosed, Untreated | Gambia | 15.4% (4.1%-26.7%) | 6/39 |
|  | South Africa | 20.3% (10.5%-30.2%) | 13/64 |
|  | Zimbabwe | 30.0% (1.6%-58.4%) | 3/10 |
| Treated, Controlled | Gambia | 17.6% (0.0%-35.8%) | 3/17 |
|  | South Africa | 33.3% (24.6%-42.1%) | 37/111 |
|  | Zimbabwe | 21.2% (7.3%-35.2%) | 7/33 |
| Treated, Uncontrolled | Gambia | 10.5% (0.0%-24.3%) | 2/19 |
|  | South Africa | 27.8% (15.8%-39.7%) | 15/54 |
|  | Zimbabwe | 4.3% (0.0%-12.7%) | 1/23 |
