# Supplementary material for: Hypertension and diabetes prevalence, associated factors, care cascade, and quality of life in older adults: A cross-sectional population-based study in The Gambia, South Africa, and Zimbabwe
Source: PLoS Med. 2026 Jul 7;23(7):e1004785. doi: 10.1371/journal.pmed.1004785 (PMC13367908; doi:10.1371/journal.pmed.1004785)
Supplement: Attachment — Submitted filename: Peer reviwew response.docx [file pmed.1004785.s006.docx]

**Authors response**

We thank the editors and reviewers for their interest in our work and very useful comments. We have responded to all comments using blue font in this document. The page numbers cited relate to the clean version of the manuscript

Reviewer #1: This manuscript reports a well-conducted, cross-sectional survey assessing prevalence, risk factors, care cascade stages, and health-related quality of life (HRQoL) for hypertension and diabetes across rural and urban sites in three countries. The study addresses an important public health topic and provides valuable comparative data.
The study is methodologically sound and addresses an important topic. With improved contextualization, clearer presentation of results, and deeper discussion of cross-country differences, the manuscript could make a significant contribution to the literature on NCD care in sub-Saharan Africa.

Thank you for noting the importance of our study and its potential to make a significant contribution.

Major Points:
1. Introduction and Context: The introduction is clear, but it omits some recent studies and reviews on hypertension and diabetes prevalence in Africa. Including or referencing these would help contextualize the study and clarify its added value.

Thank you for this suggestion. We have now looked through recent reviews (since 2022) related to hypertension and diabetes prevalence in Africa and revised the second paragraph to read, pages 6:

*In 2024, the IDF estimated the age-standardised prevalence of diabetes in Africa as 5%, although available evidence suggests that prevalence is higher in older adults. A 2016 meta-analysis reported a 14% pooled prevalence of diabetes among adults age ≥55 years in Africa [8]. Given the diverse diabetes phenotypes, a 2025 meta-analysis found a pooled prevalence of lean type 2 diabetes (individuals with body mass index [BMI] of <25 kg.m^2^) of 39% in people with a mean age of 48 years[9]. For hypertension, a series of meta-analyses have reported the pooled prevalence of diabetes in different age groups in Africa: 57% in adults aged≥50 years in 2019 [10]; 27% in rural and 34% in urban settings amongst West Africans of mean age 35-56 years [11]; and 58% in people living with diabetes with a mean age of 58 years in 2023 [12].*

2. Study Design and Implementation: Data were collected over nearly two years and embedded within a vertebral fracture study. Additional discussion of potential implications of this design (e.g., timing effects, site comparability) would improve interpretation.

Thank you for this suggestion. We have now added the following statement on pages 36:

*Finally, whilst data were collected over two years, healthcare delivery for hypertension and diabetes did not materially change over this period, so the effects of timing within and between countries would have been minimal. .*

3. Results Presentation: Some descriptive results, particularly for prevalence and HRQoL, are dense and difficult to follow. Simplifying sentence structure, standardizing presentation (country, value, confidence interval), and minimizing repetition would improve clarity. Adding a few interpretive comments in the results could help guide readers.

Thank you for this comment, which was shared by another reviewer. To make the results less dense, we have now provided the results pooled by age and removed the additional results relating to hypertension subtypes.

To improve readability, we have added interpretive comments in the prevalence results, which now read, pages 18-20:

*In total, the prevalence of hypertension was 55.6% (95% confidence intervals [CI] 54.2-56.9%). Across the study sites, prevalence was lowest in rural Gambia (39.6% (95% CI: 36.7-42.7)); >50% in urban Gambia (51.3% (48.5-54.2)) and urban South Africa (54.4% (51.3-57.6)); and >60% in rural South Africa (66.7% (63.6-69.7)) and urban Zimbabwe (66.9% (64.1-69.7)). The prevalence was higher among women in urban and rural South Africa and urban Zimbabwe (Figure 2, Table A in S1 Appendix). Across all study sites, prevalence was higher in the 40-54 age group compared to the ≥70 years age group (Figure A in S1 Appendix).*

*Overall, the prevalence of diabetes was 14.0% (95% CI: 13.1-15.0%). The overall prevalence was similar for the rural (12.8% (10.9-15.0)) and urban (13.1% (11.4-15.2)) Gambia, lower than for the rural (19.4% (16.9-22.1)) and urban (16.6% (14.3-19.1) ) South Africa, and higher than in urban Zimbabwe (9.2% (7.6-11.0)). Prevalence was higher in women in urban South Africa and Zimbabwe (Figure 2, Table A in S1 Appendix). Prevalence was higher in older age groups and both sexes in all sites, except for men in urban Zimbabwe and women in urban Gambia (Figure A in S1 Appendix). In all three countries, the prevalence of diabetes-hypertension comorbidity was 10.5% (9.7-11.3%). Prevalence was higher in rural (15.8% (13.6-18.3)) and urban (14.5% (12.3-16.8)) South Africa and lower in the other study sites albeit with overlapping confidence intervals in urban Gambia and Zimbabwe: 6.2% (4.8-7.8) in rural Gambia, 8.9% (7.3-10.6) in urban Gambia, and 8.3% (6.7-10.1) in urban Zimbabwe. The prevalence was higher among women in urban South Africa (Figure 2, Table A in S1 Appendix)*

For the HRQoL, besides adding words to help the flow (such as similarly, in contrast), we have split the results to four sections:

*Association between hypertension care cascade and quality of life;*

*Association between diabetes care cascade and quality of life;*

*Sensitivity analyses;*

*Association between the number of medicines, complications, and disease control*

4. Discussion and Interpretation: While findings align with existing literature, the manuscript could better highlight its unique contribution. Differences across countries could be discussed in the context of local health systems, policies, and capacities, which would strengthen the policy relevance of the study.

Thank you for this suggestion. We have attempted to discuss these differences when discussing the global diabetes targets using the following statements, pages 34:

*Moreover, achievement of the global diabetes targets varied across study sites and countries; for example, The Gambia and Zimbabwe were less likely to achieve them than South Africa. Whilst in South Africa, urban dwellers had better achievements than rural dwellers. Economic differences could explain these differences: firstly, at a country-level, South Africa is an upper-middle income country [18], secondly, at the individual-level, low household wealth was more common in rural than urban South Africa. Furthermore, differences could reflect differences in treatment guidelines. For instance, statins were not on The Gambia’s 2019 essential medicines list [53], potentially explaining the absence of treatment in people with diabetes. Overall, use of most of the antihypertensive medications (11/13 unique medications, 84.6%) and half of the diabetes medications (3/6 unique medications, 50.0%) listed in the 2025 WHO Model Lists of Essential Medicines[54] was reported. Similar to the successes in achieving 95-95-95 HIV targets across countries with wide-ranging economies [55], achievement of diabetes targets will require specific funding, stakeholder engagement, multicomponent interventions, and health systems realignment to prioritise screening, treatment and control of diabetes and hypertension [56].*

Reviewer #2: The authors should be commended for this extensive work aimed at characterizing the burden of hypertension and diabetes mellitus in three African countries. In the main, the prevalence of hypertension and diabetes mellitus remains high and control rates are poor. Authors should address the following comments:
1.QoL values should be expressed as percentages up to 1 decimal place.

Thank you for this comment. However, the standard practice when presenting HRQoL is as presented in the current manuscript, as values do not always range from 0 to 1, for example, the Zimbabwe value set has a maximum value of 0.9, and HRQoL can be negative when one feels they’d rather be dead than alive.

2. No Y-axis label for Figure 2

Thank you for spotting this, we have added a y-axis label, now Figure 3

3. No Y-axis label for Figure 3

We have removed this figure to reduce the number of results presented.

4. Table H: Metformin belongs to the Biguanides

Thank you for this suggestion. We have revised Metformin to Biguanides

Reviewer #3: SUMMARY
The manuscript presents a three-country population-based study (The Gambia, South Africa and Zimbabwe) investigating hypertension and diabetes prevalence and care cascade in adults over 40 years of age. Using a cross-sectional design across 3 countries, 5 settings (urban and rural settings in each country, except for rural Zimbabwe), the authors estimate the prevalence of hypertension and prevalence, the proportion of cases dropping out at different stages of the care cascade and investigate risk factors associated both with each condition but also each step in the care cascade. Further, they evaluate quality of life at different steps in the care cascade.
The authors found high prevalence in the studied populations for both conditions, in both rural and urban settings, and generally poor progression along the care cascade (but worse for diabetes), though this is less pronounced in South Africa.

PURPOSE OF REVIEW

This is a statistical review and focuses foremost on statistical rigour of the study, though I do add some less statistical comments.
OVERALL ASSESSMENT

This is an important study generating results / data on hypertension and diabetes (prevalence, risk factors, care cascade) where there are scant data and should be published.

Thank you for noting the importance of our study and it being publishable.

The study design is largely appropriate, though perhaps unclear why specifically The Gambia, South Africa and Zimbabwe were chosen. Taken together these results will not be representative of the wider sub-Saharan African landscape (though the authors do not claim this anyway), particularly because of the absence of Eastern Africa.

Thank you for this query. We have added this limitation on page 36:

*Furthermore, the lack of representation from Eastern and Central Africa regions limits the generalisability of the findings to the continent*

I do have some reservations about some of the analytical approaches used by the authors, however. The analysis is largely descriptive, and the descriptive analyses are, for the most part, all robust and appropriate. The one exception here are descriptive results for the pooled data across all countries as this is epidemiologically somewhat dubious as the five settings are quite distinct and the overall results depend on the relative sample sizes for each setting (see comment M2 below). More importantly, however, there is a very odd analytical choice to use aggregate-level meta-analysis for the association analyses for hypertension and diabetes, and this may need review (see comment M2 below). Further, there are absolutely no details provided in Methods about what specific meta-analysis model was used and so it is in general to assess how valid or not these analyses are (see comment M2 below). For care cascade analyses, GAM models are used but again details of these models
are lacking (see comment M4). It is also unclear whether the statistical analysis followed a pre-specified SAP. Taken together, there is a need for the manuscript to be revised with a much more detailed description of the statistical analysis and a justification for the main analytical choices.

As a study presenting observational data, the authors rightly follow STROBE guidelines for reporting results and helpfully appended a completed checklist. There are, however, minor issues with STROBE compliance (see below for specifics). Generally, on presentation of results, the main issue is the sheer abundance of results the study generated. For this reason, it seems the authors tried to focus on the most relevant results, at the risk of selective reporting - I feel that results on prevalence and care cascade could be presented more clearly with consistent reporting across conditions, care cascade steps and countries: e.g. i) overall results, ii) results per country , iii) results per age group, iv) results per country & age group, v) care cascade results consistently according to the 80-80-80, resp. 80-80-80-60, targets (see comment M5).

There are also occasional mistakes in some of the numbers reported (some examples in the detailed comments below), so there may be a need for the authors to carefully check all numbers for accuracy.

Thank you for these very useful comments. We have responded to each of them below.

MAJOR COMMENTS

M1. STROBE guideline deviations / necessary clarifications:
* Item 7 is OK, but details on which variables are considered exposures, confounders, mediators are listed only later on p.13/14.

Thank you for this comment. We have revised to read pages 9-14

* Items 12c, 12d, and 12e are not "N/A" and should be completed, even if just to state that data were complete (which numbers in Table 1 confirm is not the case), that the sampling strategy was ignored (as is the case) and that no sensitivity analyses were done. The latter may not be appropriate depending on what the authors did regarding missing data (which currently is not known from what is included in the manuscript). The aggregate-level meta-analysis (an odd analytical choice - see also comment M2) could also have been subjected to a sensitivity analysis to confirm results.

Thank you for this comment. We have now stated the following related to missing data on pages 15-16: *Overall, apart from waist circumference, which was not measured in The Gambia for cultural reasons, missingness in all other variables ranged from 0% to 7.5%, and no further analyses were conducted to handle missingness.*

Related to the sampling strategy, this was not ignored, the sampling was conducted as pre-specified in the study protocol, on page 8:

*A sample target of 504 women and 504 men in each site age ≥40 years (equally distributed across three age strata: 40-54, 55-69 and ≥70 years) provided ≥90% power (α=0.05) to estimate with 2.5% precision, an outcome with 9% prevalence (e.g. vertebral fracture) in each site, and detect an odds ratio of 2.0 for an associated risk factor with 13% prevalence (e.g. HIV) [21]. Further details on the study are described in the published protocol [21].*

Thank you for the suggestion regarding sensitivity analyses we have added the following on pages 14-15:

*Following peer review suggestion, two sensitivity analyses were explored: 1) use of logistic models adjusted for country instead of meta-analyses of the effect sizes from the five sites; and 2) use of continuous variables (for age, wealth index, BMI and MET minutes per week) and categories as captured in the questionnaire without binarisation for education, marital status and food insecurity.*

* Item 13a: Figure A should ideally be in the main body of the manuscript. Figure A suggests there may have been additional eligibility criteria (e.g. disability, frailty, ...) - can the authors clarify this? further Figure A implies that there were maximum recruitment targets per age-sex stratum which is not mentioned in Methods. Finally Figure A uses a different age categorisation than the rest of the manuscript, which is confusing.

Thank you for this suggestion. We have now added Figure 1 in the main manuscript to show the participant flow diagram. Some eligible participants declined to participate, this is detailed in Figure 1. We used age and sex stratified random sampling to recruit participants, as detailed in the published protocol. We have revised text on page 8:

*A sample target of 504 women and 504 men in each site age ≥40 years (equally distributed across three age strata: 40-54, 55-69 and ≥70 years)…….*

Based on another reviewer's suggestion, we now present the pooled results in the main manuscript, with age-stratified prevalence in the Appendix (Figure A).

* Item 14b: Table 1 does NOT list missing data proportions as claimed on the completed checklist. It only does so implicitly, and it is up to the reader to check for which variables there was how much missingness. There clearly were missing data as not all variables sum to the total number of records. There is also at least one small mistake in Table 1: 190/1052 = 18% not 17%.

Thank you for this comment. We have now revised Table 1 to show both the numerator and denominator and % to one decimal place

* Item 15: consider putting this in a single table for easier reference (e.g. similar to Tables A-F in the Appendix S1 but with counts) and include it in the main body of the manuscript.

Thank you for this suggestion. This is now covered in Table A in the appendix. This table presents the same results that are in Figure 2, we thought that, on balance, we would rather leave it in the Appendix and avoid duplication in reporting. However, we are open to having both Figure 2 and Table A in the main body or either of them, depending on editorial advice.

* Item 16b: This is not "N/A" and is given on p.11-12.

Thank you, edited to reflect this.

* Item 18: This spans more than just p.32.

Thank you. Revised to show p32-35.

* Item 19: There are some missing limitations in the discussion section - e.g. no mention is made of the restriction to 3 countries only which limits generalisability beyond these 3 countries, or the fact that the HRQoL value set for all countries was the one from Zimbabwe - not optimal for The Gambia or South Africa.

Thank you for this suggestion. The following limitation has been added on page 36: *Furthermore, the lack of representation from Eastern and Central Africa regions limits the generalisability of the findings to the continent*

On the HRQoL value set, we have added a limitation on page 36: *Moreover, the use of Zimbabwean and Ghanaian (for sensitivity analyses) HRQoL value sets was not optimal for The Gambia and South Africa.*

M2. The analysis largely presents descriptive statistics and basic association analyses stratified by country. However, there are cross-country analyses. Generally, the "overall" results are difficult to interpret as this depends on the relative sizes of the participant sets in the different countries and the split between urban / rural -- not clear what this "overall" population is representative of. This is however OK as it is clear what was done - the implication is mostly on interpretation but remains epidemiologically a bit dubious. Fairly puzzling, however, is the choice to use an aggregate-level meta-analysis (specific details of the meta-analysis model etc are missing from the manuscript - so difficult to be sure of details) for association analyses across countries. The only thing that is clear is that the meta-analysis must have been aggregate given that the authors state that there was no need to account for urban/rural setting as this was collinear with site.
Given that the authors had the individual-level data from each country, it is unclear why not simply a pooled analysis accounting for country was done (this would be equivalent to an IPD meta-analysis), which is the more obvious analytical choice and would have had better statistical properties.

Thank you for this comment. We feel the meta-analysis provides robust results, given that it generates effect sizes from all sites and aggregates them into one effect size along with a heterogeneity metric, presented along with the results in Table 2. This has been done in other studies, such as this analysis published in PLOS Medicine which we have cited, <https://doi.org/10.1371/journal.pmed.1003097>

We have explained the meta-analysis in more detail as suggested on page 13-14:

*Associations between hypertension and diabetes and sociodemographic and household characteristics (age, sex, marital status, educational attainment, food insecurity, wealth index) and lifestyle factors (BMI, WHR, tobacco use, alcohol consumption, physical activity) were assessed for each disease and site using logistic models, with outputs then meta-analysed using the ‘meta’ package [38]. This package derived both fixed- and random-effects pooled odds ratios, and the random-effects odds ratios were reported (the fixed- and random-effects odds ratios were relatively similar across all associations**). The Higgins I^2^ statistic, which shows heterogeneity across study sites in effect sizes rather than sampling error [39], was also derived and presented. Up to 40% heterogeneity is considered negligible, while up to 60% is considered moderate heterogeneity [39].* For the associations reported, we have added a line to note on moderate heterogeneity, page 23: *Associations between diabetes and overweight or obese BMI had moderate level of heterogeneity, >60% (Table 2).*

Additionally, we have done a sensitivity analysis using simple pooled analysis adjusted for country and found similar results, see page 21:

*Sensitivity analyses without meta-analyses but with adjustment for country yielded similar results (Table B1 in S1 Appendix).*

In the methods, page 14-15, we have added the following:

*Following peer review suggestion, two sensitivity analyses were explored: 1) use of logistic models adjusted for country instead of meta-analyses of the effect sizes from the five sites; and 2) use of continuous variables (for age, wealth index, BMI and MET minutes per week) and categories as captured in the questionnaire without binarisation for education, marital status and food insecurity.*

M3. The sampling involves sampling blocks for each site, then inviting everyone eligible within the blocks to participate in the study (until targets achieved). The analysis does not reflect this sampling strategy other than that some analyses are stratified by site. Did the authors account for block in any of the analyses and did this change any of the results substantially?

Thank you for this inquiry. As outlined in the manuscript, each randomly sampled block aimed to recruit 18-30 participants, we have revised our analysis to present age data categorised as per the three age strata used in this random block sampling. We do not have corresponding block data for each participant; it was not collected, as the random nature of block sampling would not be expected to introduce bias.

M4. The details on the GAMs used to interrogate associations between HRQoL and care cascade steps are missing - e.g. what smooth(s) with what parameters were used for which variables (given that the outcome in these models is HRQoL, most explanatory variables would be categorical - or was HRQoL the predictor not the outcome as stated on p.14)?

Thank you for this inquiry. HRQoL was indeed the outcome as stated, and no smoothing parameters were employed as the predictors were factor variables. We have checked, and the results are unchanged whether a linear model or a generalised linear model is used.

M5. There are 5 sites, 3 care cascade steps in hypertension and 4 in diabetes. This means there are potentially a lot of results to report for the case cascade. As such, the section on this, starting on p.22, probably has to be somewhat selective in what it reports. However, I found it very difficult to read as I failed to see a clear consistent way how this is reported other than focusing on statistically significant associations. I wonder if consistently reporting according to 80-80-80 (hypertension) and 80-80-80-60 (diabetes) would be easier to report. Also switching between percentage achieved and percentage missing (the gap) contributes to making this less straightforward to follow. As an example, overall control is reported consistently for hypertension and diabetes and both conditions combined but then switches to stating numbers/ percentages that dropped out at specific steps rather than reporting how many successfully completed each step which would directly speak to the targets. Also, for diabetes and both conditions combined, not all reasons for dropping out are stated unlike for hypertension. E.g. for diabetes, report overall control 21.9% (161/375), with 50.3% (370/735) of hypertensive participants diagnosed (gap with target: 29.7%), xx% (xx/735) treated, gap xx%, xx% (xx/735) controlled, gap xx%, and xx% (xx/xx) on statins, gap xx%.

Apologies for the hard-to-read results in this section, and thank you for the useful suggestions. Just to clarify, there are two sets of analyses that are related but different. The cascade of care is similar for hypertension and diabetes, and it looks at the proportions of those diagnosed, treated, and controlled among those living with each condition, hence, these proportions add up to 100%. The targets look at four different targets (or variables) for diabetes, and three for hypertension, and the denominators are different.

We have edited the care cascade results to read as follows for hypertension, page 24:

*For hypertension, the overall proportions were 31.0% (913/2944) for treatment and control; 26.0% (766/2,944) for treatment without control; 15.8% (465/2,944) for diagnosis without treatment; and 27.2% (800/2,944) for undiagnosed hypertension (Table C in S1 Appendix). Overall, 72.8% (2,144/2,944) were diagnosed and 57.0% (1,679/2,944) treated.*

For diabetes results, we have edited to read, page 25:

*For diabetes, the overall proportions were 21.9% 161/735) for treatment and control; 13.1% (96/735) for treatment without control; 15.4% (113/735) for diagnosis without treatment; and 49.7% (365/735) for undiagnosed hypertension (Table C in S1 Appendix). Overall, 50.3% (370/735) were diagnosed and 35.0% (257/735) treated.*

For hypertension targets, the revised text now reads, page 26:

*Overall, achievement of the 80-80-80% was: 72.8% (2144/2944) for diagnosis (gap, 7.2%), 78.3% (1679/2144) for treatment (gap, 1.7%), and 54.4% (913/1679) for control (gap, 25.6%).*

For diabetes targets, the revised text now reads, page 27:

*Overall, achievement of the 80-80-80-60% was 50.8% (377/742) for diagnosis (gap, 29.2%), 65.7% (243/370) for glucose control (gap, 14.3%), 60.4% (224/371) for blood pressure control (gap, 19.6%), and 17.8% (67/377) for statin use (gap, 42.2%).*

We note that, given the volume of results, we need to balance against the word count with no intention for selective reporting.

SPECIFIC POINTS

S1. Abstract / p.2: Is the definition of minimally important difference of 1 SD standard practice or why was this chosen?

Thank you for this inquiry. It is half an SD and is often used: we have cited the reference below in the methods, page 15:

Norman GR, Sloan JA, Wyrwich KW. Interpretation of Changes in Health-related Quality of Life: The Remarkable Universality of Half a Standard Deviation. Medical Care. 2003

This reference has been cited in recent meta-analyses such as:

<https://doi.org/10.1016/j.cpr.2026.102693>

<https://doi.org/10.1136/bmj-2025-085242>

S2. Abstract / p.3: As an example of the somewhat selective reporting (though aware this is the abstract and there is not that much space), the association between treated and controlled diabetes and HRQoL is only reported for The Gambia and Zimbabwe and South Africa (where this was not statistically significant) is not mentioned.

Thank you for raising this point and noting the abstract’s space limitations. Indeed, given the sheer number of results, we are not able to fit all results in the abstract, but we have provided all results transparently in the main manuscript or supplementary material. Unfortunately, adding this to the abstract would exceed the abstract word limit.

S3. Overall, the results for HRQoL and the care cascade seem to come a bit short in the abstract given that this was a key objective (e.g. see 3rd bullet in the author summary).

Thank you for this comment. As mentioned above, the abstract’s space limitations mean we are unable to have all results in the abstract. Nonetheless, the HRQOL results account for 30% of all results in the abstract in terms of word count.

S4. If most existing data have been on <50-year-olds, why was this study not restricted to >= 50 years of age?

Thank you for this question. We considered

S6. P.6, last paragraph: What is the exact difference between "untested" and "undiagnosed" - presumably the latter is untested diseased individuals whereas the former also includes healthy untested individuals? The rest of the manuscript does not really make a difference here (or only focuses on undiagnosed as opposed to untested).

Thank you for raising this. In this instance, we cite a paper that uses these terms, and we have now added more clarification from the cited paper to now read as follows: *(sum of those not tested, tested but unaware of their diagnosis, aware of their diagnosis but untreated, and treated but with uncontrolled diabetes)*

Testing relates to the question of *ever being tested for diabetes or hypertension*, while undiagnosed relates to *being tested and informed of a positive outcome*.

The rest of the manuscript does not use these terms, as we did not have data on testing, i.e. asking participants if they had ever been tested for diabetes or hypertension.

S7. P.6, last paragraph: The second sentence mentions 12 countries, states 2 percentages and lists 4 specific countries - so unclear for which countries the 80% and 62% refer to.

Thank you for this comment. We have now clarified from where the two percentages arise by saying, page 7:

*A large unmet need remains in the diagnosis, treatment, and control of both conditions. The estimated total unmet need (sum of those not tested, tested but unaware of their diagnosis, aware of their diagnosis but untreated, and treated but with uncontrolled diabetes ) for diabetes care in 12 African countries was approximately 80% as reported by Manne-Goehler et al [12] and 62% in Kenya, Burkina Faso, Ghana and SA as reported by Wade et al [13]*

S8. The rationale given on p.7 for doing this study in 3 countries only needs to be clearer. E.g. on p.6, 12 countries were mentioned, and unclear why no country from Eastern Africa was included and why 2 countries from Southern Africa were included for example.

Thank you for this query. As we mention in the introduction (page 8) the choice of the three countries was to have representation across the economic gradient based on income groups, page 7-8*: This study aimed to add to the existing literature on hypertension and diabetes and fill knowledge gaps based on data from three countries representing the Western (The Gambia) and Southern Africa regions (Zimbabwe, South Africa) and different World Bank income groups: low-income (The Gambia), lower-middle income (Zimbabwe), and upper-middle income (South Africa)[21].*

However, we note the lack of countries from other regions as a limitation on page 36:

*Furthermore, the lack of representation from Eastern and Central Africa regions limits the generalisability of the findings to the continent*

S9. P.8, sample size calculation: the statement regarding >90% power for detecting an OR of 2.0 or larger is not in correct; but in fact this is powered to detect on odds ratio as low as 1.74 with 90% power (the power for OR 2.0 is 98.6%).

Thank you for this comment. We use those metrics as they are the ones stated in the protocol, which we have cited. This allows for uniformity across papers that use the dataset. The stated power of >90% covers the 98.6%.

S10. P.11, first paragraph: Was there no other management of those with low blood glucose other than giving them a sugary snack?

Thank you for this question. Those with low blood glucose were also referred for clinical management. We have clarified this using the following statement on page 11

*Participants with an elevated blood glucose were referred to a local health facility for management, while those with a low blood glucose (<3.7 mmol/L) were promptly given sugary food/drink and referred by the study clinician for clinical management.*

S11. P.11, third paragraph: The binarisation of some variables is statistically not optimal as information is lost, power for detection associations is reduced and results will depend on the specific cut-off chosen. Did the authors do any sensitivity analyses to check the effect of changing the binary cut-off? A similar point pertains to the categorisation of continuous variables such as BMI on p.12.

Thank you for this comment. We have now added sensitivity analyses, which we have described in the methods, page 14-15:

*Following peer review suggestion, two sensitivity analyses were explored: 1) use of logistic models adjusted for country instead of meta-analyses of the effect sizes from the five sites; and 2) use of continuous variables (for age, wealth index, BMI and MET minutes per week) and categories as captured in the questionnaire without binarisation for education, marital status and food insecurity.*

We have noted the results of these analyses in the results section on page 21:

*Sensitivity analyses without meta-analyses but with adjustment for country yielded similar results (Table B1 in S1 Appendix). Using age, wealth index, BMI, and physical activity level as continuous variables yielded similar results (Table B2 in S1 Appendix). Without binarising categorical variables, compared with never married, being currently married was associated with diabetes after adjusting for sex, age, educational attainment, and wealth index: paOR, 1.96 (1.45-2.64, p<0.001) (Table B2 in S1 Appendix). Further, compared to answering No to food insecurity questions, answering Yes to one question was associated with diabetes: 1.35 (1.02-1.79, p=0.038) (Table B2 in S1 Appendix)*

S12. P.12, Quality of Life: If I read correctly, then the same value set for Zimbabwe was also used for The Gambia and South Africa? These are quite distinct populations and I would not be surprised if their value sets differed. I understand the pragmatic choice if a value set was only available for Zimbabwe, but was any sensitivity analysis done to check how much results would differ if other value sets from SSA would have been used for The Gambia and South Africa?

Thank you for this comment. We have now done a further sensitivity analysis using the recently published Ghanaian value set, see page 15:

*The same analysis was repeated using the visual analogue scale as the outcome, and sensitivity analysis used the utility scores from the Ghanaian value set [36] following peer review suggestions.*

While the scores are different, given that the Ghanian value set has a maximum score of 1 compared to 0.9 for the Zimbabwean value set, the trends and magnitude of difference between the care cascade stages are similar. We have added the following statement on page 30: *Trends and magnitude of differences in HRQoL were similar when the Ghanian value set was used (Figure D and Table N in S1 Appendix).*

S13. Statistical analysis section: Please specify whether there was a pre-specified Statistical Analysis Plan that was followed or not. If yes, where is this deposited / published? Were there any deviations from it?

Thank you for this inquiry. A prespecified analysis plan was not published; only the protocol was published.

S14. It is not incorrect to use a logistic regression model to estimate prevalence with corresponding 95% CI, but it would be more typical to compute exact binomial confidence intervals using the Clopper-Pearson approach. Why was the logistic regression approach chosen (which makes perfect sense for the association analyses, but is just a bit surprising for using it for estimation and yields point estimates that are not as intuitive as k/n)?

Thank you for this inquiry. We have recalculated the prevalences and the CIs using exact binomial intervals. The prevalences are similar to what we had stated. We have revised the statements on prevalence analyses to read on page 13:

*Prevalence estimates for hypertension, diabetes, and hypertension-diabetes comorbidity were calculated by study site, sex and age-group. The Clopper-Pearson exact binomial method* *(using the binom.test() function[37]) was used to compute 95% confidence intervals for all prevalence estimates.*

S15. Table 1: Add a count and % of missing data for each variable (or respectively the number and percentage of records for which each variable was available). It is clear that not all variables have complete records, e.g. for rural The Gambia, the numbers for educational attainment and BMI categories only sum to 1,045 not 1,052. Please also carefully check all numbers in this table; there is at least one error (e.g. tobacco use for rural The Gambia: even if all participants had this recorded, the percentage would be at least 190/1052 which is 18% not 17%).

Thank you for this feedback. We have revised the entire Table 1 to include the following: 1) counts and denominators of all percentages; 2) percentages to one decimal place rather than whole numbers; and 3) counts and percentages of missing data in each of the variables.

S16. Figure 1: Please add also the actual counts on top of each bar, in addition to the percentage which is already shown.

Thank you for this suggestion. We have now added counts and percentages at the top of bars.

S17. P.19, 2nd sentence ("The diabetes prevalence ..."): This statement is only correct in so far as a binary interpretation of statistical significance is made.  In all settings the diabetes prevalence is higher in the older age group, even if only statistically significantly so in urban South Africa.

Thank you for this comment. We agree this is the case, and we mention statistically significant results as we note in the methods on page 13: *Overall, confidence intervals were used to infer evidence of between-group differences.*

S18. Figure 4: This is a really nice, clear figure. While I am not sure that 'overall' is a very meaningful analysis population, but given how often it is being referred to in the text, it might be clearer if a bar for 'overall' is added to this figure. Also add counts on top of each bar (together with the percentages which are already shown) and totals (maybe at the bottom?).

Thank you for this suggestion. We have now revised the figure as suggested to include an ‘overall’ bar and to show the totals at the bottom of the bars

S19. Figure 5: 1) Unclear how meaningful the broken line / median is given that this is both across sites and conditions. I also though the fact that HRQoL for non-hypertensive and undiagnosed are broadly similar was quite interesting and would be worth commenting on.

We would like to keep the broken line to allow readers to have a reference for the population median. We have added a comment on the similarity between non-hypertensive and undiagnosed using the following statement on page 28:

*In contrast, being undiagnosed was broadly similar in HRQoL with being non-hypertensive (Figure 5).*

S20. P.30: Comparing controlled & treated to non-hypotensive / non-diabetic participants is appropriate. But I think this section could be improved by also commenting on comparisons between each failure in the case cascade and being treated and controlled - this is where improvements could be made by improving achieved levels on the care cascade pathway; i.e. add comparisons of undiagnosed vs treated & controlled, untreated vs treated & controlled and treated but uncontrolled vs treated & controlled.

Thank you for this comment. The comparisons made in this section were guided by MID, and only when MID was reached did we report the comparisons. We have clarified this using the following on page 15:

*Comparisons between being nondiabetic or non-hypertensive and care cascade categories were reported when MID was reached.*

S21. P.32: The sentence "Undiagnosed disease contributed most ..." needs rewriting. As written it seems to be at odds with Figure 4 for hypertension where the diagnostic gap is not that large (and control is much worse in both conditions).

Thank you for this comment. The statement is correct as it looks at the care cascade in Figure 3 (formerly Figure 2), which is a stacked bar, and yellow represents the undiagnosed disease, and not Figure 4, which is on the targets and has a different computation of the proportions. We have made clear it refers to Figure 3.

S22. P.34: For easier reading of this, it would help if the targets are restated somewhere on this page. Please also check stated percentages, e.g. the 29% on line 5 on this page seems to be 30% if properly rounded from the numbers on p.24 (735-365 = 370 diagnosed --> 50.3% --> gap of 29.7% to get to 80%), and the 20% from line 9 seems to be 25.4%, based on the numbers from p.22 (I get 913 controlled out of 1679 treated; i.e. 54.4% --> gap of 25.6% to get to 80% target).

Thank you for this suggestion. We have revised the statement to read, page 33:

*To standardise diabetes care, WHO member states recently adopted the global diabetes targets (80-80-80-60% in diagnosis, glucose control, hypertension control, and statin use), which aim to screen, treat, and manage diabetes (including a target on hypertension control) for all adults aged ≥40 years.*

The stated percentages may not add up, given that the two analyses – care cascade and gaps to targets – are slightly different, with the former being a stacked bar adding up to 100% from one variable having various categories while the latter uses standalone variables on each of the targets, hence the denominators may be different, leading to different percentages.

S23. P.35, last line: Causal inference is only precluded up to a point -- there are causal modelling techniques for observational, cross-sectional data (but of course these come at a cost and have limitations)...

Thank you for this comment. Given that causal inference methods were not used in this study, we prefer keeping the limitation as it is, and given it is already a very full paper, we would rather report the methodological approach as it is.

TYPOS & MINOR POINTS

T1. Abstract / p.3: "adults in The Gambia" reads a bit strangely as all participants were adults; I presume the authors meant "all participants in The Gambia"?

Thank you for this suggestion. We have revised to read, page 3: *Underdiagnosis and inadequate treatment and control of both diseases were more common in men and in both sexes in The Gambia.*

T2. P.8, second paragraph: replace "to detect, with 2.5% precision" with "to estimate, with 2.5% precision"; detection suggests some type of null hypothesis testing, but this is clearly about estimation not testing. Then add "to detect" in front of "an odds ratio of 2.0" in the next line.

The statement now reads, page 8: *A sample target of 504 women and 504 men in each site age ≥40 years provided ≥90% power (α=0.05) to estimate with 2.5% precision, an outcome with 9% prevalence (e.g. vertebral fracture) in each site, and detect an odds ratio of 2.0 for an associated risk factor with 13% prevalence (e.g. HIV) [15].*

T3. P.8, second paragraph: What has got "vertebral fracture" got to do with hypertension or diabetes?

This was a secondary analysis of a study whose primary aim was to determine the prevalence of vertebral fractures as implied by this statement on page 8: *This population-based cross-sectional study, conducted between 2022 and 2024, was embedded within a primary study of prevalent vertebral fractures and multimorbidity in adults age ≥40 years in five sites*

T4. Last line of p.10 and first two lines of p.11: This should be in the Results section.

Thank you for this suggestion. However, we would prefer this to be in the methods, as it was to determine the ‘validity’ of the methods of classifying someone as having undiagnosed diabetes in the study.

T5. P.15, last paragraph: "food insecurity was so in South Africa" --> "food insecurity was most common in South Africa"

Thank you, corrected to read ‘most common in South Africa’

T6. P.17, last sentence before "Factors associated with ...": It is not clear what the second half of this sentence is saying - can you rephrase?

Thank you for this comment. The sentence now reads: *The prevalence was higher among women in urban South Africa (Figure 2, Table A in S1 Appendix)*

T7. Table 2: Continuous colour legend scales might be clearer than just giving 2 levels for each scale. Consider also using different colours for protective ORs <1. What I^2 indicates is unclear as not described in Methods or Results - presumably this is the proportion of variation unexplained by sampling variation?

Thank you for this suggestion. We do not have protective factors per se in the results, as the protective odds ratios for smoking and alcohol consumption may not be actual and have been elaborated.

We have now clarified the I^2^ on page 14:

*The Higgins I^2^ statistic, which shows heterogeneity across the study sites in effect sizes as opposed to sampling error [11], was also derived and presented. Up to 40% heterogeneity is considered negligible, while up to 60% is considered moderate heterogeneity [11].*

T8. P.27: Something missing in the last sentence - what does 'of SDH...' and 'of IDH...' refer to?

We have now deleted this section in line with a reviewer suggestion to reduce the results presented

T9. P.34: "...as low household wealth.." --> "...low household wealth..."

Thank you: ‘as’ has been deleted

T10. P.34: "on a statin" --> "on statins"

Now reads ‘on statins’

T11. P.35, fifth/sixth line: "as it is influenced" --> "as they are influenced"
Thank you. We have revised to read as suggested

T12. P.35: better / lower quality of life - compared to what control? Healthy or undiagnosed but diseased or diagnosed and untreated, ...?

Thank you for this query. We have revised the statement to read, pages 34-35:

*Previous single-country studies have been conflicting; a study in Congo found that adherence to hypertension medication compared to non-adherence was associated with a better quality of life [16] , whereas a Nigerian study found that being on antihypertensives was associated with a lower quality of life compared to being untreated, whilst hypertension itself had no effect [17].*

T13. P.36, sixth line: "a single random blood glucose measurement" -- should this be "a single elevated random blood glucose measurement"

Thank you, revised to read as suggested

T14. P.36: "some, though very few" --> "some, most likely very few" (given that this is conjecture, albeit very reasonable, I think this should be expressed more cautiously)

Thank you, revised to read as suggested

T15. P.36: "Despite these limitations, [...]" I think this should be on a new paragraph.

Thank you for this suggestion. This has now been made into a new paragraph.

Reviewer #4: This paper used cross-sectional data from urban and rural sites in three African countries to estimate the prevalence of hypertension and diabetes, their risk factors, care continuum outcomes, medication use, and association with quality of life. Overall, the paper covers an important topic with a rare and valuable source of data. I provide my specific suggestions for improving the paper below.

Thank you for noting the importance of the study’s topic.

Motivation and Focus
The authors present a large number of analyses with varying degrees of motivation. This makes it hard to parse all the paper's findings and the central contribution of the paper becomes a bit lost. My suggestion would be to focus on a smaller subset of analyses that are more strongly and convincingly motivated in the introduction. Specifically:
1. For the prevalence and care continuum analyses, the authors need to directly confront what their paper brings above the STEPS and DHS data sources. I am not immediately familiar with whether their countries have STEPS/DHS data. If not, the authors should state this directly as a clear motivation; if yes, it would be very important that the authors then argue for what their analyses bring above these existing sources of data.

The DHS data primarily focuses on reproductive and maternal health, with only limited and self-reported data on noncommunicable diseases. For STEPS data sources, we have added the following statement on pages 7:

*While the WHO STEPwise approach to NCD risk factor surveillance (STEPS) surveys provide important evidence on hypertension and diabetes prevalence and associated factors, most of the surveys were conducted a decade ago, limited to adults below 70 years, and lacked data on quality of life [18].*

2. I found the hypertension subtypes and association of continuum outcomes with quality of life to be less well motivated. Beyond stating that these analyses don't exist for their countries, the authors don't convince the reader of why these are important to know. My suggestion would be to move these results to an appendix rather than focus on them in the main results as there is already quite a lot going on in the main paper.
In general, I felt the introduction could be significantly improved to be clearer about the main contributions of the paper.

Thank you for this comment. On reflection, we have removed the results on hypertension subtypes. Related to quality of life, we have added the following statement in the introduction, page 7:

*Patient-reported outcome measures, such as health-related quality of life, are important indicators of treatment efficacy and burden based on patients’ experiences [19]. However, the association between hypertension and diabetes treatment, control and quality of life remain understudied in Africa.*

Data and Sampling
Overall, I was impressed with the quality of data and the high response rates. The big open question I was left with was:
1. Are the data sub-nationally representative (e.g. do they represent the rural and urban populations of their countries), or, are among a selected population? The authors should provide more information on this in the main paper (rather than refer readers to an external protocol) as it has direct relevance for how widely applicable the findings are.

Thank you for this query. Indeed, the sites were representative of rural and urban sites in the study countries. We have added the following statement to clarify this on page 8:

*The study sites were representative of typical urban and rural sites and thus broadly representative of urban and rural sites in each country.*

Analyses and Results
1. As stated above, I found the analyses of hypertension subtypes and the association of continuum outcomes with quality of life to be not as well motivated or immediately relevant. I would suggest the authors move these to an appendix to bring more clarity to the results section.

Thank you for this suggestion. We have deleted the results on hypertension subtypes, as we will publish a more detailed article on hypertension subtypes.

2. Given the large number of results, I would suggest that the authors present the results pooled over age (rather than stratified by age group) in their main results, and discuss age differences in an Appendix. I found it hard to make sense of all the results given the number of stratifications.

Thank you for this suggestion. We have revised results to include the pooled results over age in the main results and those stratified by age group in the Appendix

3. I may have missed it, but the authors mention that they will meta-analyze their results in the methods section but I did not see those corresponding results in the text.

Thank you for this inquiry. We have now added the following to explain the meta-analysis in the methods, page 13-14:

*Associations between hypertension and diabetes and sociodemographic and household characteristics (age, sex, marital status, educational attainment, food insecurity, wealth index) and lifestyle factors (BMI, WHR, tobacco use, alcohol consumption, physical activity) were assessed for each disease and site using logistic models, with outputs then meta-analysed using the ‘meta’ package [38]. This package derived both fixed- and random-effects pooled odds ratios, and the random-effects odds ratios were reported (the fixed- and random-effects odds ratios were relatively similar across all associations). The Higgins I^2^ statistic, which shows heterogeneity across study sites in effect sizes rather than sampling error [39], was also derived and presented. Up to 40% heterogeneity is considered negligible, while up to 60% is considered moderate heterogeneity [39].*

4. The data on medications is rich and rare and I think the authors could exploit this more. Specifically, I was wondering if the number/type of medications was associated with control among those that report any treatment. Right now the authors briefly state that number of medications was not associated with control but I was left wondering which population this was among. This medication data could go a long way to understanding why there is such poor control among the treated (e.g is it adherence or under-dosed medications?)

Thank you for this comment. Given the large number of results already in the manuscript, we aim to develop a separate manuscript that delves deeper into medication adherence and dosage among those on treatment.

5. A minor point: given the high prevalence of both hypertension and diabetes, I would suggest that the authors present their regression results as risk ratios or risk differences, as the odds ratio is less appropriate here and is harder to interpret.

Thank you for this comment. We report odds ratios for two reasons: 1) we considered logistic models to be ideal for confounder-adjusted associations whose output is odds ratios; 2) to be comparable with other African studies that have used odds ratios to report on diabetes and hypertension-associated factors, such as the national Gambian study <https://doi.org/10.1016/S2214-109X(23)00508-9> and the AWI-Gen Study, <https://bmjopen.bmj.com/content/13/4/e069193>

and related meta- analyses which have used odds ratios for effect estimates: e.g.,

Ghana diabetes meta-analysis, <https://doi.org/10.1093/inthealth/ihy067>

Ethiopia hypertension meta-analysis, <https://doi.org/10.1371/journal.pone.0244642>

General editorial requests:

(Note: not all will apply to your paper, but please check each item carefully)

 We ask every co-author listed on the manuscript to fill in a contributing author statement, making sure to declare all competing interests. If any of the co-authors have not filled in the statement, we will remind them to do so when the paper is revised. If all statements are not completed in a timely fashion this could hold up the re-review process. If new competing interests are declared later in the revision process, this may also hold up the submission. Should there be a problem getting one of your co-authors to fill in a statement we will be in contact. Please do not add or remove authors without first discussing this with the handling editor. You can see our competing interests policy here: <http://journals.plos.org/plosmedicine/s/competing-interests>.

Thank you. Do let me know those who have not filled and I can follow up with them individually.

*Please upload any figures associated with your paper as individual TIF or EPS files with 300dpi resolution at resubmission; please read our figure guidelines for more information on our requirements: <http://journals.plos.org/plosmedicine/s/figures>. While revising your submission, we strongly recommend that you use PLOS's NAAS tool (<https://ngplosjournals.pagemajik.ai/artanalysis>) to test your figure files. NAAS can convert your figure files to the TIFF file type and meet basic requirements (such as print size, resolution), or provide you with a report on issues that do not meet our requirements and that NAAS cannot fix.

After uploading your figures to PLOS's NAAS tool -<https://ngplosjournals.pagemajik.ai/artanalysis>, NAAS will process the files provided and display the results in the "Uploaded Files" section of the page as the processing is complete.
If the uploaded figures meet our requirements (or NAAS is able to fix the files to meet our requirements), the figure will be marked as "fixed" above. If NAAS is unable to fix the files, a red "failed" label will appear above.
When NAAS has confirmed that the figure files meet our requirements, please download the file via the download option, and include these NAAS processed figure files when submitting your revised manuscript.

We have now used the NAAS tool to adjust figures to the specification and uploaded them separately. We still have the figures in the main manuscript for easy peer review.

*Please ensure that the paper adheres to the PLOS Data Availability Policy (see <http://journals.plos.org/plosmedicine/s/data-availability)>, which requires that all data underlying the study's findings be provided in a repository or as Supporting Information. For data residing with a third party, authors are required to provide instructions with contact information (web or email address) for obtaining the data. Please note that a study author cannot be the contact person for the data. PLOS journals do not allow statements supported by "data not shown" or "unpublished results." For such statements, authors must provide supporting data or cite public sources that include it.
We have these statements for on data availability:

*Data are available upon reasonable request. Researchers can access participant-level data via data.bris, provided ethical approvals are in place compliant with country-specific governance regulations.*

*We expect all researchers with submissions to PLOS in which author-generated code underpins the findings in the manuscript to make all author-generated code available without restrictions upon publication of the work. In cases where code is central to the manuscript, we may require the code to be made available as a condition of publication. Authors are responsible for ensuring that the code is reusable and well documented. Please make any custom code available, either as part of your data deposition or as a supplementary file. Please add a sentence to your data availability statement regarding any code used in the study, e.g. "The code used in the analysis is available from Github [URL] and archived in Zenodo [DOI link]" Please review our guidelines at <https://journals.plos.org/plosmedicine/s/materials-software-and-code-sharing> and ensure that your code is shared in a way that follows best practice and facilitates reproducibility and reuse. Because Github depositions can
be readily changed or deleted, we encourage you to make a permanent DOI'd copy (e.g. in Zenodo) and provide the URL.

We are working to upload the code on Github and should be ready before the paper is published

FORMATTING - GENERAL
*       Abstract: Please structure your abstract using the PLOS Medicine headings (Background, Methods and Findings, Conclusions). Please combine the Methods and Findings sections into one section.

We have adhered to this structure

*       At this stage, we ask that you include a short, non-technical Author Summary of your research to make findings accessible to a wide audience that includes both scientists and non-scientists. The Author Summary should immediately follow the Abstract in your revised manuscript. This text is subject to editorial change and should be distinct from the scientific abstract. Ideally each sub-heading should contain 2-3 single sentence, concise bullet points containing the most salient points from your study. In the final bullet point of 'What Do These Findings Mean?', please include the main limitations of the study in non-technical language. Please see our author guidelines for more information: <https://journals.plos.org/plosmedicine/s/revising-your-manuscript#loc-author-summary>.

We have added an Author Summary

*Please express the main results with 95% CIs as well as p values. When reporting p values please report as p<0.001 and where higher as the exact p value p=0.002, for example. Throughout, suggest reporting statistical information as follows to improve clarity for the reader "22% (95% CI [13%,28%]; p</=)". Please be sure to define all numerical values at first use.

We have now presented all 95% CI with p values when applicable and also defined all numerical values at first use

*Please include page numbers and line numbers in the manuscript file. Use continuous line numbers (do not restart the numbering on each page).

We have included page numbers and line numbers

Please cite the reference numbers in square brackets. Citations should precede punctuation.

Citations are made in square brackets with citation preceding punctuation

FIGURES AND TABLES
*Please provide titles and legends for all figures and tables (including those in Supporting Information files).

Table titles and figure legends provided after the Reference list

*Please define all abbreviations used in each figure/table (including those in Supporting Information files).

Abbreviations have been provided for figures and tables

*Please consider avoiding the use of red and green in order to make your figure more accessible to those with color blindness.

We have not used the green and red colors

SUPPLEMENTARY MATERIAL
*Please note that supplementary material will be posted as supplied by the authors. Therefore, please amend it according to the relevant comments outlined here.

*Please cite your Supporting Information as outlined here: <https://journals.plos.org/plosmedicine/s/supporting-information>
We have amended the supplementary material and cited it as S1 appendix in the manuscript

REFERENCES
*PLOS uses the numbered citation (citation-sequence) method and first six authors, et al.

* Please ensure that journal name abbreviations match those found in the National Center for Biotechnology Information (NCBI) databases (<http://www.ncbi.nlm.nih.gov/nlmcatalog/journals>), and are appropriately formatted and capitalised.
We have used the PLOS style of numbered citation and listing of authors

*Where website addresses are cited, please include the complete URL and specify the date of access (e.g. [accessed: 12/06/2023]).
*Please also see <https://journals.plos.org/plosmedicine/s/submission-guidelines#loc-references> for further details on reference formatting.

URL and date of access for websites have been provided

OBSERVATIONAL STUDIES

* Abstract: Please include the study design, population and setting, number of participants, years during which the study took place (enrollment and follow up), length of follow up, and main outcome measures.

Details on setting, number of participants and years the study took place have been provided. Follow-up time is not applicable. Main outcome measures have been included

*Please ensure that the study is reported according to the STROBE (or appropriate STOBE extension) guideline (available from: <https://www.equator-network.org/reporting-guidelines/strobe)> and include the completed STROBE (or STROBE extension) checklist as Supporting Information. Please add the following statement, or similar, to the Methods: "This study is reported as per the Strengthening the Reporting of Observational Studies in Epidemiology (STROBE) guideline (S1 Checklist)." When completing the checklist, please use section and paragraph numbers, rather than page numbers.

The study is reported according to the STROBE checklist which is provided as S1 Checklist

*[FOR POPULATION HEALTH/REGISTRY STUDIES] Please ensure that the study is reported according to the RECORD guideline (available from [[https://www.record-statement.org)]https://www.record-statement.org](https://www.record-statement.org)]https/www.record-statement.org)) and include the completed checklist as Supporting Information. Please add the following statement, or similar, to the Methods: "This study is reported as per the Reporting of Studies Conducted using Observational Routinely-Collected Data (RECORD) guideline (S1 Checklist)." When completing the checklist, please use section and paragraph numbers, rather than page numbers.

Not applicable

*[FOR POPULATION HEALTH ESTIMATES] Please ensure that the study is reported according to the GATHER statement (available from <https://www.equator-network.org/reporting-guidelines/gather-statement)> and include the completed checklist as Supporting Information. Please add the following statement, or similar, to the Methods: "This study is reported as per the Guidelines for Accurate and Transparent Health Estimates Reporting (GATHER) statement (S1 Checklist)." When completing the checklist, please use section and paragraph numbers, rather than page numbers.

Not applicable

*[FOR MEDIATION ANALYSES] We recommend that the study is reported according to the AGReMA statement (<https://agrema-statement.org/#:~:text=AGReMA%20is%20an%20evidence%2D%20and,randomised%20trials%20and%20observational%20studies>) and include the completed checklist as Supporting Information. Please add the following statement, or similar, to the Methods: "This study is reported as per the Guideline for Reporting Mediation Analyses (AGReMA) statement (S1 Checklist)." When completing the checklist, please use section and paragraph numbers, rather than page numbers.

Not applicable

*For all observational studies, in the manuscript text, please indicate: (1) the specific hypotheses you intended to test, (2) the analytical methods by which you planned to test them, (3) the analyses you actually performed, and (4) when reported analyses differ from those that were planned, transparent explanations for differences that affect the reliability of the study's results. If a reported analysis was performed based on an interesting but unanticipated pattern in the data, please be clear that the analysis was data driven.

We have provided the hypotheses at the end of the introduction using the study aims. We have described the analytical methods and analyses performed in the methods.

*Please state in the Methods section whether the study had a prospective protocol or analysis plan. If a prospective analysis plan (from your funding proposal, IRB or other ethics committee submission, study protocol, or other planning document written before analyzing the data) was used in designing the study, please include the relevant document(s) with your revised manuscript as a Supporting Information file to be published alongside your study and cite it in the Methods section. A legend for this file should be included at the end of your manuscript. If no such document exists, please make sure that the Methods section transparently describes when analyses were planned, and when/why any data-driven changes to analyses took place. Changes in the analysis, including those made in response to peer review comments, should be identified as such in the Methods section of the paper, with rationale.

A prospective analysis plan was not developed. We only cite the project protocol. We have noted additional analyses informed by peer review suggestions.
