# Supplementary material for: Hypertension and diabetes prevalence, associated factors, care cascade, and quality of life in older adults: A cross-sectional population-based study in The Gambia, South Africa, and Zimbabwe
Source: PLoS Med. 2026 Jul 7;23(7):e1004785. doi: 10.1371/journal.pmed.1004785 (PMC13367908; doi:10.1371/journal.pmed.1004785)
Supplement: Attachment — Submitted filename: Authors response_second review_APR.docx [file pmed.1004785.s007.docx]

**Authors’ response to second peer review feedback**

Reviewer #2: The authors have addressed all my comments excellently.

Thank you for taking the time to look at the revisions made.

Reviewer #3: I thank the authors for addressing many of my somewhat long list of comments from the previous round of review - the manuscript is much improved already. I still think this is an important and generally high-quality study that should be published. Nevertheless, I remain with some reservations that I think should be addressed before publication:

Thank you for this comment and for noting that the manuscript is much improved. We have provided a point-by-point response to the comments below.

Major comments:

1. My methodological comment on the meta-analysis approach remains. The authors have somewhat clarified the meta-analysis method used. It remains an aggregate-level meta-analysis, and is what is sometimes called a two-stage IPD meta-analysis. However, a one-stage IPD meta-analysis, which is achieved by using mixed effects models, is the gold standard in the literature when there is a limited number of studies / sites to be meta-analysed. I appreciate the paper cited by the authors, but I would have the same reservations for their analytical choice (I also note they had a much larger number of sites). The one-stage meta-analysis should essentially be what the authors have now included as a sensitivity analysis (though the full details of this model are not clearly enough described for me to be sure; though the text on l.326-327 does suggest a simple adjustment for country rather than treating site as a random factor was made - if so then this is not the IPD meta-analysis I suggested doing). To be clear: a one-stage, individual participant data analysis, treating site as a random factor in a mixed effects model, is a meta-analysis. The authors seem to have misinterpreted my comment as recommending to not do a meta-analysis. That was not what I was recommending, I just do not understand why the authors decided to use a less powerful approach to doing the meta-analysis than what is considered the gold standard meta-analysis - the data the authors have available would allow such a gold standard analysis. If the sensitivity analysis is this one-stage IPD meta-analysis, then I would suggest listing this as the primary analysis rather than the statistically less efficient two-stage approach where the meta-analysis component uses only aggregate data (and has a sample size of effectively just 5). To be very clear hear: the two-stage IPD meta-analysis approach currently used in the paper is not incorrect, it is just not what is currently considered to be the best approach. I do not expect there to be major differences in results, and I will not insist on this point if the authors absolutely want to stick with the current approach - I am just trying to nudge the authors to revise their analysis so as to use a slightly better approach.

Thank you for this suggestion and the nudge to revise our analyses. Given the similarity of results with our earlier approach, we have now used the one-stage IPD meta-analysis and deleted results associated with the two-stage IPD meta-analysis. The revised one-stage IPD accounts for the hierarchy sampling strategy as described in the second comment below. The results are provided in a revised Table 2, which provides odds ratios and p-values (instead of the heterogeneity measure metric).

2. My comment as to the sampling strategy being ignored related to the analysis not accounting for it rather than it not being described in the manuscript (which it is). Many of the reported analyses, as described currently at least, do not account for the stratified (by site, sex and age) or the block-randomised sampling process that was used to recruit participants into the study. Obviously, some analyses are stratified and/or account for sex and age and that is fine. The sampling strategy is described in the paper, it just does not seem to have been taken into account for all analyses. For many of the descriptive analyses this is fine, but for analyses using regressions, ideally the design factors should have been accounted for. As such, I disagree with the authors' response to my original comment M3. A principled analysis would account for the blocks used in the random block sampling - effectively this introduces clustering which means that the assumption of independent data observations that virtually all standard statistical analyses make is not met and hence inference would technically not be valid. I do not expect that this would make a huge difference, but it should technically be accounted for. Related to this item 12d from the STROBE checklist should not be left as N/A.
To be very clear on this point: not accounting for the blocks is technically not correct. I do not expect that there is any strong cluster effect and hence do not expect that accounting for the blocks would make much of a difference to results. There is however a risk of underestimated standard errors and too low p-values etc here. So I urge the authors to account for this in their analyses as otherwise these would not be technically valid. If this is not possible due to the block information not having been recorded (but surely it must have given that it was required for sampling?), then this should be clearly stated.

Thank you for this comment. We have now merged the requisite datasets required to revise the logistic regression analysis to account for the hierarchy sampling strategy. We have used site rather than country as the block numbers are unique for each site in the sites’ sampling areas. We have included details of this strategy using the following text on pages 13-14:

*Associations between hypertension and diabetes and sociodemographic and household characteristics (age, sex, marital status, educational attainment, food insecurity, wealth index) and lifestyle factors (BMI, WHR, tobacco use, alcohol consumption, physical activity) were assessed for each disease using Generalised Linear Mixed Effects Models (GLMM) with study site as a fixed effect and a random intercept for sampling block, nested within sampling area, nested within site, to account for the hierarchical sampling structure. Model estimation used the Laplace approximation with the BOBYQA optimiser set to a maximum of 200,000 function evaluations to facilitate convergence using the ‘lme4’ package [38].*

We have revised the STROBE checklist to reflect that 12d is now reported in the Statistical analysis section, paragraph 2.

Other comments:

3. The authors did not respond to my comment that the "overall" analysis is epidemiologically a bit dubious as this would not be reflective of any particular population given the dependence on the relative size of recruitment from each site etc. I do not suggest this should be removed from the paper and it is clearly described, but I think somewhere this caveat should be stated clearly - perhaps in the limitations in the discussion? This is related though distinct from the lack of generalisability of results to the wider SSA region given the lack of Eastern and Central African sites; but could possibly be mentioned in the same limitation section.

Apologies for not responding to the comment fully and thank you for the clarification. We have added the following statement in the limitations section, page 40: *For clarity, the ‘overall’ findings in the current study are not reflective of a particular population but rather the combined sample from all study sites.*

4. Regarding the description of models fitted as generalised additive models (GAMs) on lines 337 to 346, and given the explanation in the author's rebuttal letter that none of these models had continuous predictor variables, this means that the GAMs were just simple generalised linear models (GLMs) and should be described as such. It is not incorrect to call them GAMs, given that GAMs are extending GLMs, but it is a bit misleading to refer to a more complex model that is specifically designed to allow flexible modelling of continuous predictors when no such predictors are in fact in the models.
Thank you for this comment. We have now revised to refer to the models as generalised linear models (GLMs) on page 15.

5. The response to my original comment S4 seems to have been inadvertently cut off and is incomplete.

Apologies for this, we missed this incompleteness. The comment was: S4. If most existing data have been on <50-year-olds, why was this study not restricted to >= 50 years of age?

Thank you for this question. The study was not restricted to <50 years for the following reasons: 1) the 40-49 age group corresponds to a period of middle age that is characterised by with increased hypertension and diabetes risk, 2) restriction would disassemble the age-groups used to structure the sampling strategy.

6. As response to my original comment S13, the manuscript should briefly state within the manuscript whether or not analyses followed a pre-specified SAP (it would have been better if this had been published, but if there was a SAP and it was followed this should still be stated in the manuscript; this will have implications for how readers interpret results).

Thank you for clarifying this point. We have now added the following statement, on page 40: *Whilst analyses were pre-planned, the statistical analysis plan was not pre-specified.*

7. In my original comment S23, I was not suggesting that the authors do a causal analysis, it is just that the statement that a causal analysis is not possible due to this being a cross-sectional study is not correct; this could be done if this had been the intention of the authors. So, I still think the statement on what is now l.625-626 should be reworded to clarify that not doing a causal analysis was an analytical choice rather than an inevitability due to the study design. There are valid reasons for this choice: causal inference using observational data can be challenging, is often unconvincing due to strong assumptions and often not the point of the study objectives anyway.

Thank you for this clarification and suggestion. We have edited the limitation, on page 39, to read:

*Firstly, the choice not to undertake a causal inference analysis precludes any causal claims about the observed associations.*

8. I did not earlier appreciate, on what is now Figure 1, the 44 "additional eligible attendees" participants listed in the bottom section. Where did these come from and why were these not screened and invited like other participants?

Thank you for this comment. The additional eligible attendees were caregivers from the same household who accompanied the invited participants and who requested to participate. We have added the following in the Figure caption: *The 44 additional eligible attendees were members of the same household who accompanied the invited participants in a caregiving capacity.*

Reviewer #4: I thank the authors for taking my comments seriously. I believe the revised manuscript is a significant improvement. I had a few remaining minor comments:

Thank you for noting that the manuscript has significantly improved. We have addressed the comments below.

1. Overall, the revised introduction is much improved and clearer. The authors introduced new text based on my comment to motivate the analyses with health-related quality of life. This new text is good, however, it is placed in the middle of a paragraph, which interrupts a discussion of other data gap and breaks up an otherwise clear thought. I would suggest that the authors move this new text to a small paragraph following paragraph 3 in the intro to keep the ideas together.

Thank you for this comment and noting that the introduction is improved and clearer. We have moved the text motivating the health-related quality of life into a new paragraph. This paragraph now includes the sentence that summarises the knowledge gaps.

2. Lines 334-336 still reference the now removed subtype analysis. This was probably just a minor typo, but it should be removed.

Thank you for spotting that reference has now been deleted.

3. When describing the association, odds ratio, results, the authors should clearly state what the reference groups are. This information is in the table but I found myself asking "greater among those aged 55-69 compared to whom?"

Thank you for this comment. We have now revised the text to provide the reference groups on page 21:

*Independent of study site, sex, educational attainment and wealth index, being 55-69 years, compared to being 40-54 years, was associated with higher odds of both hypertension (2.71 (2.34;3.13), p<0.001) and diabetes (1.84 (1.48;2.30), p<0.001). Similarly, being ≥70 years, compared to being 40-54 years, was associated with both hypertension (6.59 (5.58;7.79), p<0.001) and diabetes (2.24 (1.79;2.82), p<0.001) (Table 2).*

4. I had two comments on the regression tables: (1) I personally did not understand the value of the shading, and this actually confused me as I initially assumed it was relating to the size of the p-value; (2) what is the value of showing model 1,2, and 3? My suggestion would be to just show the fully adjusted model 3 and put the others in the appendix if needed. This feels more intellectually robust while also streamlining the text and interpretation.

Thank you for these comments. In the revised table following a new analysis, we have removed the shading. On the second suggestion, we would prefer to report all 3 models, as is standard practice, rather than reporting only the fully adjusted model. To make the manuscript more concise, given the breadth of results, we only mention the fully adjusted model in the text, but readers can see the results of the other two models. However, should the editorial office feel strongly about this, we are happy to trim the table to only include the third model.

5. Lines 448-458: As the authors are now presenting the medication results in another paper, I wondered if this paragraph should be removed and saved for that paper. At a minimum, there should be a mention of this analysis in the methods section.

Thank you for this suggestion. We feel this paragraph provides largely descriptive data that further enriches the care cascade data and would not be the main focus of the other paper; we therefore would rather keep it in, it adds value. We have included a statement in the statistical analysis section to describe the analysis, on page 15:

*Using the previously described definitions, descriptive analyses (counts and percentages) were conducted for hypertension and diabetes medications, and results for diabetes complications were presented in the text or tables.*
