# Supplementary material for: Hypertension and diabetes prevalence, associated factors, care cascade, and quality of life in older adults: A cross-sectional population-based study in The Gambia, South Africa, and Zimbabwe
Source: PLoS Med. 2026 Jul 7;23(7):e1004785. doi: 10.1371/journal.pmed.1004785 (PMC13367908; doi:10.1371/journal.pmed.1004785)
Supplement: Attachment — Submitted filename: Minor revision response.docx [file pmed.1004785.s008.docx]

**Authors’ response to minor revision**

Thank you for your time in reviewing our manuscript. We have now responded to each of the comments using blue font in the document below.

Technical requests

1) Please ensure that the funders and grant numbers match between the Financial Disclosure field and the Funding Information tab in your submission form. Note that the funders must be provided in the same order in both places as well.
   Currently, order of grants are different in both places.

We have now revised to have the funders in the same order.

2) We do not publish any copyright or trademark symbols that usually accompany proprietary names, e.g., (R), (C), or TM  (e.g. next to drug or reagent names). Please remove all instances of trademark/copyright symbols throughout the text, including © on line no 787,Seca® on line no 267, 270

We have now removed the trademark symbols that were associated with Seca

3) Please label your SI Tables in the numerical order in the uploaded SI file(S1 Appendix). Currently (Table H,I,O,P) are missing.
Please check and amend the changes accordingly

We have now amended the numbering of the S1 tables

4) In your Data Availability Statement, please ensure you have provided all necessary access information in complete sentences. Access information should include the name of any data repositories used as well as digital object identifiers (DOIs), accession numbers or codes, or other persistent identifiers for all relevant data. If all data has been provided within your submission files, please use the statement "All relevant data is available within the manuscript and Supporting Information files." The Data Availability Statement will appear on the published version of your manuscript

We have now revised the Data Availability Statement to read: *Data used in the current study is stored on a secure, password-protected University of Bristol network filestore space. Data are available upon reasonable request. Third-party researchers wishing to access individual data records will be able to apply the following processes outlined at* [*http://www.bristol.ac.uk/staff/researchers/data/accessing-research-data/*](http://www.bristol.ac.uk/staff/researchers/data/accessing-research-data/)*, subject to a data transfer agreement and informed consent procedures. Shared will be anonymised and contain no information that could directly identify any of the participants. Data users will need to acknowledge data sources and ensure the regulatory requirements of relevant ethical bodies are met. The contact for inquiries is* [*data-bris@bristol.ac.uk*](mailto:data-bris@bristol.ac.uk)*.*

GENERAL EDITORIAL REQUESTS

* Please confirm that your title complies with PLOS Medicine's style. Your title must be nondeclarative and not a question. It should begin with main concept if possible. "Effect of" should be used only if causality can be inferred, i.e., for an RCT. Please place the study design ("A randomized controlled trial," "A retrospective study," "A modelling study," etc.) in the subtitle (ie, after a colon).

We confirm that the title complies with PLOS Medicine's style. The title reads: *Hypertension and diabetes prevalence, associated factors, care cascade, and quality of life in older adults: cross-sectional population-based study in The Gambia, South Africa, and Zimbabwe*

* Please confirm that your abstract complies with our requirements, including format (three sections: Background, Methods and Findings, and Conclusions) and providing all the information relevant to this study type <https://journals.plos.org/plosmedicine/s/submission-guidelines#loc-abstract> 

We confirm the abstract complies with the format requirements and provides all information relevant to this study

* In the abstract, please include the important dependent variables that are adjusted for in the analyses.

We have provided the dependent variables adjusted for using the following statement: *Associations between hypertension and diabetes and risk factors were assessed using study site, age, sex, educational attainment, and wealth index-adjusted Generalised Linear Mixed Effects Models*.

* Please ensure that the Introduction ends with a clear description of the study question or hypothesis.

The Introduction ends with a statement of the following questions, on page 8:

*Specifically, this study aimed to answer the following questions: 1) What is the prevalence of hypertension, diabetes and comorbid diabetes and hypertension across urban and rural sites? 2) Which sociodemographic, household and lifestyle factors are associated with hypertension and diabetes? 3) What is the achievement status on the stages of the hypertension and diabetes care cascades, including attainment of African hypertension targets and global targets for diabetes? and 4) What are the associations between different care cascade stages (diagnosis, treatment and control) and health-related quality of life?*

* Please ensure that all abbreviations are defined at first use throughout the text.

We have defined all abbreviations at first use

* Please confirm that all numbers presented in the abstract are present and identical to numbers presented in the main manuscript text.

We confirm that all numbers in the abstract are identical to the numbers in the main manuscript

* Statistical reporting: Please revise throughout the manuscript, including tables and figures.
- Please report statistical information as follows to improve clarity for the reader (95% CI [13,28]; p</=).
- Please separate upper and lower bounds with commas instead of hyphens as the latter can be confused with reporting of negative values.
- Please repeat statistical definitions (HR, CI etc.) for each set of parentheses.

Thank you for this suggestion. We have now revised our reporting to follow this format.

GENERAL

FUNDING STATEMENT
* The funding statement should include: specific grant numbers, initials of authors who received each award, URLs to sponsors’ websites. Also, please state whether any sponsors or funders (other than the named authors) played any role in study design, data collection and analysis, the decision to publish, or preparation of the manuscript. If they had no role in the research, include this sentence: “The funders had no role in study design, data collection and analysis, decision to publish, or preparation of the manuscript.”

* It appears that one or more study authors may be affiliated with one or more of the agencies that funded the study (i.e. MRC) Thus, the statement “The funders had no role in study design, data collection and analysis, decision to publish, or preparation of the manuscript” does not apply. Please revise the Financial Disclosure accordingly, as in "[Author name] is [author's role] at [funding agency]. The funders had no other role in study design…..”

We have now included the funders' URLs in the funding statement. No author is affiliated with a funding agency; they are from a research unit that has received core funding from the MRC, hence the naming of the unit after the MRC, but they do not work for the funder: MRC.

COMPETING INTERESTS STATEMENT
* All authors must declare their relevant competing interests per the PLOS policy, which can be seen here: <https://journals.plos.org/plosmedicine/s/competing-interests> For authors with ties to industry, please indicate whether any of the interests has a financial stake in the results of the current study.
*Please amend your competing interests statement. Funding information should go to the relevant section and include URLs.

The authors do not have competing interests to report, and we have now included the URLs related to funding sources

DATA AVAILABILITY
* PLOS Medicine requires that the de-identified data underlying the specific results in a published article be made available, without restrictions on access, in a public repository or as Supporting Information at the time of article publication, provided it is legal and ethical to do so. Please see the policy at

<http://journals.plos.org/plosmedicine/s/data-availability> 

and FAQs at

<http://journals.plos.org/plosmedicine/s/data-availability#loc-faqs-for-data-policy>

* PLOS defines the “minimal data set” to consist of the data set used to reach the conclusions drawn in the manuscript with related metadata and methods, and any additional data required to replicate the reported study findings in their entirety. Authors do not need to submit their entire data set, or the raw data collected during an investigation. Please submit the following data:

The values behind the means, standard deviations and other measures reported;
The values used to build graphs;
The points extracted from images for analysis.

* The Data Availability Statement (DAS) requires revision. For each data source used in your study:

a) If the data are freely or publicly available, note this and state the location of the data: within the paper, in Supporting Information files, or in a public repository (include the DOI or accession number).

b) If the data are owned by a third party but freely available upon request, please note this and state the owner of the data set and contact information for data requests (web or email address). Note that a study author cannot be the contact person for the data.

c) If the data are not freely available, please describe briefly the ethical, legal, or contractual restriction that prevents you from sharing it. Please also include an appropriate contact (web or email address) for inquiries (again, this cannot be a study author

Given the ethical restrictions, the data used in the study cannot be made freely available but can be shared upon reasonable request and approval from the country study ethics leads. We have revised the Data Availability statement to read, on page 41: *Data used in the current study is stored on a secure, password-protected University of Bristol network filestore space. Data are available upon reasonable request. Third-party researchers wishing to access individual data records will be able to apply the following processes outlined at* [*http://www.bristol.ac.uk/staff/researchers/data/accessing-research-data/*](http://www.bristol.ac.uk/staff/researchers/data/accessing-research-data/)*, subject to a data transfer agreement and informed consent procedures. Shared will be anonymised and contain no information that could directly identify any of the participants. Data users will need to acknowledge data sources and ensure the regulatory requirements of relevant ethical bodies are met. The contact for inquiries is* [*data-bris@bristol.ac.uk*](mailto:data-bris@bristol.ac.uk)

ETHICS AND CONSENT
* Please provide the name(s) of the institutional review board(s) that provided ethical approval in the UK (if relevant).

Ethical approval was not sought in the UK, as data collection was not done in the UK.

* Please provide more information about the assent process through a
proxy for those with cognitive impairment. In particular, who was the proxy and how was cognitive impairment determined. Please ensure that the research complies with the PLOS policy in full: <https://journals.plos.org/plosmedicine/s/human-subjects-research#loc-patient-privacy-and-informed-consent-for-publication>

We have not clarified on the assent process using the following revised statements, on page 13:*Before data collection, all participants provided written informed consent or assent through a proxy (family member, carer, friend) for those with cognitive impairment. Cognitive impairment was implied for participants who could not understand, retain or weigh up information provided or communicate their decision based on this information.*

FIGURES

* Please provide titles and legends for all figures and tables (including those in Supporting Information files). Please define all acronyms used in each figure or table in its corresponding legend.

We have provided the titles and legends of all figures and tables

* Please ensure that where relevant figures include 95% CIs.

Where relevant, the figures have included 95% CIs

* When a p-value is given, please specify the statistical test used to determine it in the legend.

We have now included in the Tables where we report p-values: *P-values were derived from Wald z-tests.*

* Please consider avoiding the use of red and green in order to make your figure more accessible

We have avoided the use of red and green where distinctions need to be made. The use of red and green in Figure 4 does not affect the distinction identification, as each of the colours has been used in a standalone analysis.

* Please provide the unadjusted comparisons as well as the adjusted comparisons in all relevant Tables

We have provided unadjusted and adjusted comparisons in Table 2

* Please specify the variables controlled for in all relevant Tables

We have specified variables controlled for in Table 2

INCLUSIVITY IN GLOBAL RESEARCH
PLOS has a 'Inclusivity in Global Research' policy which aims to promote collaboration and inclusivity in global health research. You are required to complete PLOS’ questionnaire on inclusivity in global research and submit it with your revised paper. The policy and questionnaire can be found at <https://journals.plos.org/plosone/s/best-practices-in-research-reporting>.

We have now completed the inclusivity in global research questionnaire and uploaded it with the revised paper.

OBSERVATIONAL, COHORT, CROSS-SECTIONAL, AND CASE CONTROL STUDIES

* Please ensure that the study is reported according to the STROBE guideline, and include the completed STROBE checklist as Supporting Information.  Please add the following statement, or similar, to the Methods: ""This study is reported as per the Strengthening the Reporting of Observational Studies in Epidemiology (STROBE) guideline (S1 Checklist).""
The STROBE guideline can be found here: <http://www.equator-network.org/reporting-guidelines/strobe/>

We have reported the study according to the STROBE guideline and have included the following statement, on page 8: *This study is reported using the STROBE (Strengthening the reporting of observational studies in epidemiology) checklist for cross-sectional studies (S1 Checklist) [20].*

When completing the checklist, please use section and paragraph numbers, rather than page numbers.

Thank you: we have used paragraph numbers rather than page numbers

* Did your study have a prospective protocol or analysis plan? Please state this (either way) early in the Methods section.

a) If a prospective analysis plan (from your funding proposal, IRB or other ethics committee submission, study protocol, or other planning document written before analyzing the data) was used in designing the study, please include the relevant prospectively written document with your revised manuscript as a Supporting Information file to be published alongside your study, and cite it in the Methods section. A legend for this file should be included at the end of your manuscript.

b) If no such document exists, please make sure that the Methods section transparently describes when analyses were planned, and when/why any data-driven changes to analyses took place. 

c) In either case, changes in the analysis-- including those made in response to peer review comments-- should be identified as such in the Methods section of the paper, with rationale."

We have now added the following statement regarding the planning of the analysis and data-driven changes, on page 13: *The analyses were pre-planned as part of an internship in May 2024. Data-driven changes to the analyses (emerging from health-related quality of life related analysis) were incorporated in June 2025. Changes to the analyses following peer review were incorporated between January and April 2026.*

* Your study is observational and therefore causality cannot be inferred. Please remove language that implies causality and refer to associations instead.
* For all observational studies, in the manuscript text, please indicate: (1) the specific hypotheses you intended to test, (2) the analytical methods by which you planned to test them, (3) the analyses you actually performed, and (4) when reported analyses differ from those that were planned, transparent explanations for differences that affect the reliability of the study's results. If a reported analysis was performed based on an interesting but unanticipated pattern in the data, please be clear that the analysis was data-driven.

Thank you for this suggestion, we have removed language implying causality and use association language instead. We have also added the specific hypothesis we intended to test using the following text on page 8: *Informed by literature, we hypothesised that socioeconomic (e.g. older age, high educational attainment), household (e.g. high household wealth), lifestyle (e.g. obesity) factors were associated with hypertension and diabetes[20, 21]; and untreated and uncontrolled hypertension and diabetes were associated with lower quality of life [22, 23].*

We have described the analytical methods in the Statistical analysis section, including the analyses that were performed. We have specified that although the analyses were pre-planned, some data-driven changes to the analyses were incorporated using the following statements, on page 13: *The analyses were pre-planned as part of an internship in May 2024. Data-driven changes to the analyses (emerging from health-related quality of life-related analysis) were incorporated in June 2025.*

Comments from Reviewers:

Reviewer #3: I thank the authors for addressing my remaining comments comprehensively. I have no further comments that require my re-review.

A very minor thing that the authors may want to check: in the statement "While analyses were pre-planned, the statistical analysis plan was not pre-specified", I wonder whether the authors had intended the last word to be "published" rather than "pre-specified"? If the analyses were pre-planned, then there would have been a pre-specified SAP - it's just that that was not deposited externally, I assume?

Thank you for spotting this, we have now edited the statement to read: *Whilst analyses were pre-planned, the statistical analysis plan was not published.*

Reviewer #4: I thank the authors for their responses to my comments (I was reviewer 4 in the previous round). I have no further comments.
