# Supplementary material for: Hypertension and diabetes prevalence, associated factors, care cascade, and quality of life in older adults: A cross-sectional population-based study in The Gambia, South Africa, and Zimbabwe
Source: PLoS Med. 2026 Jul 7;23(7):e1004785. doi: 10.1371/journal.pmed.1004785 (PMC13367908; doi:10.1371/journal.pmed.1004785)
Supplement: Peer review history

# Editor report: Decision Letter 0

**DOI:** 10.1371/journal.pmed.1004785.r001

**Authors:** Andreia Cunha

7 Oct 2025

Dear Dr Manyara,

Thank you for submitting your manuscript entitled "Hypertension and diabetes prevalence, associated factors, care cascade, and quality of life in older adults: cross-sectional population-based study in The Gambia, South Africa, and Zimbabwe" for consideration by PLOS Medicine.

Your manuscript has now been evaluated by the PLOS Medicine editorial staff and I am writing to let you know that we would like to send your submission out for external peer review.

However, before we can send your manuscript to reviewers, we need you to complete your submission by providing the metadata that is required for full assessment. To this end, please login to Editorial Manager where you will find the paper in the 'Submissions Needing Revisions' folder on your homepage. Please click 'Revise Submission' from the Action Links and complete all additional questions in the submission questionnaire.

For clinical studies, please upload a copy of your trial study protocol as a supporting information file. The study protocol should be the version submitted for approval to the institutional review board or ethics committee, should include any amendments to the study protocol, as well as the date of their approval by the institutional review or ethics committee. Please also detail any deviations from the study protocol in the Methods section of your manuscript. The editors will consider the protocol and study conduct prior to a final decision for external review.

Please re-submit your manuscript within two working days, i.e. by Oct 09 2025 11:59PM.

Login to Editorial Manager here: https://www.editorialmanager.com/pmedicine

Once your full submission is complete, your paper will undergo a series of checks in preparation for peer review. Once your manuscript has passed all checks it will be sent out for review.

Feel free to email us at plosmedicine@plos.org if you have any queries relating to your submission.

Kind regards,

Andreia Cunha, PhD

Senior Editor

PLOS Medicine

# Peer review: Decision Letter 1

**DOI:** 10.1371/journal.pmed.1004785.r002

**Authors:** Andreia Cunha

19 Dec 2025

Dear Dr Manyara,

Apologies for the delay in getting back to you with a decision, which was due to challenges in securing all the necessary advice. Many thanks for submitting your manuscript "Hypertension and diabetes prevalence, associated factors, care cascade, and quality of life in older adults: cross-sectional population-based study in The Gambia, South Africa, and Zimbabwe" (PMEDICINE-D-25-03448R1) to PLOS Medicine. The paper has been reviewed by subject experts and a statistician; their comments are included below and can also be accessed here: [LINK]

As you will see, the reviewers find your work of interest but they raise important questions and concerns that we must ask you to address in full. After discussing the paper with the editorial team, I'm pleased to invite you to revise the paper in response to the reviewers' comments. We plan to send the revised paper to some or all of the original reviewers, and we cannot provide any guarantees at this stage regarding publication.

In addition to these revisions, you may need to complete some formatting changes, which you will receive in a follow up email. A member of our team will be in touch with a set of requests shortly. If you do not receive a separate email within a few days, please assume that checks have been completed, and no additional changes are required.

When you upload your revision, please include a point-by-point response that addresses all of the reviewer and editorial points, indicating the changes made in the manuscript and either an excerpt of the revised text or the location (eg: page and line number) where each change can be found. Please also be sure to check the general editorial comments at the end of this letter and include these in your point-by-point response. When you resubmit your paper, please include a clean version of the paper as the main article file and a version with changes tracked as a marked-up manuscript. It may also be helpful to check the guidelines for revised papers at http://journals.plos.org/plosmedicine/s/revising-your-manuscript for any that apply to your paper.

We ask that you submit your revision by Jan 30 2026 11:59PM. However, if this deadline is not feasible, please contact me by email, and we can discuss a suitable alternative.

Don't hesitate to contact me directly with any questions (acunha@plos.org).

Best regards,

Andreia

Andreia Cunha, PhD

Senior editor

PLOS Medicine

acunha@plos.org

-----------------------------------------------------------

Comments from the reviewers:

Reviewer #1: This manuscript reports a well-conducted, cross-sectional survey assessing prevalence, risk factors, care cascade stages, and health-related quality of life (HRQoL) for hypertension and diabetes across rural and urban sites in three countries. The study addresses an important public health topic and provides valuable comparative data.

The study is methodologically sound and addresses an important topic. With improved contextualization, clearer presentation of results, and deeper discussion of cross-country differences, the manuscript could make a significant contribution to the literature on NCD care in sub-Saharan Africa.

Major Points:

1.Introduction and Context: The introduction is clear, but it omits some recent studies and reviews on hypertension and diabetes prevalence in Africa. Including or referencing these would help contextualize the study and clarify its added value.

2.Study Design and Implementation: Data were collected over nearly two years and embedded within a vertebral fracture study. Additional discussion of potential implications of this design (e.g., timing effects, site comparability) would improve interpretation.

3.Results Presentation: Some descriptive results, particularly for prevalence and HRQoL, are dense and difficult to follow. Simplifying sentence structure, standardizing presentation (country, value, confidence interval), and minimizing repetition would improve clarity. Adding a few interpretive comments in the results could help guide readers.

4.Discussion and Interpretation: While findings align with existing literature, the manuscript could better highlight its unique contribution. Differences across countries could be discussed in the context of local health systems, policies, and capacities, which would strengthen the policy relevance of the study.

Reviewer #2: The authors should be commended for this extensive work aimed at characterizing the burden of hypertension and diabetes mellitus in three African countries. In the main, the prevalence of hypertension and diabetes mellitus remains high and control rates are poor. Authors should address the following comments:

1.QoL values should be expressed as percentages up to 1 decimal place.

2.No Y-axis label for Figure 2

3.No Y-axis label for Figure 3

4.Table H: Metformin belongs to the Biguanides

Reviewer #3: SUMMARY

The manuscript presents a three-country population-based study (The Gambia, South Africa and Zimbabwe) investigating hypertension and diabetes prevalence and care cascade in adults over 40 years of age. Using a cross-sectional design across 3 countries, 5 settings (urban and rural settings in each country, except for rural Zimbabwe), the authors estimate the prevalence of hypertension and prevalence, the proportion of cases dropping out at different stages of the care cascade and investigate risk factors associated both with each condition but also each step in the care cascade. Further, they evaluate quality of life at different steps in the care cascade.

The authors found high prevalence in the studied populations for both conditions, in both rural and urban settings, and generally poor progression along the care cascade (but worse for diabetes), though this is less pronounced in South Africa.

PURPOSE OF REVIEW

This is a statistical review and focuses foremost on statistical rigour of the study, though I do add some less statistical comments.

OVERALL ASSESSMENT

This is an important study generating results / data on hypertension and diabetes (prevalence, risk factors, care cascade) where there are scant data and should be published.

The study design is largely appropriate, though perhaps unclear why specifically The Gambia, South Africa and Zimbabwe were chosen. Taken together these results will not be representative of the wider sub-Saharan African landscape (though the authors do not claim this anyway), particularly because of the absence of Eastern Africa.

I do have some reservations about some of the analytical approaches used by the authors, however. The analysis is largely descriptive, and the descriptive analyses are, for the most part, all robust and appropriate. The one exception here are descriptive results for the pooled data across all countries as this is epidemiologically somewhat dubious as the five settings are quite distinct and the overall results depend on the relative sample sizes for each setting (see comment M2 below). More importantly, however, there is a very odd analytical choice to use aggregate-level meta-analysis for the association analyses for hypertension and diabetes, and this may need review (see comment M2 below). Further, there are absolutely no details provided in Methods about what specific meta-analysis model was used and so it is in general to assess how valid or not these analyses are (see comment M2 below). For care cascade analyses, GAM models are used but again details of these models are lacking (see comment M4). It is also unclear whether the statistical analysis followed a pre-specified SAP. Taken together, there is a need for the manuscript to be revised with a much more detailed description of the statistical analysis and a justification for the main analytical choices.

As a study presenting observational data, the authors rightly follow STROBE guidelines for reporting results and helpfully appended a completed checklist. There are, however, minor issues with STROBE compliance (see below for specifics). Generally, on presentation of results, the main issue is the sheer abundance of results the study generated. For this reason, it seems the authors tried to focus on the most relevant results, at the risk of selective reporting - I feel that results on prevalence and care cascade could be presented more clearly with consistent reporting across conditions, care cascade steps and countries: e.g. i) overall results, ii) results per country , iii) results per age group, iv) results per country & age group, v) care cascade results consistently according to the 80-80-80, resp. 80-80-80-60, targets (see comment M5).

There are also occasional mistakes in some of the numbers reported (some examples in the detailed comments below), so there may be a need for the authors to carefully check all numbers for accuracy.

MAJOR COMMENTS

M1. STROBE guideline deviations / necessary clarifications:

* Item 7 is OK, but details on which variables are considered exposures, confounders, mediators are listed only later on p.13/14.

* Items 12c, 12d, and 12e are not "N/A" and should be completed, even if just to state that data were complete (which numbers in Table 1 confirm is not the case), that the sampling strategy was ignored (as is the case) and that no sensitivity analyses were done. The latter may not be appropriate depending on what the authors did regarding missing data (which currently is not known from what is included in the manuscript). The aggregate-level meta-analysis (an odd analytical choice - see also comment M2) could also have been subjected to a sensitivity analysis to confirm results.

* Item 13a: Figure A should ideally be in the main body of the manuscript. Figure A suggests there may have been additional eligibility criteria (e.g. disability, frailty, ...) - can the authors clarify this? further Figure A implies that there were maximum recruitment targets per age-sex stratum which is not mentioned in Methods. Finally Figure A uses a different age categorisation than the rest of the manuscript, which is confusing.

* Item 14b: Table 1 does NOT list missing data proportions as claimed on the completed checklist. It only does so implicitly, and it is up to the reader to check for which variables there was how much missingness. There clearly were missing data as not all variables sum to the total number of records. There is also at least one small mistake in Table 1: 190/1052 = 18% not 17%.

* Item 15: consider putting this in a single table for easier reference (e.g. similar to Tables A-F in the Appendix S1 but with counts) and include it in the main body of the manuscript.

* Item 16b: This is not "N/A" and is given on p.11-12.

* Item 18: This spans more than just p.32.

* Item 19: There are some missing limitations in the discussion section - e.g. no mention is made of the restriction to 3 countries only which limits generalisability beyond these 3 countries, or the fact that the HRQoL value set for all countries was the one from Zimbabwe - not optimal for The Gambia or South Africa.

M2. The analysis largely presents descriptive statistics and basic association analyses stratified by country. However, there are cross-country analyses. Generally, the "overall" results are difficult to interpret as this depends on the relative sizes of the participant sets in the different countries and the split between urban / rural -- not clear what this "overall" population is representative of. This is however OK as it is clear what was done - the implication is mostly on interpretation but remains epidemiologically a bit dubious. Fairly puzzling, however, is the choice to use an aggregate-level meta-analysis (specific details of the meta-analysis model etc are missing from the manuscript - so difficult to be sure of details) for association analyses across countries. The only thing that is clear is that the meta-analysis must have been aggregate given that the authors state that there was no need to account for urban/rural setting as this was collinear with site. Given that the authors had the individual-level data from each country, it is unclear why not simply a pooled analysis accounting for country was done (this would be equivalent to an IPD meta-analysis), which is the more obvious analytical choice and would have had better statistical properties.

M3. The sampling involves sampling blocks for each site, then inviting everyone eligible within the blocks to participate in the study (until targets achieved). The analysis does not reflect this sampling strategy other than that some analyses are stratified by site. Did the authors account for block in any of the analyses and did this change any of the results substantially?

M4. The details on the GAMs used to interrogate associations between HRQoL and care cascade steps are missing - e.g. what smooth(s) with what parameters were used for which variables (given that the outcome in these models is HRQoL, most explanatory variables would be categorical - or was HRQoL the predictor not the outcome as stated on p.14)?

M5. There are 5 sites, 3 care cascade steps in hypertension and 4 in diabetes. This means there are potentially a lot of results to report for the case cascade. As such, the section on this, starting on p.22, probably has to be somewhat selective in what it reports. However, I found it very difficult to read as I failed to see a clear consistent way how this is reported other than focusing on statistically significant associations. I wonder if consistently reporting according to 80-80-80 (hypertension) and 80-80-80-60 (diabetes) would be easier to report. Also switching between percentage achieved and percentage missing (the gap) contributes to making this less straightforward to follow. As an example, overall control is reported consistently for hypertension and diabetes and both conditions combined but then switches to stating numbers/ percentages that dropped out at specific steps rather than reporting how many successfully completed each step which would directly speak to the targets. Also, for diabetes and both conditions combined, not all reasons for dropping out are stated unlike for hypertension. E.g. for diabetes, report overall control 21.9% (161/375), with 50.3% (370/735) of hypertensive participants diagnosed (gap with target: 29.7%), xx% (xx/735) treated, gap xx%, xx% (xx/735) controlled, gap xx%, and xx% (xx/xx) on statins, gap xx%.

SPECIFIC POINTS

S1. Abstract / p.2: Is the definition of minimally important difference of 1 SD standard practice or why was this chosen?

S2. Abstract / p.3: As an example of the somewhat selective reporting (though aware this is the abstract and there is not that much space), the association between treated and controlled diabetes and HRQoL is only reported for The Gambia and Zimbabwe and South Africa (where this was not statistically significant) is not mentioned.

S3. Overall, the results for HRQoL and the care cascade seem to come a bit short in the abstract given that this was a key objective (e.g. see 3rd bullet in the author summary).

S4. If most existing data have been on <50-year-olds, why was this study not restricted to >= 50 years of age?

S6. P.6, last paragraph: What is the exact difference between "untested" and "undiagnosed" - presumably the latter is untested diseased individuals whereas the former also includes healthy untested individuals? The rest of the manuscript does not really make a difference here (or only focuses on undiagnosed as opposed to untested).

S7. P.6, last paragraph: The second sentence mentions 12 countries, states 2 percentages and lists 4 specific countries - so unclear for which countries the 80% and 62% refer to.

S8. The rationale given on p.7 for doing this study in 3 countries only needs to be clearer. E.g. on p.6, 12 countries were mentioned, and unclear why no country from Eastern Africa was included and why 2 countries from Southern Africa were included for example.

S9. P.8, sample size calculation: the statement regarding >90% power for detecting an OR of 2.0 or larger is not in correct; but in fact this is powered to detect on odds ratio as low as 1.74 with 90% power (the power for OR 2.0 is 98.6%).

S10. P.11, first paragraph: Was there no other management of those with low blood glucose other than giving them a sugary snack?

S11. P.11, third paragraph: The binarisation of some variables is statistically not optimal as information is lost, power for detection associations is reduced and results will depend on the specific cut-off chosen. Did the authors do any sensitivity analyses to check the effect of changing the binary cut-off? A similar point pertains to the categorisation of continuous variables such as BMI on p.12.

S12. P.12, Quality of Life: If I read correctly, then the same value set for Zimbabwe was also used for The Gambia and South Africa? These are quite distinct populations and I would not be surprised if their value sets differed. I understand the pragmatic choice if a value set was only available for Zimbabwe, but was any sensitivity analysis done to check how much results would differ if other value sets from SSA would have been used for The Gambia and South Africa?

S13. Statistical analysis section: Please specify whether there was a pre-specified Statistical Analysis Plan that was followed or not. If yes, where is this deposited / published? Were there any deviations from it?

S14. It is not incorrect to use a logistic regression model to estimate prevalence with corresponding 95% CI, but it would be more typical to compute exact binomial confidence intervals using the Clopper-Pearson approach. Why was the logistic regression approach chosen (which makes perfect sense for the association analyses, but is just a bit surprising for using it for estimation and yields point estimates that are not as intuitive as k/n)?

S15. Table 1: Add a count and % of missing data for each variable (or respectively the number and percentage of records for which each variable was available). It is clear that not all variables have complete records, e.g. for rural The Gambia, the numbers for educational attainment and BMI categories only sum to 1,045 not 1,052. Please also carefully check all numbers in this table; there is at least one error (e.g. tobacco use for rural The Gambia: even if all participants had this recorded, the percentage would be at least 190/1052 which is 18% not 17%).

S16. Figure 1: Please add also the actual counts on top of each bar, in addition to the percentage which is already shown.

S17. P.19, 2nd sentence ("The diabetes prevalence ..."): This statement is only correct in so far as a binary interpretation of statistical significance is made. In all settings the diabetes prevalence is higher in the older age group, even if only statistically significantly so in urban South Africa.

S18. Figure 4: This is a really nice, clear figure. While I am not sure that 'overall' is a very meaningful analysis population, but given how often it is being referred to in the text, it might be clearer if a bar for 'overall' is added to this figure. Also add counts on top of each bar (together with the percentages which are already shown) and totals (maybe at the bottom?).

S19. Figure 5: 1) Unclear how meaningful the broken line / median is given that this is both across sites and conditions. I also though the fact that HRQoL for non-hypertensive and undiagnosed are broadly similar was quite interesting and would be worth commenting on.

S20. P.30: Comparing controlled & treated to non-hypotensive / non-diabetic participants is appropriate. But I think this section could be improved by also commenting on comparisons between each failure in the case cascade and being treated and controlled - this is where improvements could be made by improving achieved levels on the care cascade pathway; i.e. add comparisons of undiagnosed vs treated & controlled, untreated vs treated & controlled and treated but uncontrolled vs treated & controlled.

S21. P.32: The sentence "Undiagnosed disease contributed most ..." needs rewriting. As written it seems to be at odds with Figure 4 for hypertension where the diagnostic gap is not that large (and control is much worse in both conditions).

S22. P.34: For easier reading of this, it would help if the targets are restated somewhere on this page. Please also check stated percentages, e.g. the 29% on line 5 on this page seems to be 30% if properly rounded from the numbers on p.24 (735-365 = 370 diagnosed --> 50.3% --> gap of 29.7% to get to 80%), and the 20% from line 9 seems to be 25.4%, based on the numbers from p.22 (I get 913 controlled out of 1679 treated; i.e. 54.4% --> gap of 25.6% to get to 80% target).

S23. P.35, last line: Causal inference is only precluded up to a point -- there are causal modelling techniques for observational, cross-sectional data (but of course these come at a cost and have limitations)...

TYPOS & MINOR POINTS

T1. Abstract / p.3: "adults in The Gambia" reads a bit strangely as all participants were adults; I presume the authors meant "all participants in The Gambia"?

T2. P.8, second paragraph: replace "to detect, with 2.5% precision" with "to estimate, with 2.5% precision"; detection suggests some type of null hypothesis testing, but this is clearly about estimation not testing. Then add "to detect" in front of "an odds ratio of 2.0" in the next line.

T3. P.8, second paragraph: What has got "vertebral fracture" got to do with hypertension or diabetes?

T4. Last line of p.10 and first two lines of p.11: This should be in the Results section.

T5. P.15, last paragraph: "food insecurity was so in South Africa" --> "food insecurity was most common in South Africa"

T6. P.17, last sentence before "Factors associated with ...": It is not clear what the second half of this sentence is saying - can you rephrase?

T7. Table 2: Continuous colour legend scales might be clearer than just giving 2 levels for each scale. Consider also using different colours for protective ORs <1. What I^2 indicates is unclear as not described in Methods or Results - presumably this is the proportion of variation unexplained by sampling variation?

T8. P.27: Something missing in the last sentence - what does 'of SDH...' and 'of IDH...' refer to?

T9. P.34: "...as low household wealth.." --> "...low household wealth..."

T10. P.34: "on a statin" --> "on statins"

T11. P.35, fifth/sixth line: "as it is influenced" --> "as they are influenced"

T12. P.35: better / lower quality of life - compared to what control? Healthy or undiagnosed but diseased or diagnosed and untreated, ...?

T13. P.36, sixth line: "a single random blood glucose measurement" -- should this be "a single elevated random blood glucose measurement"

T14. P.36: "some, though very few" --> "some, most likely very few" (given that this is conjecture, albeit very reasonable, I think this should be expressed more cautiously)

T15. P.36: "Despite these limitations, [...]" I think this should be on a new paragraph.

Reviewer #4: This paper used cross-sectional data from urban and rural sites in three African countries to estimate the prevalence of hypertension and diabetes, their risk factors, care continuum outcomes, medication use, and association with quality of life. Overall, the paper covers an important topic with a rare and valuable source of data. I provide my specific suggestions for improving the paper below.

Motivation and Focus

The authors present a large number of analyses with varying degrees of motivation. This makes it hard to parse all the paper's findings and the central contribution of the paper becomes a bit lost. My suggestion would be to focus on a smaller subset of analyses that are more strongly and convincingly motivated in the introduction. Specifically:

1. For the prevalence and care continuum analyses, the authors need to directly confront what their paper brings above the STEPS and DHS data sources. I am not immediately familiar with whether their countries have STEPS/DHS data. If not, the authors should state this directly as a clear motivation; if yes, it would be very important that the authors then argue for what their analyses bring above these existing sources of data.

2. I found the hypertension subtypes and association of continuum outcomes with quality of life to be less well motivated. Beyond stating that these analyses don't exist for their countries, the authors don't convince the reader of why these are important to know. My suggestion would be to move these results to an appendix rather than focus on them in the main results as there is already quite a lot going on in the main paper.

In general, I felt the introduction could be significantly improved to be clearer about the main contributions of the paper.

Data and Sampling

Overall, I was impressed with the quality of data and the high response rates. The big open question I was left with was:

1. Are the data sub-nationally representative (e.g. do they represent the rural and urban populations of their countries), or, are among a selected population? The authors should provide more information on this in the main paper (rather than refer readers to an external protocol) as it has direct relevance for how widely applicable the findings are.

Analyses and Results

1. As stated above, I found the analyses of hypertension subtypes and the association of continuum outcomes with quality of life to be not as well motivated or immediately relevant. I would suggest the authors move these to an appendix to bring more clarity to the results section.

2. Given the large number of results, I would suggest that the authors present the results pooled over age (rather than stratified by age group) in their main results, and discuss age differences in an Appendix. I found it hard to make sense of all the results given the number of stratifications.

3. I may have missed it, but the authors mention that they will meta-analyze their results in the methods section but I did not see those corresponding results in the text.

4. The data on medications is rich and rare and I think the authors could exploit this more. Specifically, I was wondering if the number/type of medications was associated with control among those that report any treatment. Right now the authors briefly state that number of medications was not associated with control but I was left wondering which population this was among. This medication data could go a long way to understanding why there is such poor control among the treated (e.g is it adherence or under-dosed medications?)

5. A minor point: given the high prevalence of both hypertension and diabetes, I would suggest that the authors present their regression results as risk ratios or risk differences, as the odds ratio is less appropriate here and is harder to interpret.

Any attachments provided with reviews can be seen via the following link: [LINK]

--------------------------------------------------------- ---

General editorial requests:

(Note: not all will apply to your paper, but please check each item carefully)

*We ask every co-author listed on the manuscript to fill in a contributing author statement, making sure to declare all competing interests. If any of the co-authors have not filled in the statement, we will remind them to do so when the paper is revised. If all statements are not completed in a timely fashion this could hold up the re-review process. If new competing interests are declared later in the revision process, this may also hold up the submission. Should there be a problem getting one of your co-authors to fill in a statement we will be in contact. Please do not add or remove authors without first discussing this with the handling editor. You can see our competing interests policy here: http://journals.plos.org/plosmedicine/s/competing-interests.

*Please upload any figures associated with your paper as individual TIF or EPS files with 300dpi resolution at resubmission; please read our figure guidelines for more information on our requirements: http://journals.plos.org/plosmedicine/s/figures. While revising your submission, we strongly recommend that you use PLOS's NAAS tool (https://ngplosjournals.pagemajik.ai/artanalysis) to test your figure files. NAAS can convert your figure files to the TIFF file type and meet basic requirements (such as print size, resolution), or provide you with a report on issues that do not meet our requirements and that NAAS cannot fix.

After uploading your figures to PLOS's NAAS tool - https://ngplosjournals.pagemajik.ai/artanalysis, NAAS will process the files provided and display the results in the "Uploaded Files" section of the page as the processing is complete.

If the uploaded figures meet our requirements (or NAAS is able to fix the files to meet our requirements), the figure will be marked as "fixed" above. If NAAS is unable to fix the files, a red "failed" label will appear above.

When NAAS has confirmed that the figure files meet our requirements, please download the file via the download option, and include these NAAS processed figure files when submitting your revised manuscript.

*Please ensure that the paper adheres to the PLOS Data Availability Policy (see http://journals.plos.org/plosmedicine/s/data-availability), which requires that all data underlying the study's findings be provided in a repository or as Supporting Information. For data residing with a third party, authors are required to provide instructions with contact information (web or email address) for obtaining the data. Please note that a study author cannot be the contact person for the data. PLOS journals do not allow statements supported by "data not shown" or "unpublished results." For such statements, authors must provide supporting data or cite public sources that include it.

*We expect all researchers with submissions to PLOS in which author-generated code underpins the findings in the manuscript to make all author-generated code available without restrictions upon publication of the work. In cases where code is central to the manuscript, we may require the code to be made available as a condition of publication. Authors are responsible for ensuring that the code is reusable and well documented. Please make any custom code available, either as part of your data deposition or as a supplementary file. Please add a sentence to your data availability statement regarding any code used in the study, e.g. "The code used in the analysis is available from Github [URL] and archived in Zenodo [DOI link]" Please review our guidelines at https://journals.plos.org/plosmedicine/s/materials-software-and-code-sharing and ensure that your code is shared in a way that follows best practice and facilitates reproducibility and reuse. Because Github depositions can be readily changed or deleted, we encourage you to make a permanent DOI'd copy (e.g. in Zenodo) and provide the URL.

FORMATTING - GENERAL

*Abstract: Please structure your abstract using the PLOS Medicine headings (Background, Methods and Findings, Conclusions). Please combine the Methods and Findings sections into one section.

*At this stage, we ask that you include a short, non-technical Author Summary of your research to make findings accessible to a wide audience that includes both scientists and non-scientists. The Author Summary should immediately follow the Abstract in your revised manuscript. This text is subject to editorial change and should be distinct from the scientific abstract. Ideally each sub-heading should contain 2-3 single sentence, concise bullet points containing the most salient points from your study. In the final bullet point of 'What Do These Findings Mean?', please include the main limitations of the study in non-technical language. Please see our author guidelines for more information: https://journals.plos.org/plosmedicine/s/revising-your-manuscript#loc-author-summary.

*Please express the main results with 95% CIs as well as p values. When reporting p values please report as p<0.001 and where higher as the exact p value p=0.002, for example. Throughout, suggest reporting statistical information as follows to improve clarity for the reader "22% (95% CI [13%,28%]; p</=)". Please be sure to define all numerical values at first use.

*Please include page numbers and line numbers in the manuscript file. Use continuous line numbers (do not restart the numbering on each page).

*Please cite the reference numbers in square brackets. Citations should precede punctuation.

FIGURES AND TABLES

*Please provide titles and legends for all figures and tables (including those in Supporting Information files).

*Please define all abbreviations used in each figure/table (including those in Supporting Information files).

*Please consider avoiding the use of red and green in order to make your figure more accessible to those with color blindness.

SUPPLEMENTARY MATERIAL

*Please note that supplementary material will be posted as supplied by the authors. Therefore, please amend it according to the relevant comments outlined here.

*Please cite your Supporting Information as outlined here: https://journals.plos.org/plosmedicine/s/supporting-information

REFERENCES

*PLOS uses the numbered citation (citation-sequence) method and first six authors, et al.

*Please ensure that journal name abbreviations match those found in the National Center for Biotechnology Information (NCBI) databases (http://www.ncbi.nlm.nih.gov/nlmcatalog/journals), and are appropriately formatted and capitalised.

*Where website addresses are cited, please include the complete URL and specify the date of access (e.g. [accessed: 12/06/2023]).

*Please also see https://journals.plos.org/plosmedicine/s/submission-guidelines#loc-references for further details on reference formatting.

OBSERVATIONAL STUDIES

*Abstract: Please include the study design, population and setting, number of participants, years during which the study took place (enrollment and follow up), length of follow up, and main outcome measures.

*Please ensure that the study is reported according to the STROBE (or appropriate STOBE extension) guideline (available from: https://www.equator-network.org/reporting-guidelines/strobe) and include the completed STROBE (or STROBE extension) checklist as Supporting Information. Please add the following statement, or similar, to the Methods: "This study is reported as per the Strengthening the Reporting of Observational Studies in Epidemiology (STROBE) guideline (S1 Checklist)." When completing the checklist, please use section and paragraph numbers, rather than page numbers.

*[FOR POPULATION HEALTH/REGISTRY STUDIES] Please ensure that the study is reported according to the RECORD guideline (available from https://www.record-statement.org) and include the completed checklist as Supporting Information. Please add the following statement, or similar, to the Methods: "This study is reported as per the Reporting of Studies Conducted using Observational Routinely-Collected Data (RECORD) guideline (S1 Checklist)." When completing the checklist, please use section and paragraph numbers, rather than page numbers.

*[FOR POPULATION HEALTH ESTIMATES] Please ensure that the study is reported according to the GATHER statement (available from https://www.equator-network.org/reporting-guidelines/gather-statement) and include the completed checklist as Supporting Information. Please add the following statement, or similar, to the Methods: "This study is reported as per the Guidelines for Accurate and Transparent Health Estimates Reporting (GATHER) statement (S1 Checklist)." When completing the checklist, please use section and paragraph numbers, rather than page numbers.

*[FOR MEDIATION ANALYSES] We recommend that the study is reported according to the AGReMA statement (https://agrema-statement.org/#:~:text=AGReMA%20is%20an%20evidence%2D%20and,randomised%20trials%20and%20observational%20studies) and include the completed checklist as Supporting Information. Please add the following statement, or similar, to the Methods: "This study is reported as per the Guideline for Reporting Mediation Analyses (AGReMA) statement (S1 Checklist)." When completing the checklist, please use section and paragraph numbers, rather than page numbers.

*For all observational studies, in the manuscript text, please indicate: (1) the specific hypotheses you intended to test, (2) the analytical methods by which you planned to test them, (3) the analyses you actually performed, and (4) when reported analyses differ from those that were planned, transparent explanations for differences that affect the reliability of the study's results. If a reported analysis was performed based on an interesting but unanticipated pattern in the data, please be clear that the analysis was data driven.

*Please state in the Methods section whether the study had a prospective protocol or analysis plan. If a prospective analysis plan (from your funding proposal, IRB or other ethics committee submission, study protocol, or other planning document written before analyzing the data) was used in designing the study, please include the relevant document(s) with your revised manuscript as a Supporting Information file to be published alongside your study and cite it in the Methods section. A legend for this file should be included at the end of your manuscript. If no such document exists, please make sure that the Methods section transparently describes when analyses were planned, and when/why any data-driven changes to analyses took place. Changes in the analysis, including those made in response to peer review comments, should be identified as such in the Methods section of the paper, with rationale.

# Author comment: Author response to Decision Letter 2

**DOI:** 10.1371/journal.pmed.1004785.r003

2 Feb 2026

Attachment Submitted filename: Peer reviwew response.docx

# Peer review: Decision Letter 2

**DOI:** 10.1371/journal.pmed.1004785.r004

**Authors:** Andreia Cunha

11 Mar 2026

Dear Dr Manyara,

Sincere apologies for the delay in getting back to you with a decision, which was due to challenges in securing the necessary advice. Many thanks for submitting your manuscript "Hypertension and diabetes prevalence, associated factors, care cascade, and quality of life in older adults: cross-sectional population-based study in The Gambia, South Africa, and Zimbabwe" (PMEDICINE-D-25-03448R2) to PLOS Medicine. The paper has been reviewed by three of the original Reviewers, including a statistician; their comments are included below and can also be accessed here: [LINK]

As you will see, Reviewers 3 and 4 still have some remaining points that we must ask you to address in full. After discussing the paper with the editorial team and an academic editor with relevant expertise, I'm pleased to invite you to revise the paper in response to the reviewers' comments. We plan to send the revised paper to some or all of the original reviewers, and we cannot provide any guarantees at this stage regarding publication.

In addition to these revisions, you may need to complete some formatting changes, which you will receive in a follow up email. A member of our team will be in touch with a set of requests shortly. If you do not receive a separate email within a few days, please assume that checks have been completed, and no additional changes are required.

When you upload your revision, please include a point-by-point response that addresses all of the reviewer and editorial points, indicating the changes made in the manuscript and either an excerpt of the revised text or the location (eg: page and line number) where each change can be found. Please also be sure to check the general editorial comments at the end of this letter and include these in your point-by-point response. When you resubmit your paper, please include a clean version of the paper as the main article file and a version with changes tracked as a marked-up manuscript. It may also be helpful to check the guidelines for revised papers at http://journals.plos.org/plosmedicine/s/revising-your-manuscript for any that apply to your paper.

We ask that you submit your revision by Apr 01 2026 11:59PM. However, if this deadline is not feasible, please contact me by email, and we can discuss a suitable alternative.

Don't hesitate to contact me directly with any questions (acunha@plos.org).

Best regards,

Andreia

Andreia Cunha, PhD

Senior editor

PLOS Medicine

acunha@plos.org

-----------------------------------------------------------

Comments from the reviewers:

Reviewer #2: The authors have addressed all my comments excellently.

Reviewer #3: I thank the authors for addressing many of my somewhat long list of comments from the previous round of review - the manuscript is much improved already. I still think this is an important and generally high-quality study that should be published. Nevertheless, I remain with some reservations that I think should be addressed before publication:

Major comments:

1. My methodological comment on the meta-analysis approach remains. The authors have somewhat clarified the meta-analysis method used. It remains an aggregate-level meta-analysis, and is what is sometimes called a two-stage IPD meta-analysis. However, a one-stage IPD meta-analysis, which is achieved by using mixed effects models, is the gold standard in the literature when there is a limited number of studies / sites to be meta-analysed. I appreciate the paper cited by the authors, but I would have the same reservations for their analytical choice (I also note they had a much larger number of sites). The one-stage meta-analysis should essentially be what the authors have now included as a sensitivity analysis (though the full details of this model are not clearly enough described for me to be sure; though the text on l.326-327 does suggest a simple adjustment for country rather than treating site as a random factor was made - if so then this is not the IPD meta-analysis I suggested doing). To be clear: a one-stage, individual participant data analysis, treating site as a random factor in a mixed effects model, is a meta-analysis. The authors seem to have misinterpreted my comment as recommending to not do a meta-analysis. That was not what I was recommending, I just do not understand why the authors decided to use a less powerful approach to doing the meta-analysis than what is considered the gold standard meta-analysis - the data the authors have available would allow such a gold standard analysis. If the sensitivity analysis is this one-stage IPD meta-analysis, then I would suggest listing this as the primary analysis rather than the statistically less efficient two-stage approach where the meta-analysis component uses only aggregate data (and has a sample size of effectively just 5).

To be very clear hear: the two-stage IPD meta-analysis approach currently used in the paper is not incorrect, it is just not what is currently considered to be the best approach. I do not expect there to be major differences in results, and I will not insist on this point if the authors absolutely want to stick with the current approach - I am just trying to nudge the authors to revise their analysis so as to use a slightly better approach.

2. My comment as to the sampling strategy being ignored related to the analysis not accounting for it rather than it not being described in the manuscript (which it is). Many of the reported analyses, as described currently at least, do not account for the stratified (by site, sex and age) or the block-randomised sampling process that was used to recruit participants into the study. Obviously, some analyses are stratified and/or account for sex and age and that is fine. The sampling strategy is described in the paper, it just does not seem to have been taken into account for all analyses. For many of the descriptive analyses this is fine, but for analyses using regressions, ideally the design factors should have been accounted for. As such, I disagree with the authors' response to my original comment M3. A principled analysis would account for the blocks used in the random block sampling - effectively this introduces clustering which means that the assumption of independent data observations that virtually all standard statistical analyses make is not met and hence inference would technically not be valid. I do not expect that this would make a huge difference, but it should technically be accounted for. Related to this item 12d from the STROBE checklist should not be left as N/A.

To be very clear on this point: not accounting for the blocks is technically not correct. I do not expect that there is any strong cluster effect and hence do not expect that accounting for the blocks would make much of a difference to results. There is however a risk of underestimated standard errors and too low p-values etc here. So I urge the authors to account for this in their analyses as otherwise these would not be technically valid. If this is not possible due to the block information not having been recorded (but surely it must have given that it was required for sampling?), then this should be clearly stated.

Other comments:

3. The authors did not respond to my comment that the "overall" analysis is epidemiologically a bit dubious as this would not be reflective of any particular population given the dependence on the relative size of recruitment from each site etc. I do not suggest this should be removed from the paper and it is clearly described, but I think somewhere this caveat should be stated clearly - perhaps in the limitations in the discussion? This is related though distinct from the lack of generalisability of results to the wider SSA region given the lack of Eastern and Central African sites; but could possibly be mentioned in the same limitation section.

4. Regarding the description of models fitted as generalised additive models (GAMs) on lines 337 to 346, and given the explanation in the author's rebuttal letter that none of these models had continuous predictor variables, this means that the GAMs were just simple generalised linear models (GLMs) and should be described as such. It is not incorrect to call them GAMs, given that GAMs are extending GLMs, but it is a bit misleading to refer to a more complex model that is specifically designed to allow flexible modelling of continuous predictors when no such predictors are in fact in the models.

5. The response to my original comment S4 seems to have been inadvertently cut off and is incomplete.

6. As response to my original comment S13, the manuscript should briefly state within the manuscript whether or not analyses followed a pre-specified SAP (it would have been better if this had been published, but if there was a SAP and it was followed this should still be stated in the manuscript; this will have implications for how readers interpret results).

7. In my original comment S23, I was not suggesting that the authors do a causal analysis, it is just that the statement that a causal analysis is not possible due to this being a cross-sectional study is not correct; this could be done if this had been the intention of the authors. So, I still think the statement on what is now l.625-626 should be reworded to clarify that not doing a causal analysis was an analytical choice rather than an inevitability due to the study design. There are valid reasons for this choice: causal inference using observational data can be challenging, is often unconvincing due to strong assumptions and often not the point of the study objectives anyway.

8. I did not earlier appreciate, on what is now Figure 1, the 44 "additional eligible attendees" participants listed in the bottom section. Where did these come from and why were these not screened and invited like other participants?

Reviewer #4: I thank the authors for taking my comments seriously. I believe the revised manuscript is a significant improvement. I had a few remaining minor comments:

1. Overall, the revised introduction is much improved and clearer. The authors introduced new text based on my comment to motivate the analyses with health-related quality of life. This new text is good, however, it is placed in the middle of a paragraph, which interrupts a discussion of other data gap and breaks up an otherwise clear thought. I would suggest that the authors move this new text to a small paragraph following paragraph 3 in the intro to keep the ideas together.

2. Lines 334-336 still reference the now removed subtype analysis. This was probably just a minor typo, but it should be removed.

3. When describing the association, odds ratio, results, the authors should clearly state what the reference groups are. This information is in the table but I found myself asking "greater among those aged 55-69 compared to whom?"

4. I had two comments on the regression tables: (1) I personally did not understand the value of the shading, and this actually confused me as I initially assumed it was relating to the size of the p-value; (2) what is the value of showing model 1,2, and 3? My suggestion would be to just show the fully adjusted model 3 and put the others in the appendix if needed. This feels more intellectually robust while also streamlining the text and interpretation.

5. Lines 448-458: As the authors are now presenting the medication results in another paper, I wondered if this paragraph should be removed and saved for that paper. At a minimum, there should be mention of this analysis in the methods section.

Any attachments provided with reviews can be seen via the following link: [LINK]

--------------------------------------------------------- ---

General editorial requests:

* We ask every co-author listed on the manuscript to fill in a contributing author statement, making sure to declare all competing interests. If any of the co-authors have not filled in the statement, we will remind them to do so when the paper is revised. If all statements are not completed in a timely fashion this could hold up the re-review process. If new competing interests are declared later in the revision process, this may also hold up the submission. Should there be a problem getting one of your co-authors to fill in a statement we will be in contact. Please do not add or remove authors without first discussing this with the handling editor. You can see our competing interests policy here: http://journals.plos.org/plosmedicine/s/competing-interests.

* Please upload any figures associated with your paper as individual TIF or EPS files with 300dpi resolution at resubmission; please read our figure guidelines for more information on our requirements: http://journals.plos.org/plosmedicine/s/figures. While revising your submission, we strongly recommend that you use PLOS's NAAS tool (https://ngplosjournals.pagemajik.ai/artanalysis) to test your figure files. NAAS can convert your figure files to the TIFF file type and meet basic requirements (such as print size, resolution), or provide you with a report on issues that do not meet our requirements and that NAAS cannot fix.

After uploading your figures to PLOS's NAAS tool - https://ngplosjournals.pagemajik.ai/artanalysis, NAAS will process the files provided and display the results in the "Uploaded Files" section of the page as the processing is complete.

If the uploaded figures meet our requirements (or NAAS is able to fix the files to meet our requirements), the figure will be marked as "fixed" above. If NAAS is unable to fix the files, a red "failed" label will appear above.

When NAAS has confirmed that the figure files meet our requirements, please download the file via the download option, and include these NAAS processed figure files when submitting your revised manuscript.

*PLOS Medicine requires that the de-identified data underlying the specific results in a published article be made available, without restrictions on access, in a public repository or as Supporting Information at the time of article publication, provided it is legal and ethical to do so. Please see the policy at

http://journals.plos.org/plosmedicine/s/data-availability

and FAQs at

http://journals.plos.org/plosmedicine/s/data-availability#loc-faqs-for-data-policy

* PLOS defines the “minimal data set” to consist of the data set used to reach the conclusions drawn in the manuscript with related metadata and methods, and any additional data required to replicate the reported study findings in their entirety. Authors do not need to submit their entire data set, or the raw data collected during an investigation. Please submit the following data:

The values behind the means, standard deviations and other measures reported;

The values used to build graphs;

The points extracted from images for analysis.

* The Data Availability Statement (DAS) requires revision. For each data source used in your study:

a) If the data are freely or publicly available, note this and state the location of the data: within the paper, in Supporting Information files, or in a public repository (include the DOI or accession number).

b) If the data are owned by a third party but freely available upon request, please note this and state the owner of the data set and contact information for data requests (web or email address). Note that a study author cannot be the contact person for the data.

c) If the data are not freely available, please describe briefly the ethical, legal, or contractual restriction that prevents you from sharing it. Please also include an appropriate contact (web or email address) for inquiries (again, this cannot be a study author).

* We expect all researchers with submissions to PLOS in which author-generated code underpins the findings in the manuscript to make all author-generated code available without restrictions upon publication of the work. In cases where code is central to the manuscript, we may require the code to be made available as a condition of publication. Authors are responsible for ensuring that the code is reusable and well documented. Please make any custom code available, either as part of your data deposition or as a supplementary file. Please add a sentence to your data availability statement regarding any code used in the study, e.g. "The code used in the analysis is available from Github [URL] and archived in Zenodo [DOI link]" Please review our guidelines at https://journals.plos.org/plosmedicine/s/materials-software-and-code-sharing and ensure that your code is shared in a way that follows best practice and facilitates reproducibility and reuse. Because Github depositions can be readily changed or deleted, we encourage you to make a permanent DOI'd copy (e.g. in Zenodo) and provide the URL.

* The funding statement should include: specific grant numbers, initials of authors who received each award, URLs to sponsors’ websites. Also, please state whether any sponsors or funders (other than the named authors) played any role in study design, data collection and analysis, the decision to publish, or preparation of the manuscript. If they had no role in the research, include this sentence: “The funders had no role in study design, data collection and analysis, decision to publish, or preparation of the manuscript.”

* All authors must declare their relevant competing interests per the PLOS policy, which can be seen here: https://journals.plos.org/plosmedicine/s/competing-interests For authors with ties to industry, please indicate whether any of the interests has a financial stake in the results of the current study.

* In the ethics statement please provide more information about the process of getting assent.

FORMATTING - GENERAL

* Abstract: Please structure your abstract using the PLOS Medicine headings (Background, Methods and Findings, Conclusions). Please combine the Methods and Findings sections into one section.

* At this stage, we ask that you include a short, non-technical Author Summary of your research to make findings accessible to a wide audience that includes both scientists and non-scientists. The Author Summary should immediately follow the Abstract in your revised manuscript. This text is subject to editorial change and should be distinct from the scientific abstract. Ideally each sub-heading should contain 2-3 single sentence, concise bullet points containing the most salient points from your study. In the final bullet point of 'What Do These Findings Mean?', please include the main limitations of the study in non-technical language. Please see our author guidelines for more information: https://journals.plos.org/plosmedicine/s/revising-your-manuscript#loc-author-summary.

* Please express the main results with 95% CIs as well as p values. When reporting p values please report as p<0.001 and where higher as the exact p value p=0.002, for example. Throughout, suggest reporting statistical information as follows to improve clarity for the reader "22% (95% CI [13%,28%]; p</=)". Please be sure to define all numerical values at first use.

* Please include page numbers and line numbers in the manuscript file. Use continuous line numbers (do not restart the numbering on each page).

* Please cite the reference numbers in square brackets. Citations should precede punctuation.

FIGURES AND TABLES

* Please provide titles and legends for all figures and tables (including those in Supporting Information files).

* Please define all abbreviations used in each figure/table (including those in Supporting Information files).

* Please consider avoiding the use of red and green in order to make your figure more accessible to those with color blindness.

SUPPLEMENTARY MATERIAL

* Please note that supplementary material will be posted as supplied by the authors. Therefore, please amend it according to the relevant comments outlined here.

* Please cite your Supporting Information as outlined here: https://journals.plos.org/plosmedicine/s/supporting-information

REFERENCES

* PLOS uses the numbered citation (citation-sequence) method and first six authors, et al.

* Please ensure that journal name abbreviations match those found in the National Center for Biotechnology Information (NCBI) databases (http://www.ncbi.nlm.nih.gov/nlmcatalog/journals), and are appropriately formatted and capitalised.

* Where website addresses are cited, please include the complete URL and specify the date of access (e.g. [accessed: 12/06/2023]).

* Please also see https://journals.plos.org/plosmedicine/s/submission-guidelines#loc-references for further details on reference formatting.

OBSERVATIONAL STUDIES

* Abstract: Please include the study design, population and setting, number of participants, years during which the study took place (enrollment and follow up), length of follow up, and main outcome measures.

* Please ensure that the study is reported according to the STROBE (or appropriate STOBE extension) guideline (available from: https://www.equator-network.org/reporting-guidelines/strobe) and include the completed STROBE (or STROBE extension) checklist as Supporting Information. Please add the following statement, or similar, to the Methods: "This study is reported as per the Strengthening the Reporting of Observational Studies in Epidemiology (STROBE) guideline (S1 Checklist)." When completing the checklist, please use section and paragraph numbers, rather than page numbers.

* For all observational studies, in the manuscript text, please indicate: (1) the specific hypotheses you intended to test, (2) the analytical methods by which you planned to test them, (3) the analyses you actually performed, and (4) when reported analyses differ from those that were planned, transparent explanations for differences that affect the reliability of the study's results. If a reported analysis was performed based on an interesting but unanticipated pattern in the data, please be clear that the analysis was data driven.

* Please state in the Methods section whether the study had a prospective protocol or analysis plan. If a prospective analysis plan (from your funding proposal, IRB or other ethics committee submission, study protocol, or other planning document written before analyzing the data) was used in designing the study, please include the relevant document(s) with your revised manuscript as a Supporting Information file to be published alongside your study and cite it in the Methods section. A legend for this file should be included at the end of your manuscript. If no such document exists, please make sure that the Methods section transparently describes when analyses were planned, and when/why any data-driven changes to analyses took place. Changes in the analysis, including those made in response to peer review comments, should be identified as such in the Methods section of the paper, with rationale.

# Author comment: Author response to Decision Letter 3

**DOI:** 10.1371/journal.pmed.1004785.r005

1 Apr 2026

Attachment Submitted filename: Authors response_second review_APR.docx

# Peer review: Decision Letter 3

**DOI:** 10.1371/journal.pmed.1004785.r006

**Authors:** Andreia Cunha

13 May 2026

Dear Dr. Manyara,

Thank you very much for re-submitting your manuscript "Hypertension and diabetes prevalence, associated factors, care cascade, and quality of life in older adults: cross-sectional population-based study in The Gambia, South Africa, and Zimbabwe" (PMEDICINE-D-25-03448R3) for review by PLOS Medicine.

I have discussed the paper with my colleagues and it was also seen again by two original reviewers. I am pleased to say that provided the remaining editorial and production issues are dealt with we are planning to accept the paper for publication in the journal.

The remaining issues that need to be addressed are listed at the end of this email. Any accompanying reviewer attachments can be seen via the link below. Please take these into account before resubmitting your manuscript:

[LINK]

***Please note while forming your response, if your article is accepted, you may have the opportunity to make the peer review history publicly available. The record will include editor decision letters (with reviews) and your responses to reviewer comments. If eligible, we will contact you to opt in or out.***

In revising the manuscript for further consideration here, please ensure you address the specific points made by each reviewer and the editors. In your rebuttal letter you should indicate your response to the reviewers' and editors' comments and the changes you have made in the manuscript. Please submit a clean version of the paper as the main article file. A version with changes marked must also be uploaded as a marked up manuscript file.

Please also check the guidelines for revised papers at http://journals.plos.org/plosmedicine/s/revising-your-manuscript for any that apply to your paper. If you haven't already, we ask that you provide a short, non-technical Author Summary of your research to make findings accessible to a wide audience that includes both scientists and non-scientists. The Author Summary should immediately follow the Abstract in your revised manuscript. This text is subject to editorial change and should be distinct from the scientific abstract.

In addition to these revisions, you may need to complete some formatting changes, which you will receive in a follow up email. A member of our team will be in touch with a set of requests shortly. If you do not receive a separate email within a few days, please assume that checks have been completed, and no additional changes are required.

We expect to receive your revised manuscript within 1 week. Please email us (plosmedicine@plos.org) if you have any questions or concerns.

We ask every co-author listed on the manuscript to fill in a contributing author statement. If any of the co-authors have not filled in the statement, we will remind them to do so when the paper is revised. If all statements are not completed in a timely fashion this could hold up the re-review process. Should there be a problem getting one of your co-authors to fill in a statement we will be in contact. YOU MUST NOT ADD OR REMOVE AUTHORS UNLESS YOU HAVE ALERTED THE EDITOR HANDLING THE MANUSCRIPT TO THE CHANGE AND THEY SPECIFICALLY HAVE AGREED TO IT.

Please ensure that the paper adheres to the PLOS Data Availability Policy (see http://journals.plos.org/plosmedicine/s/data-availability), which requires that all data underlying the study's findings be provided in a repository or as Supporting Information. For data residing with a third party, authors are required to provide instructions with contact information for obtaining the data. PLOS journals do not allow statements supported by "data not shown" or "unpublished results." For such statements, authors must provide supporting data or cite public sources that include it.

To enhance the reproducibility of your results, we recommend that you deposit your laboratory protocols in protocols.io, where a protocol can be assigned its own identifier (DOI) such that it can be cited independently in the future. Additionally, PLOS ONE offers an option to publish peer-reviewed clinical study protocols. Read more information on sharing protocols at https://plos.org/protocols?utm_medium=editorial-email&utm_source=authorletters&utm_campaign=protocols

Please review your reference list to ensure that it is complete and correct. If you have cited papers that have been retracted, please include the rationale for doing so in the manuscript text, or remove these references and replace them with relevant current references. Any changes to the reference list should be mentioned in the rebuttal letter that accompanies your revised manuscript.

Please note, when your manuscript is accepted, an uncorrected proof of your manuscript will be published online ahead of the final version, unless you've already opted out via the online submission form. If, for any reason, you do not want an earlier version of your manuscript published online or are unsure if you have already indicated as such, please let the journal staff know immediately at plosmedicine@plos.org.

If you have any questions in the meantime, please contact me or the journal staff on plosmedicine@plos.org.

We look forward to receiving the revised manuscript by May 20 2026 11:59PM.

Sincerely,

Andreia Cunha, PhD

Senior Editor

PLOS Medicine

plosmedicine.org

------------------------------------------------------------

Requests from Editors:

GENERAL EDITORIAL REQUESTS

* Please confirm that your title complies with PLOS Medicine's style. Your title must be nondeclarative and not a question. It should begin with main concept if possible. "Effect of" should be used only if causality can be inferred, i.e., for an RCT. Please place the study design ("A randomized controlled trial," "A retrospective study," "A modelling study," etc.) in the subtitle (ie, after a colon).

* Please confirm that your abstract complies with our requirements, including format (three sections: Background, Methods and Findings, and Conclusions) and providing all the information relevant to this study type https://journals.plos.org/plosmedicine/s/submission-guidelines#loc-abstract

* In the abstract, please include the important dependent variables that are adjusted for in the analyses.

* Please ensure that the Introduction ends with a clear description of the study question or hypothesis.

* Please ensure that all abbreviations are defined at first use throughout the text.

* Please confirm that all numbers presented in the abstract are present and identical to numbers presented in the main manuscript text.

* Statistical reporting: Please revise throughout the manuscript, including tables and figures.

- Please report statistical information as follows to improve clarity for the reader (95% CI [13,28]; p</=).

- Please separate upper and lower bounds with commas instead of hyphens as the latter can be confused with reporting of negative values.

- Please repeat statistical definitions (HR, CI etc.) for each set of parentheses.

GENERAL

FUNDING STATEMENT

* The funding statement should include: specific grant numbers, initials of authors who received each award, URLs to sponsors’ websites. Also, please state whether any sponsors or funders (other than the named authors) played any role in study design, data collection and analysis, the decision to publish, or preparation of the manuscript. If they had no role in the research, include this sentence: “The funders had no role in study design, data collection and analysis, decision to publish, or preparation of the manuscript.”

* It appears that one or more study authors may be affiliated with one or more of the agencies that funded the study (i.e. MRC) Thus, the statement “The funders had no role in study design, data collection and analysis, decision to publish, or preparation of the manuscript” does not apply. Please revise the Financial Disclosure accordingly, as in "[Author name] is [author's role] at [funding agency]. The funders had no other role in study design…..”

COMPETING INTERESTS STATEMENT

* All authors must declare their relevant competing interests per the PLOS policy, which can be seen here: https://journals.plos.org/plosmedicine/s/competing-interests For authors with ties to industry, please indicate whether any of the interests has a financial stake in the results of the current study.

*Please amend your competing interests statement. Funding information should go to the relevant section and include URLs.

DATA AVAILABILITY

* PLOS Medicine requires that the de-identified data underlying the specific results in a published article be made available, without restrictions on access, in a public repository or as Supporting Information at the time of article publication, provided it is legal and ethical to do so. Please see the policy at

http://journals.plos.org/plosmedicine/s/data-availability

and FAQs at

http://journals.plos.org/plosmedicine/s/data-availability#loc-faqs-for-data-policy

* PLOS defines the “minimal data set” to consist of the data set used to reach the conclusions drawn in the manuscript with related metadata and methods, and any additional data required to replicate the reported study findings in their entirety. Authors do not need to submit their entire data set, or the raw data collected during an investigation. Please submit the following data:

The values behind the means, standard deviations and other measures reported;

The values used to build graphs;

The points extracted from images for analysis.

* The Data Availability Statement (DAS) requires revision. For each data source used in your study:

a) If the data are freely or publicly available, note this and state the location of the data: within the paper, in Supporting Information files, or in a public repository (include the DOI or accession number).

b) If the data are owned by a third party but freely available upon request, please note this and state the owner of the data set and contact information for data requests (web or email address). Note that a study author cannot be the contact person for the data.

c) If the data are not freely available, please describe briefly the ethical, legal, or contractual restriction that prevents you from sharing it. Please also include an appropriate contact (web or email address) for inquiries (again, this cannot be a study author).

ETHICS AND CONSENT

* Please provide the name(s) of the institutional review board(s) that provided ethical approval in the UK (if relevant).

* Please provide more information about the assent process through a

proxy for those with cognitive impairment. In particular, who was the proxy and how was cognitive impairment determined. Please ensure that the research complies with the PLOS policy in full: https://journals.plos.org/plosmedicine/s/human-subjects-research#loc-patient-privacy-and-informed-consent-for-publication

FIGURES

* Please provide titles and legends for all figures and tables (including those in Supporting Information files). Please define all acronyms used in each figure or table in its corresponding legend.

* Please ensure that where relevant figures include 95% CIs.

* When a p value is given, please specify the statistical test used to determine it in the legend.

* Please consider avoiding the use of red and green in order to make your figure more accessible

* Please provide the unadjusted comparisons as well as the adjusted comparisons in all relevant Tables

* Please specify the variables controlled for in all relevant Tables

INCLUSIVITY IN GLOBAL RESEARCH

PLOS has a 'Inclusivity in Global Research' policy which aims to promote collaboration and inclusivity in global health research. You are required to complete PLOS’ questionnaire on inclusivity in global research and submit it with your revised paper. The policy and questionnaire can be found at https://journals.plos.org/plosone/s/best-practices-in-research-reporting.

OBSERVATIONAL, COHORT, CROSS-SECTIONAL, AND CASE CONTROL STUDIES

* Please ensure that the study is reported according to the STROBE guideline, and include the completed STROBE checklist as Supporting Information. Please add the following statement, or similar, to the Methods: ""This study is reported as per the Strengthening the Reporting of Observational Studies in Epidemiology (STROBE) guideline (S1 Checklist).""

The STROBE guideline can be found here: http://www.equator-network.org/reporting-guidelines/strobe/

When completing the checklist, please use section and paragraph numbers, rather than page numbers.

* Did your study have a prospective protocol or analysis plan? Please state this (either way) early in the Methods section.

a) If a prospective analysis plan (from your funding proposal, IRB or other ethics committee submission, study protocol, or other planning document written before analyzing the data) was used in designing the study, please include the relevant prospectively written document with your revised manuscript as a Supporting Information file to be published alongside your study, and cite it in the Methods section. A legend for this file should be included at the end of your manuscript.

b) If no such document exists, please make sure that the Methods section transparently describes when analyses were planned, and when/why any data-driven changes to analyses took place.

c) In either case, changes in the analysis-- including those made in response to peer review comments-- should be identified as such in the Methods section of the paper, with rationale."

* Your study is observational and therefore causality cannot be inferred. Please remove language that implies causality and refer to associations instead.

* For all observational studies, in the manuscript text, please indicate: (1) the specific hypotheses you intended to test, (2) the analytical methods by which you planned to test them, (3) the analyses you actually performed, and (4) when reported analyses differ from those that were planned, transparent explanations for differences that affect the reliability of the study's results. If a reported analysis was performed based on an interesting but unanticipated pattern in the data, please be clear that the analysis was data-driven.

Comments from Reviewers:

Reviewer #3: I thank the authors for addressing my remaining comments comprehensively. I have no further comments that require my re-review.

A very minor thing that the authors may want to check: in the statement "While analyses were pre-planned, the statistical analysis plan was not pre-specified", I wonder whether the authors had intended the last word to be "published" rather than "pre-specified"? If the analyses were pre-planned, then there would have been a pre-specified SAP - it's just that that was not deposited externally, I assume?

Reviewer #4: I thank the authors for their responses to my comments (I was reviewer 4 in the previous round). I have no further comments.

Any attachments provided with reviews can be seen via the following link:

[LINK]

# Author comment: Author response to Decision Letter 4

**DOI:** 10.1371/journal.pmed.1004785.r007

18 May 2026

Attachment Submitted filename: Minor revision response.docx

# Editor report: Decision Letter 4

**DOI:** 10.1371/journal.pmed.1004785.r008

**Authors:** Andreia Cunha

26 May 2026

Dear Dr Manyara,

On behalf of my colleagues and the Academic Editor, Jennifer Manne-Goehler, I am pleased to inform you that we have agreed to publish your manuscript "Hypertension and diabetes prevalence, associated factors, care cascade, and quality of life in older adults: cross-sectional population-based study in The Gambia, South Africa, and Zimbabwe" (PMEDICINE-D-25-03448R4) in PLOS Medicine.

Before your manuscript can be formally accepted you will need to complete some formatting changes, which you will receive in a follow up email. Please be aware that it may take several days for you to receive this email; during this time no action is required by you. Once you have received these formatting requests, please note that your manuscript will not be scheduled for publication until you have made the required changes.

In the meantime, please log into Editorial Manager at http://www.editorialmanager.com/pmedicine/, click the "Update My Information" link at the top of the page, and update your user information to ensure an efficient production process.

PRESS

We frequently collaborate with press offices. If your institution or institutions have a press office, please notify them about your upcoming paper at this point, to enable them to help maximise its impact. If the press office is planning to promote your findings, we would be grateful if they could coordinate with medicinepress@plos.org. If you have not yet opted out of the early version process, we ask that you notify us immediately of any press plans so that we may do so on your behalf.

We also ask that you take this opportunity to read our Embargo Policy regarding the discussion, promotion and media coverage of work that is yet to be published by PLOS. As your manuscript is not yet published, it is bound by the conditions of our Embargo Policy. Please be aware that this policy is in place both to ensure that any press coverage of your article is fully substantiated and to provide a direct link between such coverage and the published work. For full details of our Embargo Policy, please visit http://www.plos.org/about/media-inquiries/embargo-policy/.

To enhance the reproducibility of your results, we recommend that you deposit your laboratory protocols in protocols.io, where a protocol can be assigned its own identifier (DOI) such that it can be cited independently in the future. Additionally, PLOS ONE offers an option to publish peer-reviewed clinical study protocols. Read more information on sharing protocols at https://plos.org/protocols?utm_medium=editorial-email&utm_source=authorletters&utm_campaign=protocols

Thank you again for submitting to PLOS Medicine. We look forward to publishing your paper.

Sincerely,

Andreia Cunha, PhD

Senior Editor

PLOS Medicine
